# BMPR2 acts as a gatekeeper to protect endothelial cells from increased TGFβ responses and altered cell mechanics

**DOI:** 10.1371/journal.pbio.3000557

**Published:** 2019-12-11

**Authors:** Christian Hiepen, Jerome Jatzlau, Susanne Hildebrandt, Branka Kampfrath, Melis Goktas, Arunima Murgai, Jose Luis Cuellar Camacho, Rainer Haag, Clemens Ruppert, Gerhard Sengle, Elisabetta Ada Cavalcanti-Adam, Kerstin G. Blank, Petra Knaus

**Affiliations:** 1 Freie Universität Berlin, Institute for Chemistry and Biochemistry, Berlin, Germany; 2 Berlin-Brandenburg School for Regenerative Therapies, Charité Universitätsmedizin Berlin, Germany; 3 Max Planck Institute of Colloids and Interfaces, Mechano(bio)chemistry, Potsdam, Germany; 4 Max Planck Institute for Molecular Genetics, Berlin, Germany; 5 Universities of Giessen and Marburg Lung Center (UGMLC), Medical Clinic II, Justus Liebig University, Giessen, Germany; 6 University of Cologne, Center for Biochemistry, Medical Faculty, Center for Molecular Medicine Cologne (CMMC), Cologne, Germany; 7 Max Planck Institute for Medical Research, Cellular Biophysics, Department of Physical Chemistry, Heidelberg, Germany; University of Pennsylvania School of Medicine, UNITED STATES

## Abstract

Balanced transforming growth factor-beta (TGFβ)/bone morphogenetic protein (BMP)-signaling is essential for tissue formation and homeostasis. While gain in TGFβ signaling is often found in diseases, the underlying cellular mechanisms remain poorly defined. Here we show that the receptor BMP type 2 (BMPR2) serves as a central gatekeeper of this balance, highlighted by its deregulation in diseases such as pulmonary arterial hypertension (PAH). We show that BMPR2 deficiency in endothelial cells (ECs) does not abolish pan-BMP-SMAD1/5 responses but instead favors the formation of mixed-heteromeric receptor complexes comprising BMPR1/TGFβR1/TGFβR2 that enable enhanced cellular responses toward TGFβ. These include canonical TGFβ-SMAD2/3 and lateral TGFβ-SMAD1/5 signaling as well as formation of mixed SMAD complexes. Moreover, BMPR2-deficient cells express genes indicative of altered biophysical properties, including up-regulation of extracellular matrix (ECM) proteins such as fibrillin-1 (FBN1) and of integrins. As such, we identified accumulation of ectopic FBN1 fibers remodeled with fibronectin (FN) in junctions of BMPR2-deficient ECs. Ectopic FBN1 deposits were also found in proximity to contractile intimal cells in pulmonary artery lesions of BMPR2-deficient heritable PAH (HPAH) patients. In BMPR2-deficient cells, we show that ectopic FBN1 is accompanied by active β1-integrin highly abundant in integrin-linked kinase (ILK) mechano-complexes at cell junctions. Increased integrin-dependent adhesion, spreading, and actomyosin-dependent contractility facilitates the retrieval of active TGFβ from its latent fibrillin-bound depots. We propose that loss of BMPR2 favors endothelial-to-mesenchymal transition (EndMT) allowing cells of myo-fibroblastic character to create a vicious feed-forward process leading to hyperactivated TGFβ signaling. In summary, our findings highlight a crucial role for BMPR2 as a gatekeeper of endothelial homeostasis protecting cells from increased TGFβ responses and integrin-mediated mechano-transduction.

## Abstract 2

Balanced TGFβ/BMP-signaling is essential for tissue formation and homeostasis; this study shows that the receptor BMPR2 serves as gatekeeper of this balance, protecting cells from increased TGFβ-responses and integrin-mediated mechanotransduction.

## Introduction

Tissue homeostasis involves tight regulation and coordination of biochemical and biomechanical signaling pathways to maintain cellular identity and functionality. Balance between bone morphogenetic protein (BMP) and transforming growth factor-beta (TGFβ) signaling is equally important for tissue homeostasis. For vascular homeostasis, sustained activity of BMP but only mild TGFβ signaling is required [1]. A switch of this balance to the benefit of augmented responses toward TGFβ is a hallmark of endothelial cell (EC) dysfunction and precondition to several vascular diseases, including cerebral cavernous malformation (CCM) [2], hereditary hemorrhagic telangiectasia (HHT) [3], and pulmonary arterial hypertension (PAH). Understanding the cellular context in which this imbalance takes place is key to tackling augmented TGFβ signaling [4]. Endothelial responses to TGFβ include extracellular matrix (ECM) production and endothelial-to-mesenchymal transition (EndMT), a process by which ECs lose their identity and instead adopt a mesenchymal/myo-fibroblastic character [5,6]. PAH is a rare and lethal vascular disorder affecting small pulmonary arteries [7–9]. Mutations in BMP type-2 receptor (*BMPR2*) represent the primary heritable risk factor for PAH development [10], with loss of functional BMPR2 expression an underlying molecular cause [11]. Endothelial BMPR2 deficiency is also found in some but not all cases of idiopathic PAH (IPAH) [12,13] as well as in other vascular pathologies involving endothelial inflammation and arteriosclerosis [14] reviewed in [15].

While BMPR2 deficiency would naturally imply reduction of pan-BMP- Suppressor of Mothers against Decapentaplegic (SMAD)1/5 signaling, several reports on BMPR2-deficient cells, including ECs, show that this cannot be seen as a generalized paradigm and strongly depends on specific BMP ligands used in the respective study [16,17]. Instead, more conclusive results in BMPR2-deficient cells exist in respect to gain in TGFβ-SMAD signaling [16,18], reviewed in Rol and colleagues [9]. Together, this suggests that BMPR2 acts as a central gatekeeper to protect ECs from dysfunction and possibly also increased TGFβ signaling by molecular mechanisms that remain poorly defined. TGFβ/BMP signaling is understood to regulate important mechanobiological aspects of the cell. One of the best understood processes is the release of mature TGFβ1 from its latent ECM tethered complex by integrins. This mechanically driven mechanism is enhanced with higher cell forces and ECM stiffening [19,20]. Particularly, myo-fibroblasts that contribute to a fibrotic process are suited to perform this activation mechanism very efficiently [21]. Histopathological features of PAH lesions—such as contractile phenotype, excessive ECM remodeling, and disturbed tissue architecture [22–24]—urged us to investigate whether there is a connection between BMPR2 deficiency and increased TGFβ signaling and how this relates to alterations in mechano-biology and ECM biology and EndMT.

Access to primary human BMPR2-deficient cells is very limited. Using CRISPR/Cas9, we created 2 human EC lines carrying monoallelic mutations in *BMPR2* leading to endothelial BMPR2 deficiency. Both mutations were reported to induce PAH-associated phenotypes in humans and mice [10,25,26]. The majority of *BMPR2* mutations found in humans are non-sense or frame-shift mutations leading to non-sense–mediated decay (NMD) of the RNA transcript. Some more clinically severe outcomes occur in patients with *BMPR2* mutations bypassing NMD but instead result in misfolded proteins that mislocalize intracellularly [27,28]. In either case, BMPR2 deficiency is established by lack of functional BMPR2 expression at the cell surface, as the expression from the remaining wild-type (WT) allele is low, which adds up to the reported haploinsufficiency for *BMPR2* mutations [11]. Combining biochemical and biophysical methods, we reveal that BMPR2 deficiency favors formation of mixed-heteromeric complexes comprising BMPR1, TGFβR1, and TGFβR2 receptors. As a consequence, SMAD signaling is altered, and genes required for cellular mechanics are up-regulated. Strikingly, BMPR2-deficient ECs undergo EndMT and recapitulate mechanical features such as stiffening at EC junctions [29]. Increased cell-generated forces promote the local retrieval of active TGFβ from ECM depots, a process dependent on specific integrins, ECM composition, and contractility [19,20,30]. Our study highlights the importance of BMPR2 for limiting TGFβ responses in ECs. This mechanism is relevant for a better understanding of EC dysfunction during vascular pathologies, particularly when ECs adopt a myo-fibrotic character.

## Results

BMP signaling requires a complex of type-1 (BMPR1, i.e., Activin receptor-like kinase [ALK]-1, 2, 3, or 6) and type-2 (i.e., BMPR2, Activin A receptor, type-2 A [ACVR2A]/Activin A receptor, type-2 B [ACVR2B]) serine/threonine kinase receptors to activate SMAD transcription factors. BMPR2, a constitutively active kinase receptor, oligomerizes with BMPR1 to phosphorylate SMAD1/5/8 upon ligand binding. In the quiescent vasculature, the main endothelial BMP-SMAD1/5 signals are induced by BMP9/10 found in human plasma [31,32], with BMP9/10 binding to its high-affinity receptor ALK1 in complex with BMPR2 and co-receptor Endoglin (ENG) [33–35]. In ECs, TGFβ induces SMAD2/3 signaling via binding to its high-affinity receptor TGFβR2 (TβR2) in complex with TGFβR1 (ALK5) [36] and co-receptor ENG [37]. Moreover, TGFβ was shown to induce “lateral” activation of SMAD1/5 via complexes comprising TβR2, ALK5, and BMP type-1 receptors ALK1 in endothelial cells [36,38] and ALK2/3 in epithelial cells [38–41]. We developed 2 human BMPR2-deficient EC models via CRISPR-Cas9 genome editing (S1A Fig). Cells expressing a single copy of extracellularly truncated BMPR2 (herein referred to as BMPR2^ΔE2^) were generated by splice-site deletion causing exon2 skipping. This mutation was found before to escape NMD and to mislocalize intracellularly [27,41]. Cells deleted for a single copy of *BMPR2* (herein referred to as BMPR2^KO^) were generated by Cas9-induced frameshift-mutation causing NMD of the RNA transcript. Validations on genome and protein levels of the editing approach are described in S1A–S1E Fig. Even though both mutations were monoallelic, both editing approaches were leading to strong reduction of expression as well as surface levels of WT BMPR2, as detected by western blotting (WB) (S1E Fig) and surface biotinylation experiments (S1F Fig). The mutation BMPR2^ΔE2^ gave rise to a truncated protein (S1C Fig) with low cell surface abundance (S1F Fig) but accumulated in the endoplasmic reticulum (S1G Fig), in agreement with previous reports on this mutation [27].

### BMPR2-deficient ECs gain canonical TGFβ-SMAD2/3 and lateral TGFβ-SMAD1/5 responsiveness

**Fig 1:**
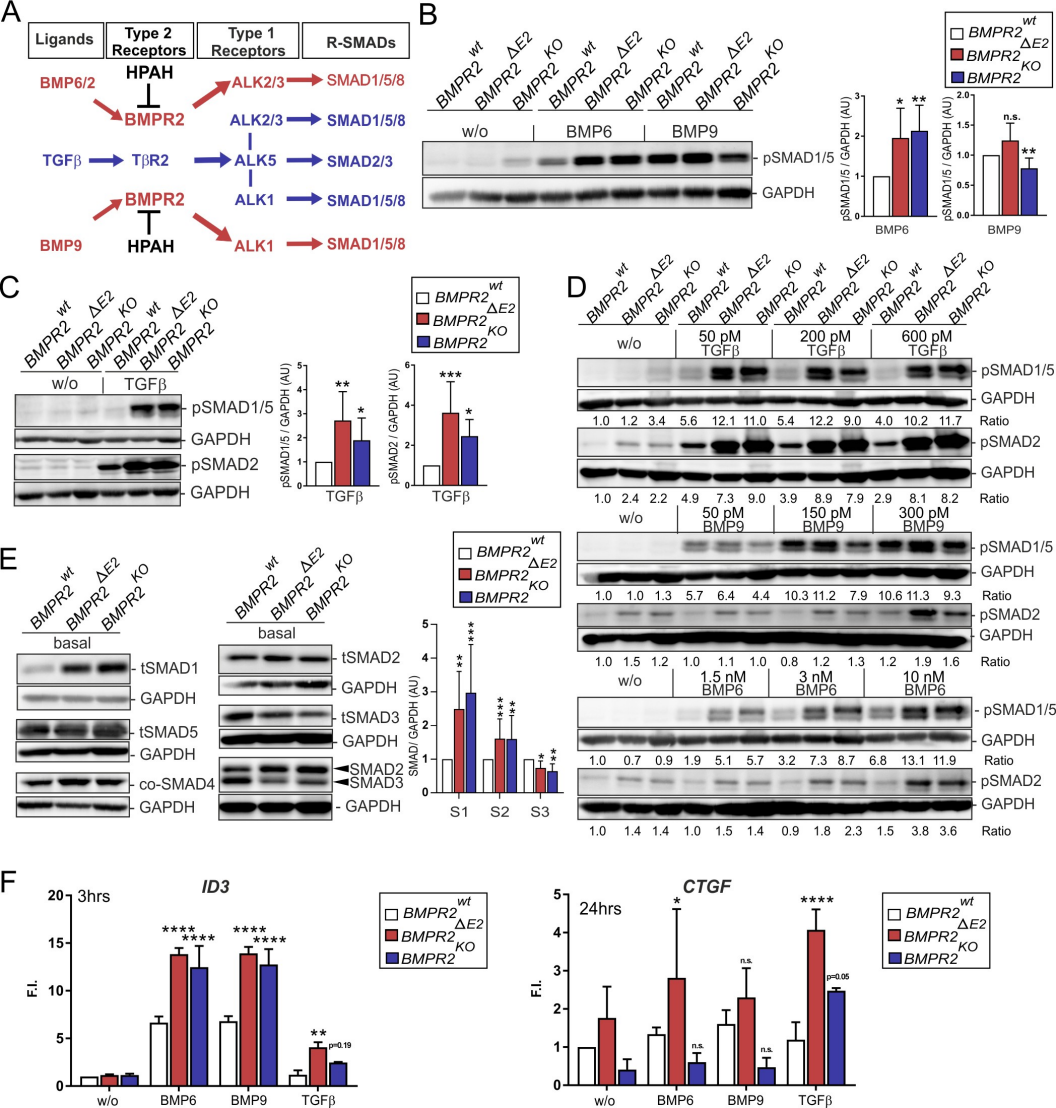
BMPR2-deficient ECs gain canonical TGFβ-SMAD2/3 and lateral TGFβ-SMAD1/5 responsiveness. (A) EC homeostasis is controlled by balanced TGFβ and BMP signaling. Loss of BMPR2 (black) leads to unbalanced TGFβ/BMP signaling. BMP6/2 activate ECs via BMPR2-ALK2 and BMPR2-ALK3 signaling complexes, respectively, to induce angiogenesis. Receptor complexes of TβR2 with either ALK5 alone (blue arrow), with ALK5 and ALK1, or with ALK5 and ALK2/3 (blue lines) have been described. TGFβ-induced activation of SMAD1/5/8 via such a complex is termed “lateral TGFβ signaling.” BMP9 acts via ALK1-SMAD1/5/8 signaling as a vascular quiescence factor important to maintaining vessel integrity. (B) Immunoblot using an antibody specific to pSMAD1/5. Cells were treated with BMP6 (3 nM) or BMP9 (0.3 nM) for 15 min. Densitometric quantification (right) of pSMAD1/5 relative to GAPDH levels expressed as AUs (*n* = 7 independent experiments). (C) Immunoblot (left) using antibodies specific to pSMAD1/5 or pSMAD2. Cells were treated with TGFβ (200 pM) for 15 min. Quantification of pSMAD1/5 and pSMAD2 signal intensity relative to GAPDH levels (right) expressed as AUs (*n* = 6 independent experiments). (D) Immunoblot showing dose responses for TGFβ (50 pM, 200 pM, 600 pM), BMP9 (50 pM, 150 pM, 300 pM), or BMP6 (1.5 nM, 3 nM, 10 nM) after 15 min of stimulation. The ratios of signal intensities are shown below each panel. (E) Total protein levels under steady-state growth conditions of indicated SMADs. Quantification of tSMAD1–3 signal intensity relative to GAPDH levels (right) expressed as AUs (*n* = 8–12 independent experiments). (F) qRT-PCR (6 h of starvation and 3 h [for ID3] or 24 h [for CTGF] stimulation) with indicated ligands. Values are expressed as F.I. (*n* = 3 independent experiments). In all panels, the data are shown as mean + SD relative to BMPR2^wt^. Statistical significance relative to BMPR2^wt^ was calculated using Kruskal-Wallis test with post hoc Dunn test for densitometric quantifications and two-way ANOVA and Bonferroni post hoc test for RT data. *P < 0.05, **P < 0.01, ***P < 0.001, ****P < 0.0001. See also S2 Fig and S1 Data for underlying data. ALK2, ; AU, arbitrary unit; BMP, bone morphogenetic protein; BMPR2, BMP type-2 receptor; EC, endothelial cell; F.I., fold induction; n.s., not significant; pSMAD, ; qRT-PCR, quantitative real-time PCR; RT, real time; SMAD, Suppressor of Mothers against Decapentaplegic; TβR2, TGFβ receptor 2; TGFβ, transforming growth factor-beta; tSMAD, total SMAD.

To characterize SMAD activation in *BMPR2-*deficient cells, we stimulated ECs with either BMP6 or BMP9, ligands with distinct receptor-complex–binding affinities and vascular functions (Fig 1A and 1B).

BMP6 signals through complexes comprising ALK2/3 with BMPR2 or ACTR2A/B to activate ECs and to regulate angiogenesis [42]. BMP9 instead acts as vascular quiescence factor signaling through complexes comprising BMPR2 and ALK1 [43–45]. Interestingly, stimulation with BMP6 leads to significant increase in SMAD1/5 phosphorylation in cells lacking BMPR2 (Fig 1B), whereas BMP9 stimulation is not altered in BMPR2^ΔE2^ mutant cells—it is down-regulated in BMRR2^KO^ cells (Fig 1B). This implies distinct involvement of the BMP type-1 receptors ALK2 (for BMP6) and ALK1 (for BMP9) in the mutant cells.

As expected, both BMPR2-deficient EC lines gained strong TGFβ responsiveness indicated by increased SMAD2 phosphorylation, as well as significantly more TGFβ-dependent SMAD1/5 phosphorylation (Fig 1C). In our system, the TGFβ-induced lateral activation of SMAD1/5 signaling appears to be dose independent, while both BMP9- and BMP6-mediated SMAD1/5 phosphorylation still depend on ligand concentrations used here (Fig 1D). This suggests that, in BMPR2-deficient cells, there is an increased number of receptor complexes able to facilitate lateral TGFβ responses. Because saturation in pSMAD1/5 activation is achieved with low doses of TGFβ in all cells, investigated abundancy of the former complexes in comparison to signaling complexes facilitating BMP6/9-induced pSMAD1/5 appears low. The same dose independency for lateral SMAD1/5 signaling was observed for Activin A, a member of the same ligand family (S2A Fig). Interestingly, protein levels of Receptor-regulated SMADs (R-SMADs) were differentially affected and SMAD1 was strongly and SMAD2 mildly increased, whereas SMAD5 was unaltered and SMAD3 decreased (Fig 1E). This increase in BMP- and TGFβ-dependent activation of SMADs is also recapitulated by the transcriptional regulation of target genes. Here, increased expression of the SMAD1/5 target gene *ID-3* upon BMP6, BMP9, and importantly also TGFβ stimulation can be observed in BMPR2-deficient cells (Fig 1F). SMAD2/3 target gene *CTGF* is stronger induced by TGFβ in *BMPR2*-deficient ECs, while there is only mild response on this gene by BMP6 or BMP9 stimulation of BMPR2^ΔE2^ mutant cells (Fig 1F). Taken together, two mutant *BMPR2*-deficient cell models confirm a gain in BMP, TGFβ, and Activin A responsiveness, with increased lateral TGFβ signaling responses as demonstrated by both increased SMAD1/5 phosphorylation and target gene responses.

### BMPR2 deficiency increases heteromerization of BMP and TGFβ receptors also indicated by increased formation of mixed SMAD complexes

**Fig 2:**
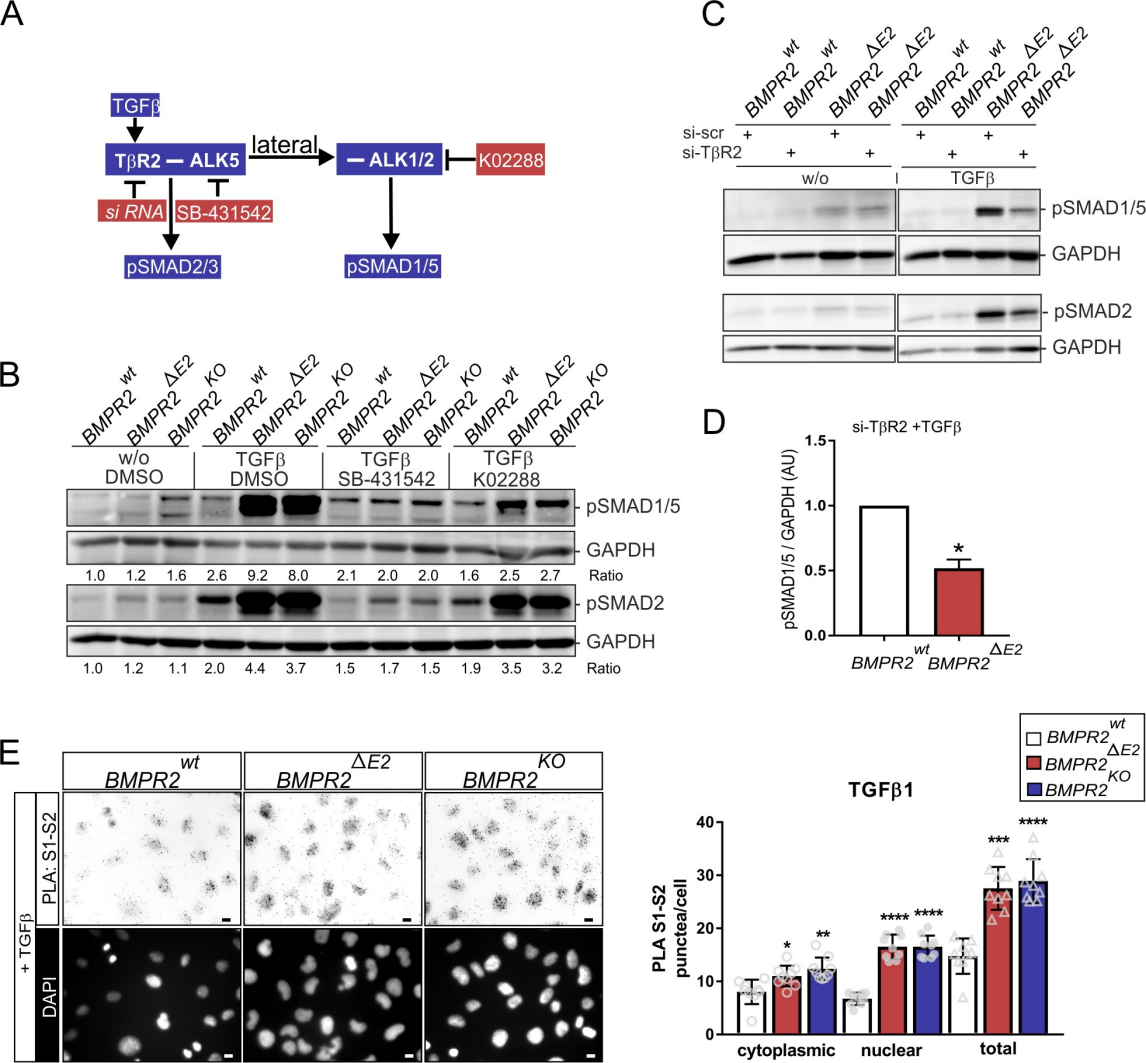
BMPR2-deficient ECs signal through heteromers comprising BMP and TGFβ receptors as indicated by the formation of mixed SMAD complexes. (A) Scheme depicting the targeting conditions for TGFβ-induced SMAD1/5 and SMAD2/3 signaling, i.e., inhibition of TβR2-ALK5 and heteromeric TβR2-ALK5-ALK1/ALK2 complexes by selective small molecules K02288 against ALK2/ALK1 or SB-431542 against ALK5, or siRNA targeting TβR2. (B) Immunoblot using antibodies specific to pSMAD1/5 or pSMAD2 showing responses upon 15 min TGFβ stimulation (200 pM) with a 1-h pre-exposure to K02288 (0.5 μM), SB-431542 (5 μM), or DMSO. (C) Effects of TβR2 knock-down by specific siRNA compared to a scrambled control. (D) Densitometric quantification of pSMAD1/5 relative to GAPDH levels expressed as AUs (*n* = 4 independent experiments). Note the significant reduction in lateral TGFβ signaling on the level of pSMAD1/5 phosphorylation (right) when TβR2 levels are reduced. (E) Epifluorescence images of PLA (left) showing complexes of SMAD1 (S1) with SMAD2 (S2) in indicated cell clones upon TGFβ stimulation (200 pM) for 15 min. PLA signals are pseudo-colored greyscale and inverted (upper). Scale bar, 10 μm. Quantification of SMAD1-SMAD2 PLA signals (right) in TGFβ-stimulated cells with the number of nuclear, cytosolic, and overall PLA foci shown. Data are presented as mean ± SD (*n* = 9 frames, 20–30 cells each). Statistical significance relative to BMPR2^wt^ was calculated using two-tailed Mann-Whitney test for densitometric quantification and one-way ANOVA and Bonferroni post hoc test for PLA data. *P < 0.05, **P < 0.01, ***P < 0.001, ****P < 0.0001. See also S3 Fig and S2 Data for underlying data. ALK5, activin receptor-like kinase 5; AU, arbitrary unit; BMP, bone morphogenetic protein; BMPR2, BMP type-2 receptor; EC, endothelial cell; PLA, proximity ligation assay; pSMAD, phosphorylated SMAD; siRNA, small interfering RNA; SMAD, suppressor of mothers against decapentaplegic; TβR2, TGFβ type-2 receptor; TGFβ, transforming growth factor-beta; tSMAD, total SMAD.

We next investigated the receptor complexes involved in the increased lateral TGFβ signaling of BMPR2-deficient cells using small interfering RNA (siRNA)-based knock-down targeting the high-affinity TβR2 (S3A Fig), small-molecule kinase inhibitors (SMKIs; selective for ALK5 [SB-431542]) [46,47], or BMP type-1 receptors ALK2 and ALK1 (K02288) [48,49] (Fig 2A).

Inhibition of ALK5 with SB-431542 efficiently blocked TGFβ-induced SMAD1/5 phosphorylation and canonical SMAD2 phosphorylation (Fig 2B) without interfering with BMP-induced SMAD1/5 phosphorylation in BMPR2-deficient ECs (S3B Fig). Inhibition with K02288 also efficiently reduced TGFβ-induced SMAD1/5 phosphorylation in BMPR2-deficient cells (Fig 2B). Moreover, knock-down of TβR2 strongly reduced TGFβ-induced SMAD1/5 phosphorylation (Fig 2C and 2D). Since individual type-1 receptors carry intrinsic specificities for respective R-SMADs [50], mixed heteromerization of BMP type-1 receptors with ALK5 and TβR2 may lead to formation of mixed SMAD complexes. Indeed, it was previously reported that lateral signaling by TGFβ is also likely to gain increased mixed SMAD complex formation, and these complexes appear with higher abundance in tissues with high levels of TGFβ [51,52]. However, it is not clear yet to what extent these mixed SMAD complexes retain signaling competence [39] and are able to bind to classical BMP target genes [40]. To explore whether increased formation of mixed SMAD complexes would be indicative of an increased mixed heteromerization between BMPR1 and TGFβR1 in BMPR2-deficient cells, we performed proximity ligation assay (PLA). PLAs were performed for the combination of SMAD1 and SMAD2 (Fig 2E) as well as SMAD5 and SMAD2/3 (S3C Fig) using specific antibodies (S3D Fig and S2E Fig) respectively. For TGFβ-stimulated cells, the combinatorial SMAD1-SMAD2 antibody approach showed increased complex formation and increased appearance of SMAD1-SMAD2 complexes in the nucleus of BMPR2-deficient cells (Fig 2E and S3F Fig). Together, this suggests that under BMPR2 deficiency, formation of mixed heteromers comprising BMP type-1 and TGFβ type-1 and type-2 receptors is facilitated. This translates to the formation of mixed SMAD heteromeric complexes indicative of increased lateral responses to TGFβ in BMPR2-deficient ECs.

### BMPR2 deficiency alters expression of mechano-relevant genes

**Fig 3:**
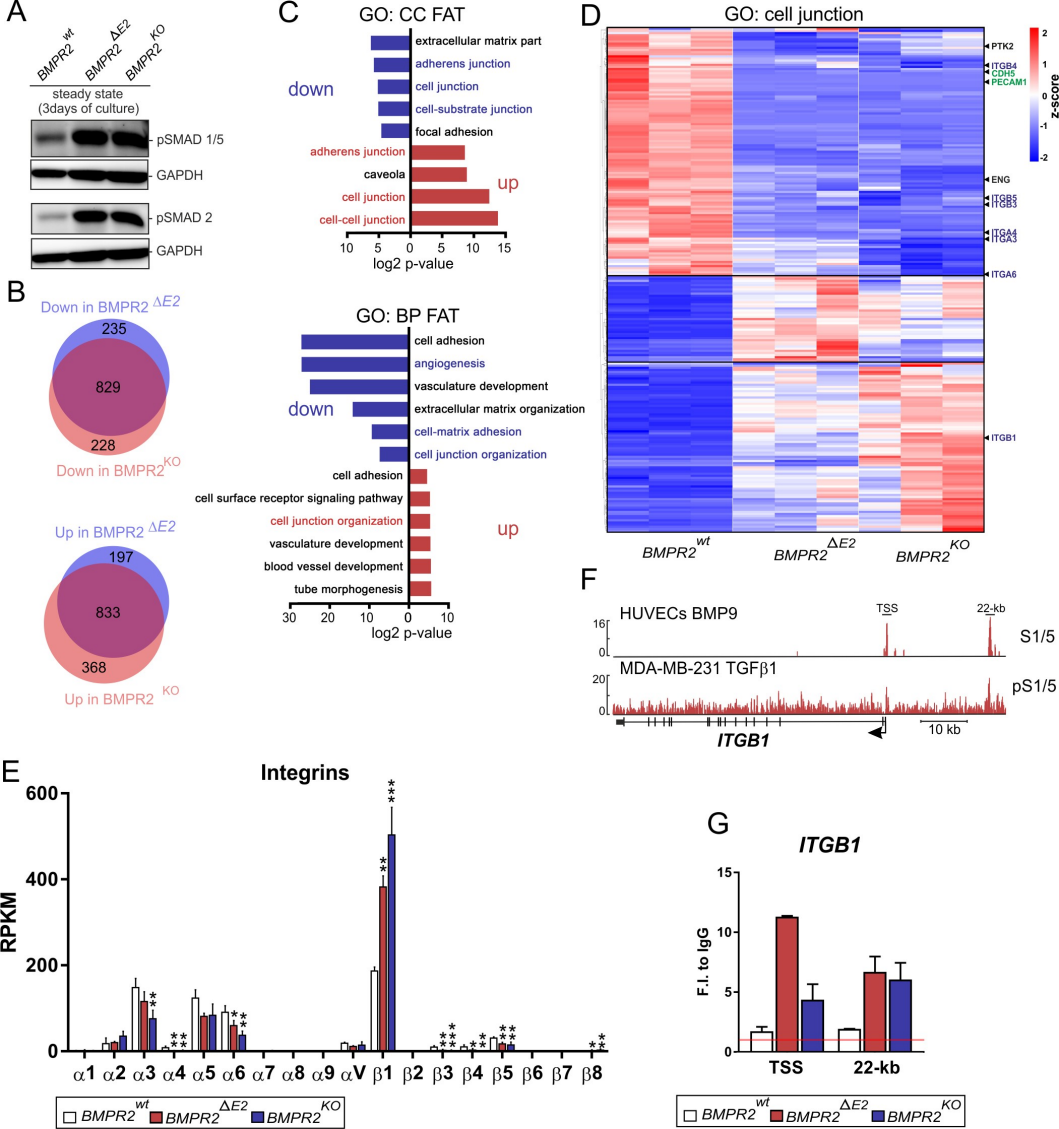
BMPR2 deficiency alters integrin expression. (A) Immunoblot using antibodies specific to pSMAD1/5 or pSMAD2 showing elevated pSMAD levels in indicated cell types cultivated in confluent monolayers for 3 days under steady-state conditions. (B–E) RNA-Seq analysis of WT and BMPR2-deficient ECs under steady-state conditions (*n* = 3 independent replicates). (B) Number of genes significantly differentially regulated in BMPR2-deficient ECs in comparison to WT ECs and their relative proportion. The majority of genes are similarly altered in both BMPR2-deficient cells lines (829 = down; 833 = up). (C) Significantly enriched GO terms of shared deregulated genes. The fold enrichment of up-regulated (red bars) and down-regulated (blue bars) GO terms is shown. Notably, GO terms associated with cell-to-cell and cell-to-substrate connectivity were both up- and down-regulated, suggesting an alteration of the cellular mechanics in absence of BMPR2. (D) Hierarchical clustering of differentially expressed genes associated with the GO term “cell junction” (GO: 0030054). Heatmap color coding shows z-score of differentially regulated genes (red = high; blue = low). (E) Relative expression of integrins under steady-state conditions shown with RPKM values. Note ITGB1 is significantly elevated and the most abundant integrin in BMPR2-deficient ECs. Statistical significance relative to BMPR2^wt^ was calculated using one-way ANOVA and Bonferroni post hoc test. *P < 0.05, **P < 0.01, ***P < 0.001. (F) IGV browser displays over the *ITGB1* loci showing SMAD1/5 ChIP-Seq track of HUVECs treated with BMP9 [53] and pSMAD1/5 ChIP-Seq track of MDA-MB-231 cells treated with TGFβ1 [41]. ChIP-Seq data were retrieved from GEO (GSM684747, GSM2429820). (G) SMAD1 occupancy at the *ITGB1* TSS or the 22-kb regions were validated by ChIP-qPCR in steady-state conditions. IPs are a representative experiment of two and ChIP-qPCR was performed in triplicates shown with mean + SD. See also S4 Fig and S3 Data for underlying data. BMP, bone morphogenetic protein; BMPR2, BMP type-2 receptor; BP, biological process; CC, cellular compartment; ChIP, chromatin immunoprecipitation; EC, endothelial cell; GEO, Gene Expression Omnibus; GO, Gene Ontology; HUVEC, human umbilical vein endothelial cell; IgG, immunoglobulin G; IP, immunoprecipitation; pSMAD, phosphorylated SMAD; qPCR, quantitative PCR; RNA-Seq, RNA sequencing; RPKM, Reads per kilobase per million mapped reads; TGFβ, transforming growth factor-beta; TSS, transcription start site; WT, wild-type; CC FAT, cellular compartment; BP FAT, biological process.

Up to this point, our experiments to characterize SMAD signaling of BMPR2-deficient ECs included starvation of cells followed by stimulation with recombinant ligands. This helped us to understand the altered responsiveness of BMPR2-deficient ECs to BMPs and TGFβ and to decipher receptor and SMAD complexes involved. However, in the quiescent vasculature, ligands such as BMPs and TGFβ are not expected to appear with sharp-arising expression profiles, but their availability is, rather, defined by the cell’s microenvironment, such as serum levels and sequestration of growth factors from the extracellular space. PAH lesions of the pulmonary artery are reminiscent of niche-like microenvironments in which accumulation of local factors is favored [23]. To gain more insights into the cellular long-term adaptation mechanism in response to BMPR2 deficiency, we approached this accumulative micro-milieu character simplistically by analyzing confluent EC monolayers, left untreated for 3 consecutive days in basal growth medium. Under these steady-state conditions, cells adapt to their local environment, and we found pSMAD1/5 and pSMAD2 levels to be increased under BMPR2 deficiency cells (Fig 3A).

To better understand this cellular adaptation in response to loss of BMPR2, we identified differentially regulated genes on a global scale. RNA sequencing (RNA-Seq) revealed that 1,662 genes were found to be differentially regulated in both BMPR2 mutants when compared to WT ECs (Fig 3B). Additional to BMPR2, both ALK1 and co-receptor ENG were prominently down-regulated in the mutant cells (S4A and S4B Fig). Among the ligands, *TGFB1* was highly expressed in comparison to other TGFβs (2/3) and BMPs (2, 4, 6, 7) in all three cell lines, while *BMP2* expression was significantly elevated and *BMP6* expression reduced in the absence of BMPR2 (S4B Fig). Whereas *ACVR1* (ALK2), *ACVR1B* (ALK4), and *TGFBR1* (ALK5) were unchanged or slightly elevated, *BMPR1A* (ALK3) was strongly elevated and *BMPR1B* (ALK6) and *ACVR1C* (ALK7) were barely detected in BMPR2-deficient ECs (S4B Fig). Both *ACTR2A* and *ACTR2B* were significantly down-regulated, and *TGFBR2* was highly expressed (S4B Fig). Subsequent Gene Ontology (GO) analysis revealed enrichment of differentially regulated genes associated with cell-to-cell and cell-to-substrate interfaces (Fig 3C). Hierarchical cluster analysis of genes associated to the GO term “cell-junction” revealed down-regulation of classical EC markers with known function in EC connectivity (*PECAM1*, *CDH5* [vascular endothelial (VE)-Cadherin]) and genes involved in EC substrate adhesion such as *PTK2* (FAK) as well as several integrins (e.g., *ITGB5* and *ITGA6*) (Fig 3D). Together, this suggests a PAH-like receptor signature (BMPR2 down, ALK1 down, ENG down) and, at the same time, altered cell-cell and cell-interface capacity of BMPR2-deficient cells, indicative of strong changes of their mechanical properties, presumably at cell junctions. α/β-integrin subunit oligomerization confers integrin-ECM specificity [54]. While the α1/β1-integrin receptor binds to collagens, α5/β1-integrin binds fibronectin (FN). Activated β subunits associate with the effector protein integrin-linked kinase (ILK), which facilitates association of focal adhesions (FAs) to the contractile actomyosin cytoskeleton supporting integrin-mediated traction forces [55]. Surprisingly, direct comparison of integrin α/β subunit expression showed that *ITGB1* was the predominant integrin and significantly elevated in BMPR2-deficient ECs (Fig 3E), which we additionally validated by quantitative real-time PCR (qRT-PCR) (S4C Fig). TGFβ was shown before to regulate ITGB1 levels associated to a profibrotic/injury-like phenotype in different models [56–58]. Analysis of publicly available SMAD1/5 chromatin immunoprecipitation sequencing (ChIP-Seq) data from human umbilical vein endothelial cells (HUVECs) treated with BMP9 [53] indicated SMAD1/5 occupancy in close proximity to the ITGB1 transcription start site (TSS) and at a 22 kb upstream region (22k). In epithelial cells treated with TGFβ, pSMAD1/5 similarly occupied the 22-kb upstream region of ITGB1 (Fig 3F) [41]. In order to prove whether in BMPR2-deficient cells increased *ITGB1* expression is a function of SMAD1 binding, we performed SMAD1-ChIP followed by quantitative PCR (qPCR) under steady-state conditions. ChIP experiments revealed stronger SMAD1 occupancy at the *ITGB1* TSS and the 22-kb upstream region when BMPR2-deficient cells adapted to their microenvironment (Fig 3G). A similarly increased SMAD1 occupancy at the *ID3* promotor was found following the same experimental strategy (S4D–S4F Fig).

### Mechano-complexes comprising β1-integrin and ILK localize at cell junctions of BMPR2-deficient ECs

**Fig 4:**
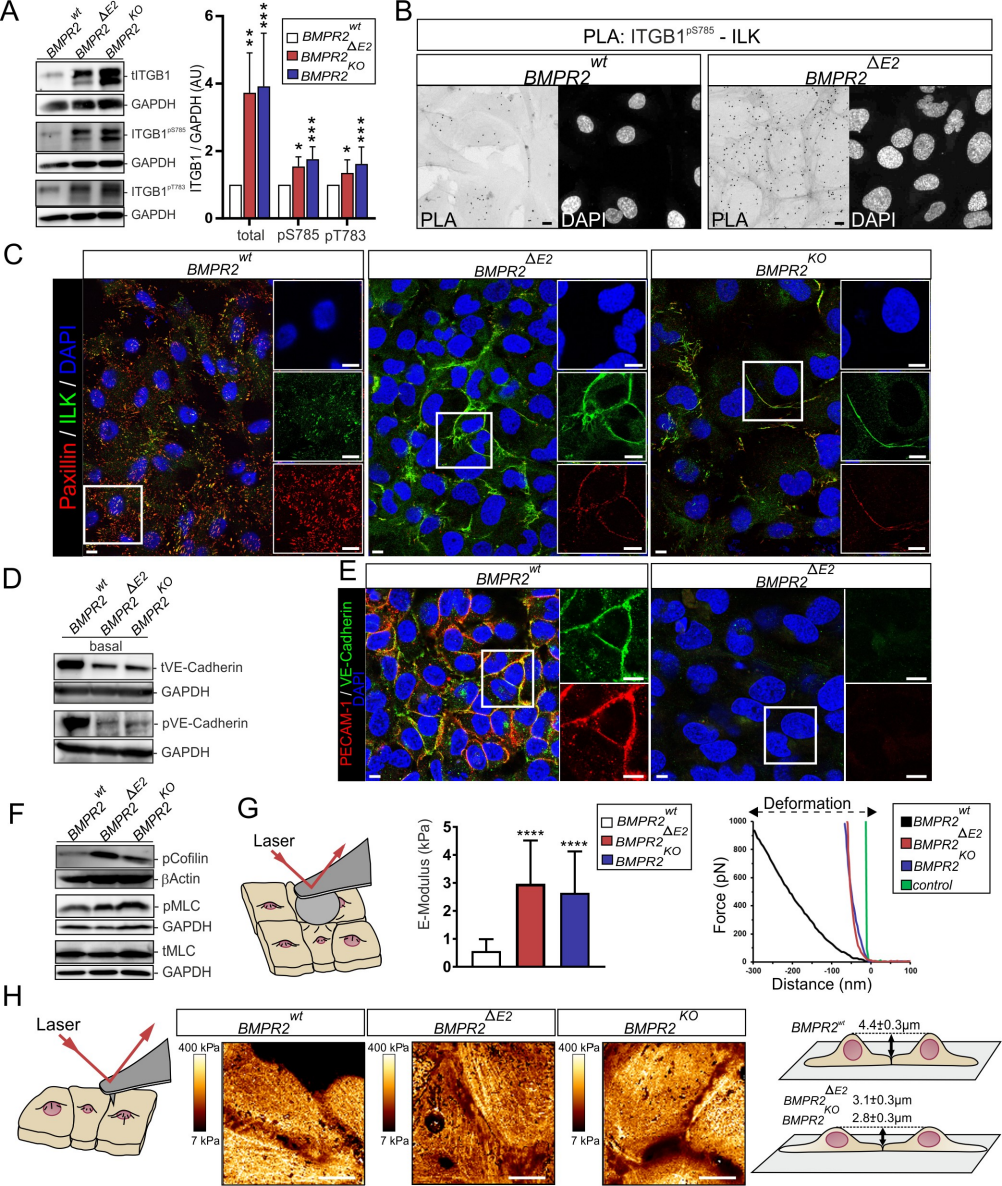
BMPR2-deficient ECs show increased activation of β1-integrin-ILK mechano-complexes, re-localization of these complexes to cell junctions, and junctional stiffening. (A) Immunoblot showing total β1-integrin (tITGB1) levels and phosphorylation at Ser785 and Threonine 783 (left) under steady-state conditions and densitometric quantification of total or phosphorylated β1-integrin levels in indicated cell clones (right). Data are presented as mean + SD (*n* = 3–4 independent experiments). (B) PLA between active β1-integrin (ITGB1^pS785^) and ILK under steady-state growth conditions. (C) Single confocal z-planes of BMPR2^wt^ (basal, left), BMPR2^ΔE2^ (medial, middle), and BMPR2^KO^ (medial, right) depicting the localization of ILK (green) or paxillin (red). Insets show zoomed-in regions. (D) Immunoblots showing protein levels of VE-Cadherin and phosphorylated VE-Cadherin in indicated ECs under steady-state conditions. (E) Single confocal z-planes of immunocytochemical staining using antibodies specific for VE-Cadherin (green) and PECAM-1 (red). Insets show zoomed-in regions. (F) Immunoblots showing levels of phosphorylated cofilin (pSer-3), pMLC (pSer-19), and total MLC in indicated ECs under steady-state culture conditions. (G) Cartoon (left) depicting the principle of CFS (particle diameter 23 μm). Elastic modulus derived from CFS of living cells (middle). Representative indentation (force versus distance) curves for the different cell lines and the hard control surface (mica; right). Data are shown as mean + SD (*n* = 30). (H) Principle of QI using a sharp cantilever tip (diameter < 20 nm; left). Representative QI scans of fixed cells, focusing on CCC sites (middle). Height profiles of nucleus-to-cell junction height differences (black arrows) are indicated. Given values are expressed as the mean ± SD. Height profile measurements were taken from *n* ≥ 18 cells; scale bars, 10 μm; Statistical significance relative to BMPR2^wt^ was calculated using Kruskal-Wallis test with post hoc Dunn test. *P < 0.05, **P < 0.01, ***P < 0.001, ****P < 0.0001. See also S5 Fig and S4 Data for underlying data. BMP, bone morphogenetic protein; BMPR2, BMP type-2 receptor; CCC, cell-to-cell contact; CFS, colloidal force spectroscopy; EC, endothelial cell; ILK, integrin-linked kinase; MLC, myosin light chain; PECAM-1, platelet endothelial cell adhesion molecule-1; PLA, proximity ligation assay; pMLC, phosphorylated MLC; QI, quantitative imaging; pS785, phosphorylated Serine 785; pT783, phosphorylated Threonine 783; tITGB1, total integrin beta-1; VE, vascular endothelial.

Next, transcriptional regulation of ITGB1 was validated on protein level. BMPR2-deficient ECs showed strong up-regulation of β1-integrin protein and its activation, indicated by phosphorylation at Tyrosine 783 (Tyr783) and Serine 785 (Ser785) (Fig 4A) [59,60].

PLA using antibodies against the C-terminal phosphorylated site of β1-integrin and ILK confirmed their enhanced complex formation in BMPR2-deficient cells indicating mechanically active β1-integrin mechano-complexes (Fig 4B). The presence of paxillin is necessary for targeting ILK to FAs [61]. Both paxillin and ILK strikingly changed their subcellular localization in BMPR2-deficient ECs from discrete basal FA foci (WT cells) to long filamentous-like structures residing in cell junctions (Fig 4C). These structures resemble adherens junctions (AJs). The AJ proteins VE-Cadherin and PECAM-1 [62] are both down-regulated and largely absent at cell junctions of BMPR2-deficient ECs (Figs 3D, 4D and 4E). Together, this highlights the presence of β1-integrin-ILK mechano-complexes in cell junctions and strong remodeling of AJs in BMPR2-deficient ECs.

### EndMT and alterations in F-actin organization induce subcellular stiffening

The loss of VE-Cadherin and PECAM-1 strongly pointed toward EndMT, a process shown to contribute to EC dysfunction [63]. However, the biomechanical aspects of EndMT remain elusive. TGFβ signals induce epithelial-to-mesenchymal transition (EMT), following similar basic principles to EndMT, including a switch in cadherins [64,65]. Here we show that BMPR2-deficient ECs switch from VE-Cadherin to junctional N-Cadherin (Fig 4E and S5A Fig). Consequently, the pan-Cadherin effector protein β-catenin remains at EC junctions of BMPR2-deficient cells, although with a less discrete pattern (S5B Fig). Similarly, changes in cell junction architecture were indicated by the GO analysis (Fig 3C). The switch of Cadherins and remodeling of EC junctions also suggests junction breakdown. This is underlined by morphological changes of the cell-membrane architecture with unorganized long-cell protrusions at cell contacts of BMPR2-deficient ECs, while junctions of WT ECs appear confined (S5C Fig). Finally, EndMT markers *SLUG* and *TWIST* are up-regulated in BMPR2-deficient cells (S5D Fig). Transition to mesenchymal cells with myo-fibroblastic character would lead to increased ECM deposition and increased cellular traction forces and contractility. To relate the morphological changes to contractile cell mechanics, we characterized regulation of F-actin assembly and myosin activation. We found strong phosphorylation of cofilin as well as myosin light chain (MLC), suggesting reduced F-actin severing and increased actomyosin contractility (Fig 4F and S5E Fig). Phalloidin staining revealed an increase in stress fibers at the basal side of BMPR2-deficient cells, while apical F-actin stress fibers adopt two discrete orientations at cell junctions: filopodia-like protrusions bridging perpendicular to cell junctions and thicker bundles of F-actin spanning the cell cortex adopting a parallel junctional orientation (S1 Movie and S5F Fig). Stiffening of cells and their extracellular environment is one mechanical aspect reported as a hallmark for some vascular disorders [22,66,67]. We therefore investigated cellular stiffness of living cells by colloidal force spectroscopy (CFS). A 4-fold increase of the Young’s modulus in BMPR2-deficient ECs indicates gain in cell stiffness (Fig 4G), in line with the observed increase in apical F-actin stress fibers. To depict these stress fibers with higher resolution, we employed atomic force microscopy (AFM)-based quantitative imaging (QI) of fixed cells, revealing discrete F-actin bundles of high relative stiffness, which align parallel to junctions. Moreover, BMPR2-deficient cells display a reduced height profile (Fig 4H). Together, BMPR2-deficient ECs undergo EndMT and subcellular stiffening through F-actin bundling and actomyosin contraction, initiated at lateral EC contact sites. This together suggests a conversion of the ECs toward a contractile myo-fibroblastic phenotype.

### BMPR2-deficient ECs display altered matrisome profile and spread on and remodel ECM via β1-integrin and Rho-associated kinase (ROCK) signaling

**Fig 5:**
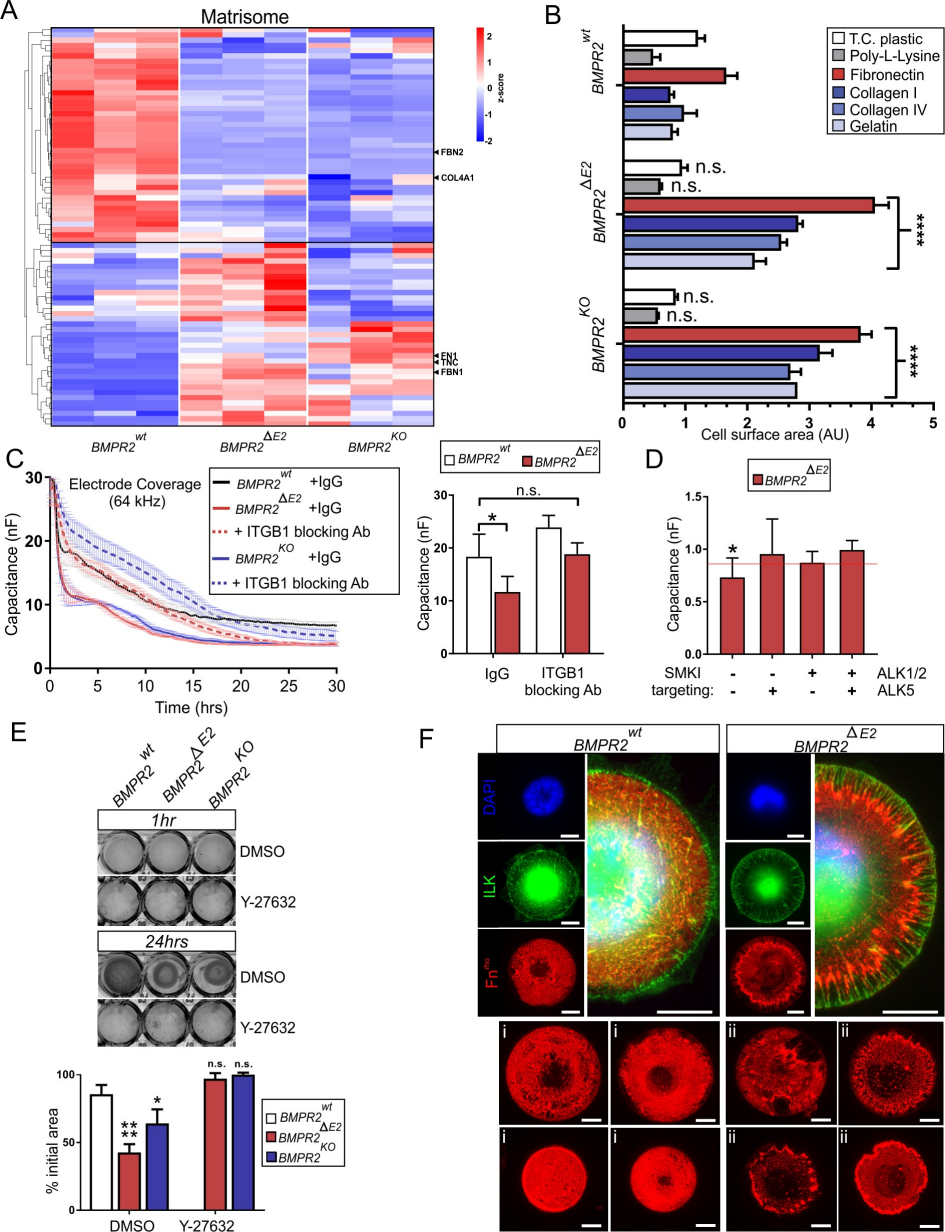
BMPR2-deficient ECs display altered ECM expression profile, spread on and remodel RGD-containing ECM, and exert high traction forces dependent on β1-integrin activity. (A) RNA-Seq analysis of WT and BMPR2-deficient ECs under steady-state conditions (*n* = 3 independent replicates). Hierarchical clustering of differentially expressed genes associated with the GSEA gene set “NABA_CORE_MATRISOME” (M5884). Heatmap color coding shows z-score of differentially regulated genes (red = high; blue = low). (B) Cell adhesion on dishes (TC plastic) coated with ECM proteins (all 5 μg/cm^2^). Spreading area is expressed as AUs. Data are presented as mean + SD (two-way ANOVA with post hoc Bonferroni relative to *BMPR2*^*wt*^, *n* = 3 independent experiments). See also S6 Fig for representative images of cells. (C) ECIS measurement at 64 kHz over 30 h monitoring cell adhesion and spreading on gelatin-coated surfaces showing decay of mean capacitance (nF) ± SEM for WT and BMPR2-deficient cells upon seeding in basal growth media and when cells were additionally pre-incubated with nonspecific antibody isotype control (IgG) or β1-integrin blocking antibody against the ecto-domain (slash-dotted line). Two-hour time point is depicted as mean capacitance ± SD (right), showing that interference with β1-integrin function by blocking antibodies resembles IgG control for WT cells; two-way ANOVA with post hoc Bonferroni relative to IgG *BMPR2*^*wt*^ was performed for *n* = 2–4 independent experiments. (D) ECIS adhesion assay upon 3 d SMKI treatment targeting ALK5 or ALK1/2 with SB-431542 and K02288. Plot shows mean capacitance (nF) ± SD, 2 h after seeding normalized to 0 h. Red line represents mean capacitance of DMSO-treated *BMPR2*^*wt*^ cells. Note that BMPR2-deficient cells have lost their increased adhesion and spreading potential compared to *BMPR2*^*wt*^ when cells were subjected to ALK5, ALK1/2, and double SMKI treatment (*n* ≥ 3 independent experiments). Statistical significance was calculated between DMSO-treated WT and BMPR2-deficient cells using an unpaired two-tailed Student *t* test. (E) Collagen lattice contractility assay showing contraction of collagen I lattice co-cultured with indicated EC clones in the absence (DMSO) or presence of ROCK inhibitor Y-27632 (10 μM) (upper). Quantification of lattice diameter constriction (as compared to initial diameter at 1 h) expressed as percent of initial diameter after 24 h of incubation (lower). Data are presented as mean + SD (two-way ANOVA with post hoc Bonferroni relative to DMSO *BMPR2*^*wt*^, *n* = 3 independent experiments). (F) Immunocytochemical staining of indicated single cells growing on FN-coated circular micropatterns for 24 h showing the relative localization of ILK (green) and remodeled FN (red) (upper). Representative epifluorescence images for different diameter circular micropatterns cultured with single *BMPR2*^*wt*^ (i) or *BMPR2*^*ΔE2*^ (ii) cells, depicting underlying FN (red) coating after 24 h (lower). Scale bars, 10 μm. *P < 0.05, **P < 0.01, ***P < 0.001, ****P < 0.0001. See also S6 Fig and S5 Data for underlying data. ALK5, activin receptor-like kinase 5; AU, arbitrary unit; BMP, bone morphogenetic protein; BMPR2, BMP type-2 receptor; EC, endothelial cell; ECIS, electric cell-substrate impedance sensing; ECM, extracellular matrix; FN, fibronectin; GSEA, Gene Set Enrichment Analysis; IgG, immunoglobulin G; ILK, integrin-linked kinase; n.s., not significant; RNA-Seq, RNA sequencing; ROCK, Rho-associated kinase; SMKI, small-molecule kinase inhibitor; TC, tissue culture; WT, wild type.

Basal cell-to-ECM contacts (CMCs) and junctional cell-to-cell contacts (CCCs) are linked to the actomyosin cytoskeleton, providing traction forces via integrins and cadherins, respectively [68]. β1-subunit–containing integrin complexes bind to FN1 and tenascin-C (TNC) via the RGD peptide sequence. Both *FN1* and *TNC* were up-regulated in BMPR2-deficient cells, whereas the EC basal-membrane–related *COL4A1* was down-regulated (Fig 5A).

Secretion of FN1 and increase in contractility is associated with a myo-fibroblast phenotype [69], while TNC expression was shown before to be up-regulated in BMPR2-deficient smooth muscle cells (SMCs) [70]. Of note, COL1-rich ECM was described in advanced PAH lesions [22,67,71], while COL4 was shown to be down-regulated in BMPR2 dysfunctional ECs from PAH donors [72]. Together, we found that, when BMPR2-deficient ECs adapted to their micro-milieu, they displayed an ECM signature indicative of EC dysfunction and reminiscent of reported changes in the ECM expression profile of PAH lesions. We set out further to connect β1-integrin up-regulation to this altered ECM expression profile and found it interesting that the majority of up-regulated ECM proteins harbor RGD motives. FN fibers act as extracellular scaffolds for fibrillin-1 (FBN1) microfibril assembly [73], with FBN microfibrils being the major ECM to bind the latency-associated peptide (LAP) form of TGFβ. Our results show that BMPR2-deficient cells indeed interact strongly with different RGD-containing ECM proteins, namely FN1, COL1, COL4, or gelatin. Increased cell adhesion and spreading was confirmed by approximately 2.5-fold increase in spreading area (Fig 5B and S6 Fig). This was not the case for cells seeded on tissue culture (TC) plastic or poly-L-lysine coatings, underlining strong changes in cell-matrix interactions upon BMPR2 deficiency. Adhesion and spreading dynamics were followed by real-time impedance measurements to prove that β1-integrin is indeed responsible for increased ECM interactions. While BMPR2-deficient cells showed enhanced adherence and spreading compared to WT cells, addition of blocking antibodies against β1-integrin converges their capacitance kinetics to WT properties (Fig 5C). By pretreatment of cells with SMKIs targeting either ALK5 (SB-431542) and/or ALK1/2 (K02288) under steady state, we aimed to show the contribution of these type-1 receptors in the increased adhesion and spreading phenotype of BMPR2-deficient cells. For this, we treated cells with SMKIs for 3 consecutive days before we subjected them to the electric cell-substrate impedance sensing (ECIS) measurement. After 2 h of cell seeding, we again found increased adhesion and spreading of BMPR2-deficient cells (Fig 5D, left), whereas blocking ALK5, ALK1/2, and a combination of both efficiently rescued these effects of BMPR2-deficient cells compared to DMSO-treated controls (Fig 5D). To test whether the conversion toward a myo-fibroblast–like phenotype is accompanied by increased contractility, we performed a COL1 lattice assay [74] (Fig 5E). Because increased non-muscle myosin motor activity was indicated (Fig 4F and S5E Fig), we also targeted the MLC upstream kinase ROCK. BMPR2-deficient cells efficiently contracted the COL1 lattice. In contrast, inhibiting ROCK by Y-27632 blocked this response (Fig 5E). Taken together, increased β1-integrin expression in BMPR2-deficient cells increases their interaction with RGD-containing ECM. This is dependent on ALK5, ALK1/2, and their ROCK-MLC–mediated contractility.

### BMPR2-deficient ECs exert increased matrix remodeling particularly at cell junctions

**Fig 6:**
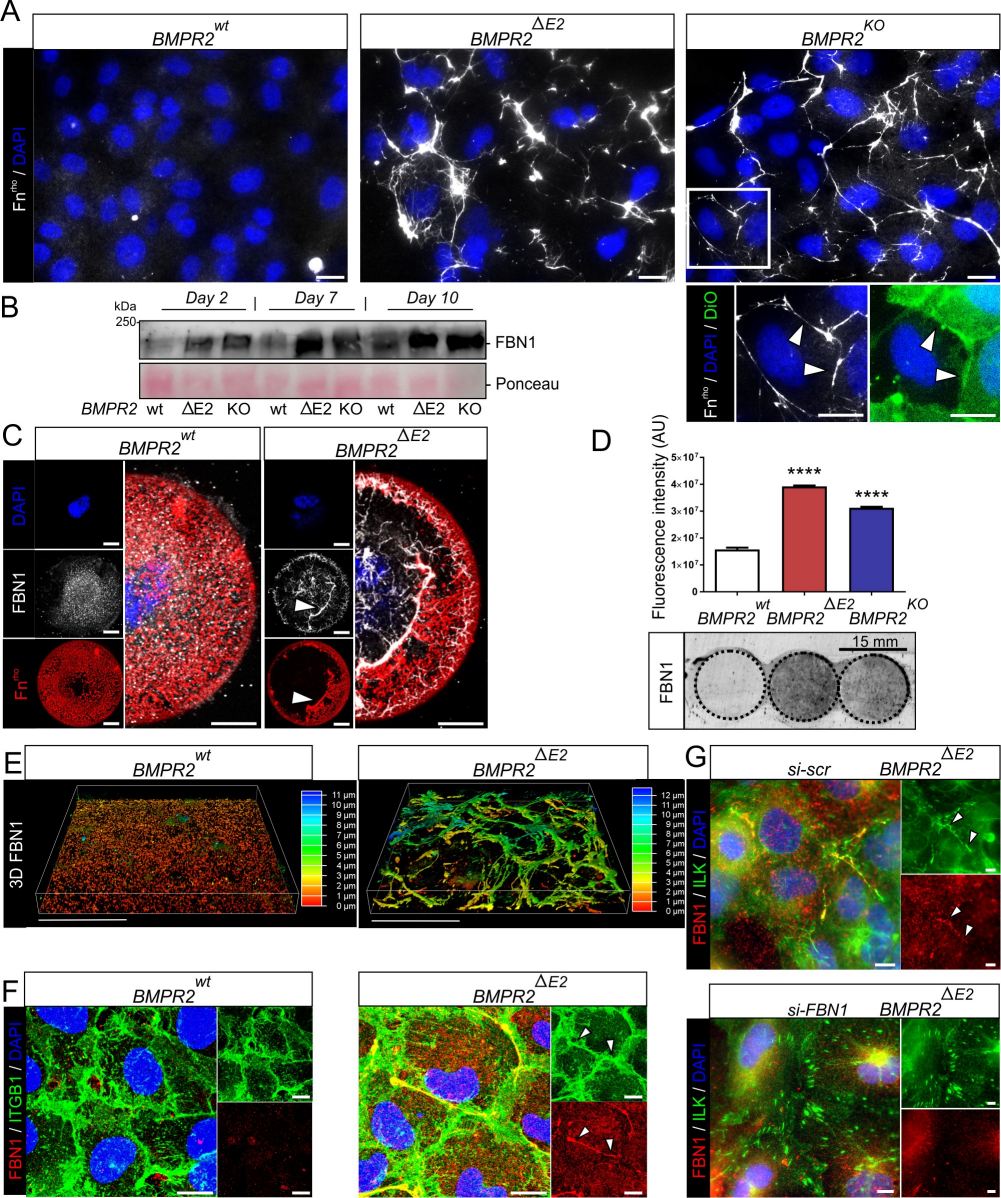
BMPR2-deficient ECs remodel Fibronectin and Fibrillin particularly at β1-integrin-ILK–rich cell junctions. (A) Epifluorescence images showing indicated cell clones cultured for 3 d in the presence of FN^rho^ (20 μg/ml) (white, pseudo-color) and counterstained for cell membrane (DiO, green). Figure enlargements are indicated by white frame (lower). Enlargement showing relative localization of cell boundaries (green) and FN fibers (white) indicated by white arrowhead. Scale bar, 20 μm. (B) Immunoblot using an antibody against FBN1 on conditioned supernatants of indicated cell clones (upper) together with Ponceau-S staining (lower). Cells were cultured under steady-state growth conditions, and equal volumes of supernatants were harvested after 2, 7, and 10 d and concentrated. (C) Epifluorescence pictures/images of indicated cell clones seeded on circular micropatterns coated with FN (red). Immunocytochemical staining shows FBN1 (white pseudo-color) after 24 h of culture deposited on FN-coated micropatterns. Sites of active co-remodeling are indicated by white arrowheads. (D) Quantification of FBN deposits on 15 mm coverslips after decellularization (upper) using a fluorescence laser scanner (Cy3 emission). The quantified coverslip area is indicated by slash-dotted circle (lower). Data are presented as mean + SD (one-way ANOVA with post hoc Bonferroni relative to *BMPR2*^*wt*^, *n* = 3). (E) Re-constructed confocal z-stacks (volume rendering) of FBN1 immunostaining depicted by topographical color-coding, indicating relative z-position of fluorescent signal (approximately 0–4 μm red-yellow/basal; approximately 4–7 μm green/medial; approximately 8–11 μm blue/apical). Note the large number of medially located signals. (F) Maximum projections of confocal z-stacks showing relative localization of β1-integrin (ITGB1, green) and FBN1 (red). Co-localization is indicated by white arrowheads (right). See S7B Fig for side-view projection. (G) Epifluorescence images showing relative ILK (green) and FBN1 (red) localization upon control (*scrambled) si-RNA* transfection (upper) or FBN1 knock-down (lower). Scale bars, 10 μm (panels A, C, F, and G) and 50 μm (panel E). See also S7 Fig and S6 Data for underlying data. BMP, bone morphogenetic protein; BMPR2, BMP type-2 receptor; Cy3, cyanine dye 3; DiO, green fluorescence emission lipophilic carbocyanine dye; EC, endothelial cell; FBN, fibrillin; FN, fibronectin; FN^rho^, rhodamine-labeled FN; ILK, integrin-linked kinase.

COL1 lattice contraction lacks single-cell resolution. We therefore took advantage of poly(dimethylsiloxane) micropatterns with different diameter and functionalized with rhodamine-labelled FN (FN^rho^) to focus on single-cell–based ECM remodeling. Interestingly, while ILK localized at junctions of monolayers in filamentous structures (Fig 4C), in single BMPR2-deficient cells, ILK predominantly localized in filamentous foci at their periphery (Fig 5F). Remarkably, at these ILK-rich cell edges, we noticed heavy FN remodeling suggesting strong traction forces, which were independent of pattern diameter and cell polarity (Fig 5Fii and 5Fiii). To prove that increased FN remodeling is also a feature of BMPR2-deficient monolayers, we incubated living cells with FN^rho^ added in solution. While WT ECs undergo vesicular FN uptake, BMPR2-deficient cells remodel exogenous FN fibrils within the extracellular space particularly at CCC sites (Fig 6A and S2 Movie).

Thus, increased actomyosin-dependent (con-) traction forces reveal that BMPR2-deficient cells strongly engage with and remodel RGD-peptide–containing matrix. In our model, FBN1 appears as an ECM protein connected to BMPR2 deficiency, and its up-regulation together with FN (Fig 5A) may—together with the previously mentioned mechanism—be the missing link to understand the gain in TGFβ signaling. FN deposition and remodeling often precede FBN1 microfibril assembly [73,75]. FBN1 itself binds α5/β1-integrin [76]. More importantly, release of mature TGFβ from FBN1-bound LAP-TGFβ requires mechanical pulling of integrin complexes at the RGD site of LAP [77–80]. First, we found that conditioned supernatants of BMPR2-deficient cells are indeed enriched in FBN1 (Fig 6B), while FBN1 fibers partially co-localized with remodeled FN on single-cell micropatterns (Fig 6C, white arrows). More remarkably, we found a densely assembled FBN1 fiber network after longer steady-state cultivation (6 d) of confluent BMPR2-deficient ECs (Fig 6D and 6E and S7E Fig), underlining that ECM production of BMPR2-deficient ECs is a function of their long-term adaptation to their micro-milieu. While smaller FBN1 deposits were already found at the basal side of WT ECs, BMPR2-deficient cells show increased FBN1 fibrillogenesis, giving rise to thick, cable-like fibers with random orientations in the x, y, and atypically also the z-plane, spanning the basal to apical side (Fig 6E and S3 Movie). These FBN1 fibers partially adopt the same junctional orientation as peripheral ILK (Fig 6E and S7A Fig and S4 Movie,) and β1-integrin (Fig 6F and S7B Fig,) at CCC-sites. FN, FBN1, β1-integrin, and ILK co-localization and functional dependency for cell mechanics is further highlighted by the co-alignment of intracellular F-actin cables with endogenous FN and FBN1 fibers (S7C and S7D Fig). Moreover, we could efficiently rescue the localization of ILK back to CMC sites by FBN1-RNA interference (Fig 6G). This suggests that the atypical localization of ILK is indeed an adaptation to ectopic FBN1 deposition. Together, this identifies FBN1 as a key ECM protein, remodelled by BMPR2-deficient ECs and deposited into CCC sites.

### Ectopic FBN1- rich ECM is also found in PAs from IPAH and heritable PAH donors with low BMPR2 expression

**Fig 7:**
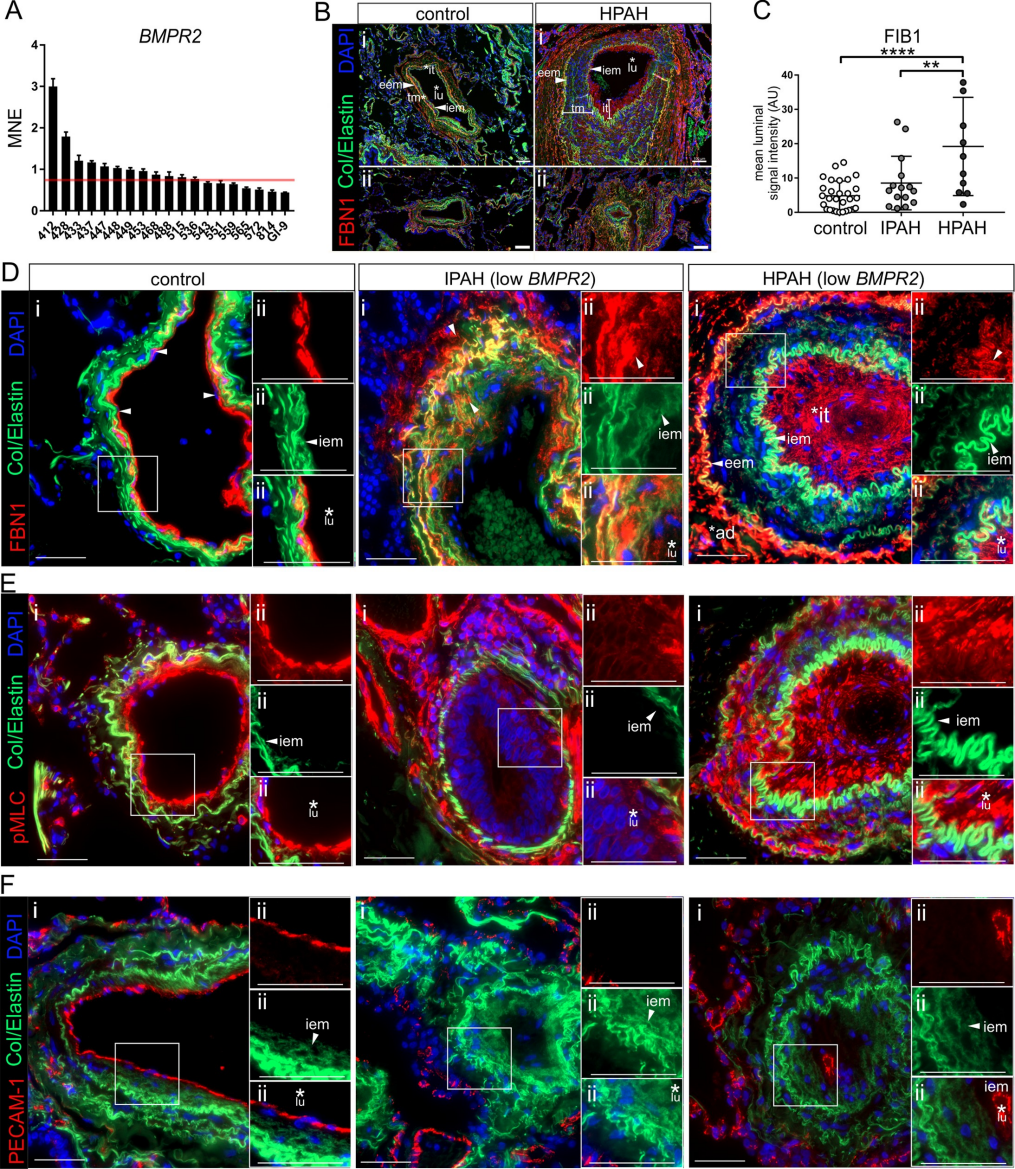
Ectopic FBN1 deposits, actomyosin contractility, and loss of endothelial character found in inner luminal PAs from IPAH and HPAH donors with low BMPR2 expression. (A) qRT-PCRs of whole lung tissue from IPAH and HPAH donors (*n* = 19) analyzed for their BMPR2 transcript levels. Red line indicates mean BMPR2 transcript levels in control tissue (*n* = 9). (B) Immunohistochemical stainings of control and HPAH tissue sections showing large (ii) and small (ii) PAs at the level of the terminal bronchioles; 10-μm-thick sections were stained for FBN1 (red) and DAPI (blue). Autofluorescence of elastic membranes (collagen and elastin) at approximately 520 nm emission (green) was used to identify relative locations of iem and eem. Note hypertrophy of intima and thickened tm in HPAH PAs when compared to control. Ectopic FBN1 deposits are found to exceed the iem toward the lumen and to add up to intimal hypertrophy. Concomitantly, few FBN1 deposits are found in medial, while larger deposits are found in adventitial regions. (C) Quantification of luminal FBN1 deposits in PAs from regions beyond the border indicating the iem; ≥10 different PAs originating from up to 3 different donors were compared (control *n* = 3, IPAH *n* = 3, HPAH *n* = 1). (D) Representative PAs of controls, IPAH, and HPAH donors were stained for FBN1, collagen, and elastin at approximately 520 nm emission (green) and DAPI (blue) (i). Higher magnification of the area surrounding the iem (ii) shows clear restriction of FBN1 to sub-EC layer (e.g., basal lamina) in controls, while FBN1 deposits are also found to extend into the lumen of PAs from IPAH (middle) and HPAH (right) donors. Note the fragmented structure of FBN1 deposits in media of IPAH plexiform lesions. In contrast, low amounts of medial FBN1 deposits versus pronounced eem, adventitial and intimal localization is found in HPAH PAs (asterisks, arrowheads). (E) Sections were stained for pMLC (red) indicating actomyosin-dependent contractility. In controls, pMLC signals are found confined in close proximity to the iem, whereas in both IPAH and HPAH, pMLC is also found exceeding the iem toward the luminal side. (F) PECAM-1 staining (red) was used to show localization of ECs in PAs from controls and IPAH/HPAH. In controls, ECs locate with distinct inner luminal distribution and connectivity in close proximity to the iem. In plexiform lesions of IPAH and HPAH, few PECAM-1–positive cells could be identified in far distance to the iem indicating loss of endothelial character. Scale bar represents 100 μM (panel B) or 50 μm (panels D–F). See also S8 Fig and S7 Data for underlying data. ad, adventitial; BMP, bone morphogenetic protein; BMPR2, BMP type-2 receptor; EC, endothelial cell; eem, external elastic membrane; FBN1, fibrillin-1; HPAH, heritable PAH; iem, internal elastic membrane; IPAH, idiopathic PAH; it, intima; lu, lumen; PA, pulmonary artery; PECAM-1, platelet endothelial cell adhesion molecule-1; pMLC, phosphorylated myosin light chain; qRT-PCR, quantitative real-time PCR; tm tunica media.

To test whether our findings in BMPR2-deficient cells regarding the deposition of ectopic FBN1 have the same relevance in human tissues, we next analyzed BMPR2 expression in lung samples from patients with documented forms of IPAH or familiar PAH (heritable PAH [HPAH]) (Fig 7A).

Further analysis was performed with samples of donors, which displayed BMPR2 expression levels below control samples. Characterization of histological sections of PAs from HPAH donors exhibited characteristic hallmarks of PAH lesions in the pulmonary artery at the level of the terminal bronchioles. These include intimal (“it” in Fig 7) thickening as well as thickening of the *tunica media* (tm), which is also indicated by the increased distance between the inner (IEM) and external (EEM) elastic membranes (Fig 7B). Quantification of luminal FBN1 deposits in PAs from low–BMPR2-expressing IPAH and HPAH donors are shown in Fig 7C. In line with our BMPR2- deficient EC model, we find significant increase in ectopic FBN1 deposition in HPAH PAs, whereas closer histological analysis revealed also ectopic FBN1 deposits—although differently organized—in IPAH lesions (Fig 7D). Here, the structure and localization of FBN1 deposits appears more transient. The remodeling of PAH lesions is a consequence of cellular transition, proliferation, apoptosis, cell migration, and cell mechanics, and ECM production and degradation thereof. Pulmonary artery pathology can be classified according to Simonneau and Tuder with a remarkable heterogeneity of PAH lesion severity reflecting the disease state [81,82]. As part of this heterogeneity, we also found the structure and location of FBN1 deposits to be nonuniform. In control PAs, FBN1 localized with a discrete pattern in close proximity to elastic membranes (Fig 7B and 7D, left, arrowheads). Plexiform lesions typically appear in the setting of collagen vascular diseases [24], and resolution of the discrete elastic membrane structures adds up to their advanced remodeling state. In grade IV plexiform lesions (degraded IEM and EEM appearance), FBN1 appears fragmented (Fig 7D, middle). In contrast, grade III lesions from HPAH donors still contain more intact FBN1 deposits with pronounced intimal thickening, lumen occlusion, and more intact EEM and IEM. Here, areas of ectopic FBN1 deposition co-localized strongly with cells that aid the lumen occlusion process (Fig 7D, right). At the same time, the level of FBN1 deposits in medial regions was relatively low.

Latent transforming growth factor beta-binding protein 1 (LTBP1) targets latent complexes of TGFβ to the ECM, where the latent cytokine is subsequently activated [83,84]. LTBPs and fibrillins are highly homologous molecules, and co-localization in the matrix of cultured cells has been reported [83]. We therefore assessed whether LTBP1 is detectable at sites of intimal thickening. Indeed, while in controls LTBP1 was restricted to the IEM, in IPAH and HPAH lesions LTBP1 was found to exceed the IEM toward the luminal side in close proximity to cells adding up to intimal thickening (S8A Fig). Relating to the actomyosin contractility, we performed phosphorylated MLC (pMLC) stainings and found that, while in control PAs pMLC staining was confined to the IEM, more pMLC-positive cells were detected in regions of intimal thickening and lumen occlusion of PAs from both IPAH and HPAH (Fig 7E). In support of findings from our BMPR2-deficiency cell model, this could suggest that also in vivo de-differentiated ECs adopted a myo-fibroblastic character, deposit FBN1, and are highly contractile. Finally, to test whether FBN1-depositing cells have endothelial character, we also performed PECAM-1 stainings in PAs from control and PAH donors. While in controls PECAM-1 gives a discrete intimal localization identifying one discrete layer of closely connected ECs lining the lumen, we found no to very little PECAM-1–positive cells in the PAH lesions investigated (Fig 7F). At the same time, alpha smooth muscle actin (αSMA) gave a strong staining in cells that were found to add up to intimal thickening and lumen occlusion, whereas in control PAs, αSMA is restricted to the media below the IEM but not the EC layer (S8B Fig). This suggests that within pulmonary artery lesions, cells that add up to intimal thickening and lumen occlusion deposit FBN1/LTBP1 and are contractile while their EC character is lost. However, the structure and intensity of FBN1 deposits may be dependent on the grade of the PAH lesion investigated, as end-stage grade IV plexiform lesions show different FBN1 localization and less confined structure compared to grade III lesions characteristic of earlier stages of progressive intimal thickening and lumen occlusion.

### Mechano-adaptation of BMPR2-deficient ECs allows for efficient retrieval of LAP-TGFβ

**Fig 8:**
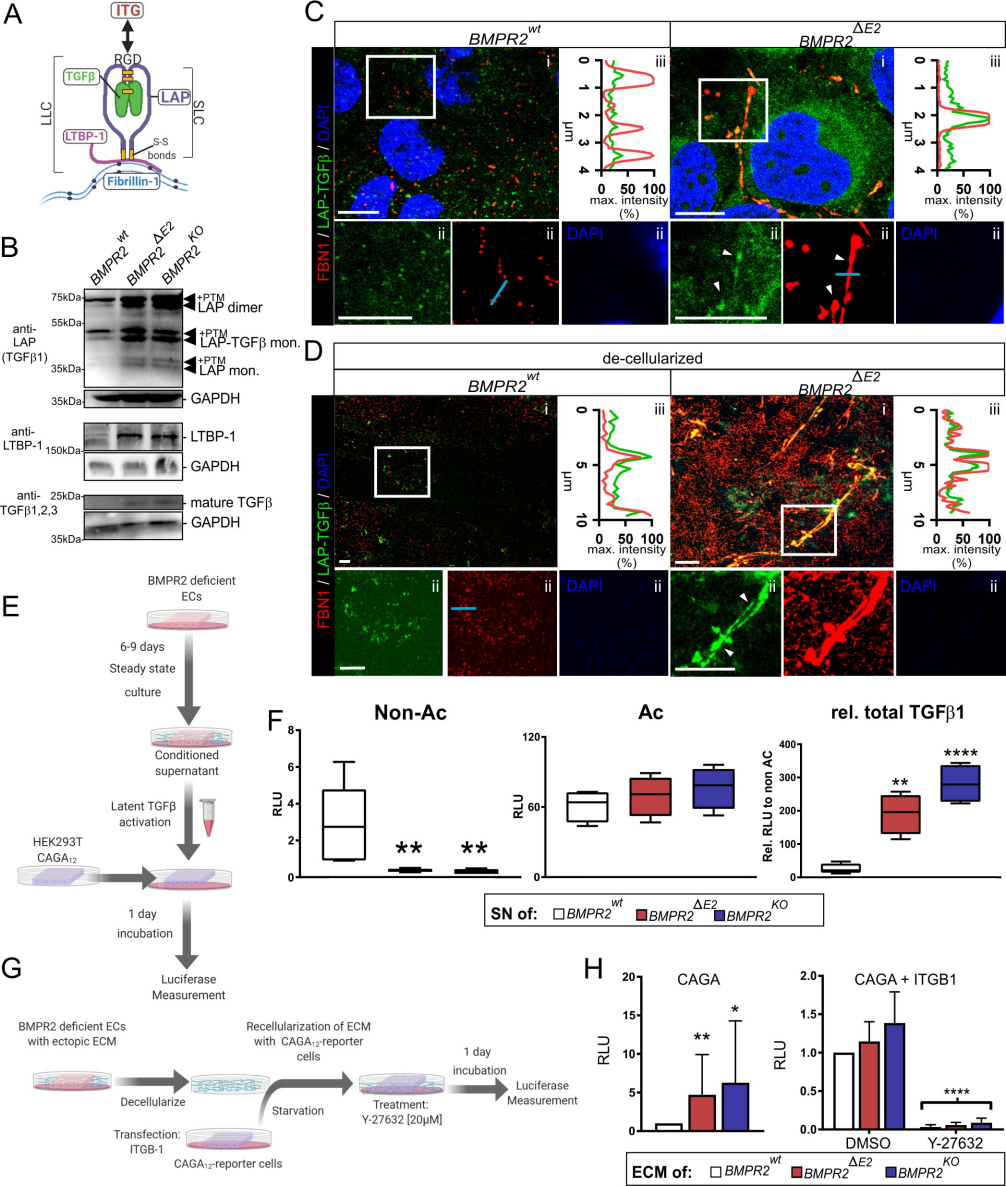
The mechano-adaptation of BMPR2-deficient ECs leads to increased retrieval of TGFβ from extracellular latency depots. (A) Cartoon depicting the structure of the LLC tethering the SLC via LTBP to fibrillin. TGFβ interacts with LAP exposing RGD sequence. Disulfide bonds are depicted in orange. (B) Total cell lysates including cell extract and ECM deposits analyzed using antibodies specific to LAP of TGFβ1 (upper panel) and LTBP-1 (middle panel) as well as mature TGFβ (lower panel). Different variants of LAP complexes are identified under non–fully-reducing conditions including LAP-only monomers (approximately 35 kDa), LAP as part of the monomeric SLC (approximately 50 kDa), and LAP-only dimers (approximately 70 kDa), of which the mature ligand has been cleaved off and retrieved by cells. The upper bands of these individual forms reflect PTMs as can be predicted in silico. (C) Single confocal z-planes (*BMPR2*^*wt*^ = basal; *BMPR2*^*ΔE2*^ = medial) showing stainings of FBN1 (red) and LAP (green) in intact cells (i). Figure enlargements are indicated by white frame (ii). Exemplary co-localization is indicated by white arrowheads (lower). Line scans (blue line; ii) for fluorescence intensities of FBN1 (red) and LAP (green) are indicated (iii). (D) Maximum projections of confocal stacks of decellularized ECM showing staining of FBN1 (red) and LAP (green). (i) Lack of DAPI staining indicates successful decellularization (ii) for indicated cell clones. Figure enlargements are indicated by white frame (ii). Exemplary co-localization shown by white arrowheads (lower). Line scans (ii) for fluorescence intensities of FBN1 (red) and LAP (green) are indicated (iii). (E) Cartoon describing bioassay using stable CAGA_12_-Luc TGFβ reporter cells and acidification of conditioned SNs. (F) TGFβ bioassay showing *firefly* luciferase values relative to *renilla* luciferase values (in RLU) for conditioned SNs of WT and BMPR2-deficient cells that were added to reporter cells either untreated (left), or when same conditioned SNs were treated with acid to activate latent complexes of TGFβ (middle) and expressed as relative ratio of acidified to nonacidified showing increase in latent TGFβ in conditioned SN of BMPR2-deficient cells (right). (G) Scheme depicting the strategy of decellularization of BMPR2-mutant ECM and re-cellularization to report on ECM-bound TGFβ by use of a bioassay. Cells were left to produce ECM followed by specific decellularization method. (H) Decellularized ECM was re-cellularized with HEK293T cells transiently transfected with a TGFβ reporter construct (CAGA_12_-Luc) (Kruskal-Wallis test with post hoc Dunn test, *n* = 12) (left) or when reporter cells were concomitantly overexpressing ITGB1 or when cells were additionally cultured in presence of either DMSO or ROCK inhibitor Y-27632 (20 μM; right) (two-way ANOVA with post hoc Bonferroni relative to DMSO, *n* = 3–4). In all panels, data are shown as mean + SD; *P < 0.05, **P < 0.01, ***P < 0.001, ****P < 0.0001. See also S7E Fig and S8 Data for underlying data. BMP, bone morphogenetic protein; BMPR2, BMP type-2 receptor; EC, endothelial cell; ECM, extracellular matrix; FBN1, fibrillin-1; LAP, latency-associated peptide; LLC, large latency complex; LTBP, latent transforming growth factor beta-binding protein; PTM, post-translational modification; RLU, relative light unit; ROCK, Rho-associated kinase; SLC, small latency complex; SN, supernatant; TGFβ, transforming growth factor-beta; WT, wild type.

LAP-TGFβ is also called small latency complex (SLC). Both the LAP and TGFβ are synthesized as a single pro-peptide cleaved prior to secretion. SLC is tethered to FBN1 microfibrils by binding to LTBP, which forms the large latency complex (LLC) integrating SLC into higher ordered FBN1 assemblies (Fig 8A) [83].

In this LLC, the growth factor adopts a straightjacket conformation, that opens up and releases mature TGFβ upon integrin-dependent tensile forces acting on LAP [85]. A number of different alpha-beta integrin complexes have been proposed to allow for LAP binding and mechanical TGFβ activation with β1-integrin–containing complexes shown in the context of tissue fibrosis [86]. To investigate whether increased β1-integrin-ILK mechano-signaling and FBN1 microfibrils result in controlling the bioavailability of LAP-TGFβ for BMPR2-deficient cells, we analyzed LAP presence in EC extracts using an antibody specific to LAP of TGFβ1. We identified increased levels of LAP of TGFβ1 (Fig 8B). This is intriguing since TGFβ1 expression remained unaltered in BMPR2-deficient ECs (S4B Fig) suggesting its extracellular accumulation. In line with this, we also found LAP1-FBN1 co-localization at medial z-planes of BMPR2-deficient cells (Fig 8C) as well as the SLC adapter protein LTBP-1, which localized with punctate pattern to cell junctions of BMPR2-deficient cells (S9A Fig). To investigate this further, we employed detergent-free decellularization to first confirm preservation of LAP-TGFβ bound to higher ordered FBN1 fibers (Fig 8D and S7E Fig). To gain more insights into the expression-independent increase in extracellular LAP-TGFβ1, we harvested conditioned media after 6–9 d of steady-state culture, from which we already knew that BMPR2-deficient cells also contain increased FBN-1 protein (Fig 6 B). With these conditioned TC supernatants, we then performed TGFβ bioassays using luciferase reporter cells carrying a SMAD3-luciferase reporter construct (CAGA-Luc). To activate potential latency complexes of TGFβ present in conditioned SN, we performed acidification followed by neutralization to chemically disassemble the SLC (Fig 8E and 8F). In line with our previous results for nonacidified conditioned SN, we found reduced bioactivity of TGFβ reporter cells with SN from BMPR2-deficient cells, suggesting their strong consumption of active TGFβ (typically delivered by serum supplements such as fetal calf serum [FCS]) under steady-state conditions (Fig 8F). However, after acidification, we found increased levels of active TGFβ present in conditioned SN from BMPR2-deficient cells, which confirms increased accumulation of de novo–formed latency complexes within conditioned media. These latency complexes are found in the supernatant, most likely bound to smaller FBN-1 deposits and a consequence of basal ECM saturation. To therefore test the accumulation of latency complexes within the decellularized ECM, we applied a strategy by which we repopulated decellularized ECM of BMPR2-deficient cells with TGFβ reporter cells (Fig 8G) showing that ECM of BMPR2-deficient cells is indeed rich in latent TGFβ (Fig 8H, left). To finally prove that our proposed mechanism of β1-integrin-actomyosin–dependent forces facilitating TGFβ release from the ECM of BMPR2-deficient cells, we further compared luciferase activity when reporter cells were co-transfected with β1-integrin or when ROCK was concomitantly inhibited. These experiments revealed that the reporter cell response is significantly increased when β1-integrin was co-expressed and reduced, when cells were concomitantly exposed to the ROCK inhibitor Y-27632 (Fig 8H, right). Together, this proves the increased production of latent TGFβ complexes by BMPR2-deficient cells, which we propose to be deposited as part of higher ordered assemblies with FBN-1 either as basal/junctional ECM deposit or secreted into the growth medium. Additionally, our experiments suggest that ITGB1 (in complex with a suitable alpha subunit) together with ROCK pathway activity is sufficient to activate those ECM-bound latent complexes of TGFβ.

## Discussion

Perturbations in BMP/TGFβ signaling are linked to a wide spectrum of diseases with a characteristic switch in the balance between both pathways in favor of increasing responses toward TGFβ. This is observed in cancer, fibrosis, and (cardio)vascular disorders, including PAH. In PAH, few ECs are resistant to apoptosis and instead proliferate and build up a cell pool, which is suggested to contribute to important stages of lesion development [6,87]. Intriguingly, these cells show a phenomenon recapitulated in a cell model presented here, in which BMPR2 was deleted by monoallelic mutations identified in HPAH patients. These BMPR2-deficient ECs gain TGFβ signaling responses. Moreover, blocking the TGFβ pathway reduced PAH in preclinical animal models [88]. Beyond that, little was known about how this gain in TGFβ signaling establishes in response to BMPR2 loss and whether cell-matrix interactions and integrin-dependent cell mechanobiology are involved.

**Fig 9:**
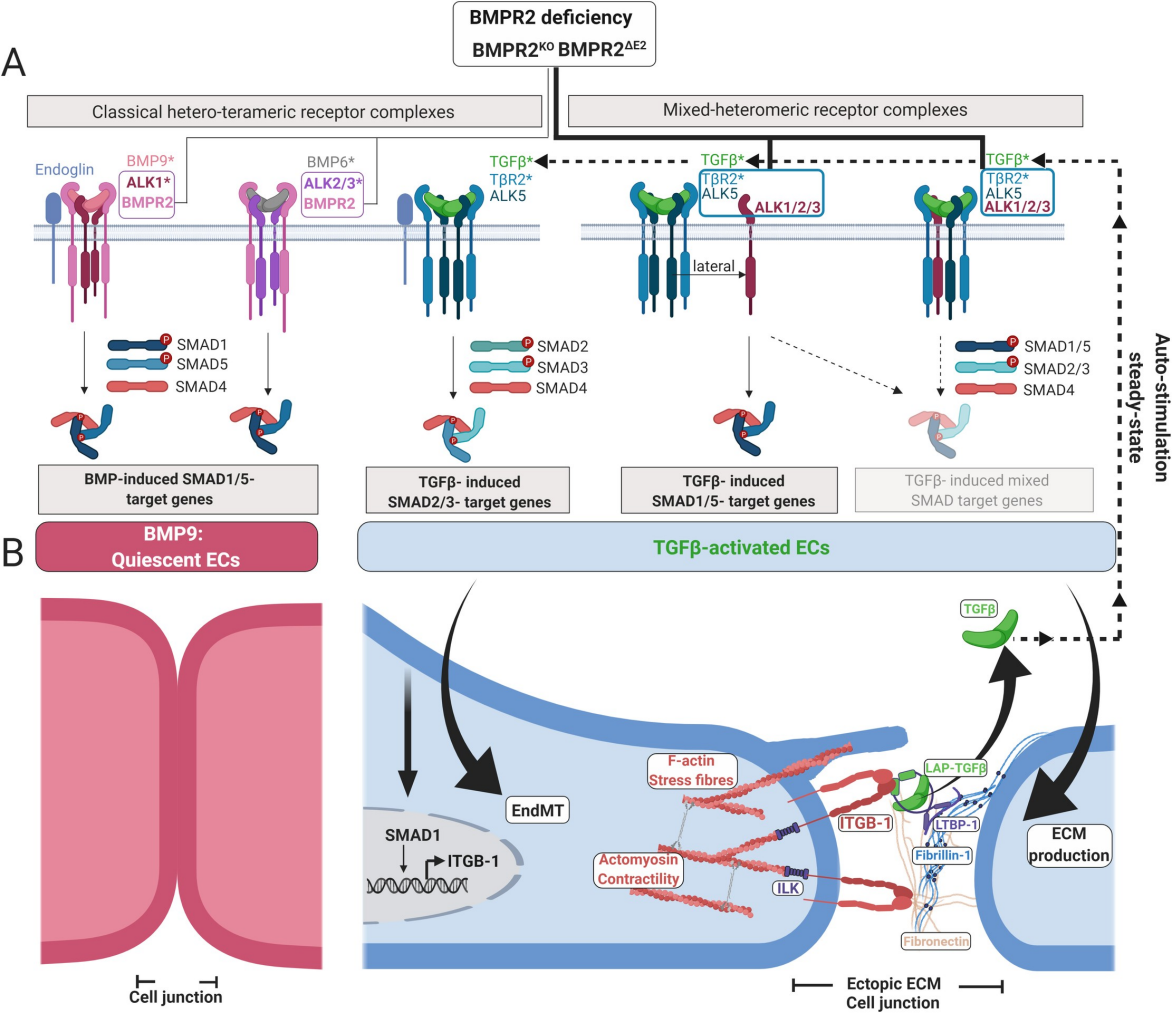
BMPR2 acts as a gatekeeper to protect ECs from activating TGFβ responses. (A) In quiescent ECs, the major BMP delivered to ECs via the blood stream is BMP9/heterodimers of BMP9/10. The majority of BMP9 (BMP9/10) signals are transduced via ALK1/BMPR2 heteromeric receptor complexes (high affinity interactors indicated by asterisks) to induce BMP-SMAD1/5 target genes. In case of BMP6 bioavailability, as, e.g., suggested over the course of activating EC sprouting angiogenesis, BMP6 signals in conjunction with ALK2/3 and BMPR2. At the same time, bioavailability of TGFβ is supposed to be relatively low. Biologically active TGFβ signals via complexes comprising TβR2 and ALK5 to induce TGFβ-SMAD2/3 target genes. Another possible route by which TGFβ signals in ECs is via complexes comprising ALK1 (possibly also ALK2/3 when surface levels relative to ALK1 increase) allowing for phosphorylation of SMAD1/5. It was suggested that this lateral route of TGFβ signaling requires ALK5. Two possible mixed-heteromeric receptor complex formation modes are suggested: (1) the BMP and TGFβ type-1 receptors are in higher ordered neighboring oligomeric assemblies or (2) BMP and TGFβ type-1 receptors occupy TGFβ binding sites within the same complex. Eventually, these two modes do occur concomitantly in spatial and temporal proximity, e.g., as part of a clustering event. Additionally, these mixed-heteromeric assemblies are likely involved in the formation of mixed-heteromeric SMAD complexes of which little is known regarding their functionality. Upon BMPR2 deficiency, the equilibrium of these receptor complexes switches toward increased formation of TGFβ binding receptor complexes. This is because the gatekeeper function of BMPR2 is impaired. (B) BMPR2-deficient ECs gain responsiveness to TGFβ but also show increased TGFβ autostimulation when cultured under steady-state conditions. This is due to increased mechanical properties of BMPR2-deficient ECs, which co-emerge with their EndMT, the resolution of their junctional architecture, and remarkable changes in their mechanical features. As such, SMAD1 induces β1-integrin (*ITGB1*) expression, and ITGB1 translocates with the actomyosin scaffolding protein ILK to CCC sites, which stiffen as a result of increased stress-fiber F-actin. As a consequence of an altered matrisome expression profile, we find at CCC sites deposits of FBN1 and increased remodeling of FN. We show that latency complexes of TGFβ tether to these ECM deposits and that they can be efficiently activated by BMPR2-deficient ECs. This is facilitated extracellularly through ITGB1 binding to LAP-(TGFβ1) and intracellularly by increased ROCK-dependent actomyosin contractility acting on ILK-ITGB1. We propose that this mechanical activation of TGFβ allows continuous autostimulation for cells, which integrates into the observed increase in TGFβ responses upon BMPR2 deficiency. ALK1, activin receptor-like kinase 1; BMP, bone morphogenetic protein; BMPR2, BMP type-2 receptor; CCC, cell-to-cell contact; EC, endothelial cell; ECM, extracellular matrix; EndMT, endothelial-to-mesenchymal transition; FBN1, fibrillin-1; F-actin, filamentous actin; ILK, integrin-linked kinase; ITGB1, integrin subunit beta 1; LAP, latency-associated peptide; ROCK, Rho-associated protein kinase; SMAD, suppressor of mothers against decapentaplegic; TβR2, TGFβ type-2 receptor; TGFβ, transforming growth factor-beta.

TGFβ is retrieved as an active growth factor from its latent ECM-bound complex through locally controlled mechanical forces involving integrins [80]. HPAH is caused by mutations in *BMPR2*, and HPAH lesions resemble typical features reminiscent of TGFβ-dependent fibrosis. In this context, the concept of integrin-dependent TGFβ retrieval is well established. We therefore hypothesized that ECs with BMPR2 deficiency could be a suitable cell model to stress this hypothesis. BMPR2 loss would causally imply loss of BMP-SMAD signaling. However, endothelial specific animal models of BMPR2 deficiency and cellular knock-down studies suggest that other BMP type-2 receptors might compensate for BMPR2 deficiency in ECs with respect to BMP ligand binding and SMAD signaling [34,89,90]. To characterize our endothelial BMPR2-deficiency cell model, we stimulated cells with ectopic recombinant ligands and indeed observed increased BMP6 and no change to mild reduction in BMP9 signaling capacity (Fig 1B and 1D). Due to the picomolar affinity of BMP9, this slight decrease was only observed for BMPR2^KO^, which exhibited lower ALK1 levels compared to BMPR2 truncation mutants (S4B Fig). This remaining BMP signaling activity can only be explained by compensation for loss of BMPR2 by other BMP-binding type-2 receptors such as ACTR2A or ACTR2B. In a previous study, Cre-mediated deletion of *Bmpr2* in SMCs revealed that BMP6 gained signaling activity via ACTR2A together with a set of type-1 receptors distinct from those that would be utilized by BMPR2 [89]. Here, we proposed that signaling initiated by recombinant BMP6 is mediated via ALK2/3 complexed with ACTR2A, while signaling initiated by recombinant BMP9 is mediated via ACTR2B-ALK1 [91] (Fig 1B). *In vivo*, BMP9 (or heterodimers of BMP9/10) would be the main endothelial ligand available through the plasma [32] contributing to EC homeostasis [92]. Thus, BMPR2 deficiency most likely affects EC BMP9/10 signaling. This is why current therapeutic attempts focus on BMP9 treatment, with BMP9 proposed to reverse PAH in BMPR2 deficiency and hypoxia PAH models [93]. According to our results, one explanation for a successful reversion of PAH by BMP9 application would be that BMP9 would serve as an antagonist for TGFβ by competing with TGFβ for binding ALK1 and ALK2 in a mixed-heteromeric receptor complex. By targeting *BMPR2* in ECs, we discovered that this receptor unexpectedly served as a gatekeeper, inhibiting under normal conditions lateral signaling by TGFβ-induced SMAD1/5 and the formation of mixed SMADs (Fig 9A).

Even though lateral TGFβ signaling was reported before to occur in several cell types (reviewed in [39]), loss of BMPR2 clearly promotes this alternative TGFβ-signaling route in ECs. We propose that this mode of action becomes particularly important with increasing TGFβ bioavailability.

BMPR2 expression levels are regulated beyond genetic factors, and low expression is found in IPAH but also a variety of other vascular diseases, including arteriosclerosis (reviewed in [15]). Moreover, even homozygous *Bmpr2* deletion in the mouse endothelium is not sufficient to cause PAH in all mice, suggesting that additional—yet less well understood—factors/triggers add up to the pathogenesis [94]. Suspected factors regulating BMPR2 expression are hypoxia, inflammation, and possibly stiffness [95,96]. Those environmental effects arise over the course of PAH lesion formation and are confirmed in our steady-state cultures. In support of this, we find besides higher stiffness of BMPR2-deficient cells (Fig 4G), a significant up-regulation of interleukin-6 (IL-6) (S9B Fig), a pro-inflammatory cytokine suggested to suppress BMPR2 expression [97]. We therefore speculate that loss of BMPR2 creates a dys-balance in BMP and TGFβ signaling that results in cellular adaptation (e.g., SMAD1 up-regulation and/or ENG-ALK1 down-regulation) but also accumulation of different ECM molecules (e.g., as seen by increased FN, TNC, FBN-1 deposition) and a remarkable mechanical adaptation. Concomitant down-regulation of BMPR2 surface levels under compromised conditions would thus allow cells to recruit other type-2 receptors (TβR2, ActR2) into a functional mixed-heteromeric receptor complex involving ALK5 and ALK1/2 (Fig 9A). The gatekeeper function of BMPR2 would therefore be to maintain BMP type-1 receptors in complexes responsive to BMP ligands, while its loss favors engagement of BMP type-1 receptors such as ALK1 and possibly also ALK2 to form heteromeric complexes together with TβR2 and ALK5 facilitating increased responsiveness toward TGFβ ligands. We have investigated here the downstream pathway alterations on the level of SMADs. Intrinsic structural properties of type-1 receptors determine which R-SMAD is phosphorylated by the activated R1 kinase [50]: ALK4, ALK5, and ALK7 phosphorylate SMAD2/3, and ALK1, ALK2, ALK3, and ALK6 phosphorylate SMAD1/5. BMPR2-deficient ECs clearly maintained SMAD1/5 but gained TGFβ-induced SMAD2/3 and SMAD1/5 (lateral)—signaling and formation of (mixed)-SMAD1/2 complexes, the latter having unknown functions in PAH. Using complementary approaches involving SMKIs, RNA interference, and dose-response kinetics, we show that, in BMPR2-deficient ECs, (1) the type-2 receptor position is occupied by a TGFβ-binding receptor, i.e., TβR2; (2) ALK2/ALK1 and ALK5 are the receptors occupying the R1 positions; and (3) ACTR2 may replace TβR2 when Activin A is present. This gain in TGFβ responsiveness in the absence of BMPR2 accounts for TGFβ-signaling routes clearly distinct from TGFβ signaling in the presence of BMPR2. Because the relative ratio of ALK2/ALK1 is increased in BMPR2-deficient cells (S6B Fig), we propose ALK2—as yet understudied in ECs—to be an important receptor, with its role potentiated upon reduced ALK1 expression (Figs 1G and 9A, and S2A Fig). The role of BMPR2 as an inhibitor of Activin-SMAD1/5/8 signaling was recently shown in myeloma cells [98]. Our current study provides insights into the molecular properties of BMPR2 but furthermore establishes new roles for this receptor adding up to previous observations by others. For example, the fibrodysplasia ossificans progressiva (FOP)-causing mutation in ALK2 results in a hypersensitive receptor, still requiring BMPR2 within the functional receptor complex [99]. The central role of type-2 receptors is also highlighted by the gain in lateral Activin-SMAD1/5 signaling due to engagement of ACTR2 into the functional FOP-ALK2 signaling complex [100]. From these and our study here, we propose that the level of BMPR2 in cells is indicative of balanced TGFβ/(Activin)/BMP signaling and thus indicative of maintenance of cell fate, making BMPR2 a central gatekeeper molecule (Fig 9A). In that context, it seems intriguing that increased lateral signaling by TGFβ as well as increased formation of mixed SMAD complexes is found in the developing mouse lung and that mixed SMAD complexes are more prevalent in murine embryonic development and human breast cancers than in normal adult physiology [52]. Therefore, it can be concluded that the proposed gatekeeper function of BMPR2 is a function of mature cells, which is impaired in cells that are not terminally specified and/or in a pathological transition process. This fits to our observations that BMPR2-deficient ECs undergo EndMT. These conclusions are further underlined by research in epithelial cells and epithelial cancer cells. Here, TGFβ was shown to also signal via lateral routes [40] required for EMT via both the SMAD3 and SMAD1/5 pathways [41].

Since FBN1—the major component controlling extracellular TGFβ sequestration—is strongly up-regulated in HPAH cells, the shift toward TGFβ signaling in our system is additionally promoted via an extracellular circuit involving the matrisome whose integration into BMPR2 deficiency is relatively little understood. A previous RNA-Seq study using pulmonary arterial ECs from PAH donors identified COL4 as a major EC basement membrane protein down-regulated upon BMPR2 deficiency [101]. Although we also saw a reduction in COL4, we could show that ECM proteins indicative of fibrosis and possibly myo-fibroblastic transition (FN, FBN-1, TNC) are up-regulated. It is of special interest that different vascular diseases are caused by mutations in either *FBN1*, *TβR2* (Marfan and Loeys-Dietz syndromes) or *BMPR2* (HPAH), and *ALK1* or ENG (HHT). In our model, FBN1 was up-regulated, while ALK1 and ENG were down-regulated as a consequence of BMPR2 deficiency (Fig 2E), emphasizing the importance of the ECM on one hand and the BMPR2-ALK1-ENG signaling axis on the other to maintain the EC phenotype. As a result of BMPR2 deficiency, ECs obtain a characteristic transcriptional profile via the concerted action of canonical, lateral, and possibly mixed SMAD complexes regulating mechano-relevant genes and the cell’s matrisome. While it is known that EndMT is induced in PAH (VE-Cadherin to N-Cadherin switch), we show additionally that mechanical properties of ECs change along this process. This is highlighted by (1) a specific expression pattern of integrins, (2) distinct expression and localization of ECM, and (3) mechanical adaptation via actin-actomyosin networks. However, we cannot rule out involvement of additional, e.g., non-SMAD pathways, regulating mechanical aspects of BMPR2-deficient cells. This is intriguing, because BMPR2 is associated to a number of cytoskeleton pathways, including LIM domain kinase, an upstream regulator of cofilin that we also find de-regulated (Fig 4F) [102], and phosphoinositide 3-kinase (PI3K) signaling. PI3K signaling was shown by us before to regulate BMP-BMPR2–dependent actin reorganization [103] and by others to regulate EC actomyosin contractility [104], which is heavily deregulated in our model (Figs 4F and 5D, and S5E Fig). Besides FBN1, β1-integrin plays a central role seemingly regulated by SMAD1. The up-regulation of ITGB1 under steady-state culture conditions is dependent on ALK5 and ALK1/2 kinase activity and SMAD1 binding to the ITGB1 promoter (Fig 3E, 3F and 3G). However, increased lateral signaling by TGFβ under steady-state conditions would imply stronger accumulation of—and autostimulation with—active TGFβ, independent of TGFβ expression, since it was not altered in BMPR2-deficient cells (S4B Fig). Indeed, we found that BMPR2-deficient ECs consume more TGFβ than WT cells, while they additionally deposit more LAP (TGFβ) into the TC supernatant (Fig 8F) and into ECM deposits (Fig 8H). Indeed, we could show that integrin-dependent TGFβ retrieval from extracellular FBN depots is increased in BMPR2-deficient cells (Fig 8H). Together with the altered ECM expression, this could create a self-stimulatory cycle for lateral TGFβ signaling of BMPR2-deficient ECs (Fig 9B). The fact that we find increased TGFβ-induced SMAD2/3 and lateral Smad1/5 signaling even upon addition of active, ectopic ligand suggests an additional mechanism that must be independent of mechanical integrin-dependent TGFβ activation. The up-regulation of SMAD1 and SMAD2 is intriguing here, while SMAD3 expression, in line with reports by others, is impaired upon BMPR2 deficiency (Fig 1E) [105]. We suggest this to be an important aspect of cell-autonomous adaptation in response to BMPR2 deficiency. Moreover, we also found—at higher doses of BMP9 (300 pM) and BMP6 (10 nM)—a BMP-dependent phosphorylation of SMAD2 in BMPR2-deficient cells (Fig 1D) and, for BMPR2^ΔE2^ cells, also a stronger BMP6-dependent induction of CTGF (Fig 1F). Of note, these experiments are conducted upon addition of recombinant ligands. For the self-stimulatory integrin-dependent retrieval of active TGFβ, however, this raises the question of whether TGFβ is locally consumed or whether it signals to cells in spatial proximity. Our system so far lacks the interface with other vascular cells, e.g., SMCs. A recent study on the micro-milieu character of mechano-dependent TGFβ retrieval suggests very localized consumption of retrieved TGFβ [106,107], suggesting that ECs themselves benefit more from the actively retrieved growth factor. Disconnecting local cellular responses from the extracellular environment by blocking integrin activity may thus open new routes to target EC pathology.

Changes in visco-elastic properties, stiffening, and crosslinking of the ECM is associated to mid- to end-stage HPAH lesions [108,109]. The balance between adhesion forces at the cell-substratum interface and cell contractility at the CCC interface is postulated to regulate endothelial function [110,111]. However, a significant part of the contractile energy is transferred to actin-network dynamics [112]. Besides higher traction forces toward FN, we also found that the F-actin cytoskeleton is altered in BMPR2-deficient ECs. F-actin characterization spatially (S4F Fig and S1–S3 Movies) and mechanically (Fig 4G and 4H), including its relative orientation to the cell junctions (Fig 4H and S5F Fig), recalls Sullivan’s concept on *form follows function* (FFF) in design and architecture. We can extend it here toward *form follows function follows form* (5F), underlining the intertwined character of ECs’ extracellular and intracellular structural adaptation in response to loss of BMPR2 as a gatekeeper.

These cellular data are further underlined by our observations in PAH lesion sites of low–BMPR2-expressing human donors, where concomitant appearance of more intact FBN-1 deposits, contractile inner luminal cells, and LTBP-1 are most prevalent when grade III lesions display still intact inner and outer elastic membrane. This may create a similarly confined microenvironment for cells to adapt while environmental factors accumulate. In contrast, in advanced grade IV plexiform lesions, the layered tissue architecture is resolved, and discrete tissue borders are absent (Fig 7D). Here instead reduced FBN-1 deposits were described in pulmonary artery [113]. Interestingly, plexiform lesions of HPAH only are characteristic for clonally expanded ECs that display microsatellite site mutations and reduced protein expression of TβR2 [114]. Thus, TβR2-negative, clonally expanded, BMPR2-deficient ECs in the intima of plexiform lesions would not transmit lateral TGFβ signaling, since TβR2 would be strictly required. This is suggesting that the here-proposed molecular mechanism accounts for EC dysfunction during earlier stages of lesion formation in HPAH.

Together, we report here on a new human BMPR2-deficient endothelial model. We propose a so far unrecognized concept by which BMPR2 acts as a gatekeeper for homeostatic and balanced BMP/TGFβ signaling in ECs protecting them from increased responses toward TGFβ (Fig 9A). We provide evidence that loss of BMPR2 alters biophysical properties of ECs and the matrisome, which integrates TGFβ-SMAD and integrin signaling into an accelerating feed-forward loop for activating TGFβ responses in endothelial dysfunction (Fig 9B).

## Materials and methods

### Ethics statement

Lung tissue biopsies were procured at the registry of the UGMLC-Giessen biobank (Justus-Liebig University Giessen, Germany; Collaborative Research Center 1213), member of the DZL (German Center for Lung Research) Platform Biobanking. The samples were collected in compliance with ethical practices and provided to PK in accordance with necessary requirements to protect the full privacy of donors. Biobank Giessen holds documents on informed consent of donors. The ethics committee of the federal state Berlin approved the methods used to analyze human PAH samples provided to PK (EA2/082/19).

### Expression plasmids

The coding sequence of human BMPR2 was C-terminally myc-tagged and subcloned into pcDNA3.1 from HA-BMPR2-LF plasmid as described previously [115]. Exon 2 of BMPR2 was deleted using Pfu-Polymerase (#E1114-02, EURx) following the instructions of the QuikChange Site-Directed Mutagenesis Kit (Agilent). In order to generate a bidirectional Firefly-Renilla-Luciferase Plasmid that reports on SMAD3 transcriptional activity and baseline expression of Renilla as internal control, the promoter sequence and luciferase gene of *(CAGA)*_*12*_*‐MLP‐Luc* was subcloned into *pMuLE-ENTR-MCS-L1-R5* (Addgene plasmid #62084) [116]. Using Gateway recombination cloning, *pENTR L1-(CAGA)12‐MLP‐Luc-R5* was recombined with *pMuLE-ENTR CMV-Renilla-Luciferase-L5-L2* (Addgene plasmid #62186) and *pLenti-X1-Puro-DEST (694–6)* to generate *bi-directional (CAGA)*_*12*_*‐MLP‐Luc-Renilla* following the protocol described in Albers and colleagues [117]. *pLenti-X1-Puro-DEST (694–6)* was a gift from Eric Campeau and Paul Kaufman (Addgene plasmid #17297*)*. *pMuLE-ENTR-CMV-Renilla-Luciferase-L5-L2* and *pMuLE-ENTR-MCS-L1-R5* were a gift from Ian Frew. Used primers are listed in S1 Table.

### CRISPR/Cas9 generation of stable cell clones

CRISPR design interface (http://crispr.mit.edu/) together with CCTop (https://crispr.cos.uni-heidelberg.de/) was used to design sgRNA and evaluate potential off-targets. The pSpCas9(BB)-2A-Puro-(PX459)-v2.0 was a gift from Feng Zhang (Addgene plasmid #62988) and used as cloning backbone for CRISPR/Cas9 targeting as described in Ran and colleagues [118]. Briefly, phosphorylation and annealing were performed with the oligonucleotides listed in S1 Table harboring a BbsI overhang. Afterwards, BbsI (#R0539S, NEB) digestion and ligation was performed according to the manufacturer’s protocol. Plasmids were validated by sequencing. EAHy926 cells were seeded (50,000 cells/24 wells) and transfected with pSpCas9(BB)-2A-Puro-BMPR2-E2 and JetPEI transfection reagent (Polyplus, New York) following the manufacturer’s instructions. Cells were selected using puromycin (3 μg/mL), followed by recovery in M199 basal growth medium prior to clonal expansion using single-cell dilution in 96-well plates. pSpCas9(BB)-2A-Puro-eLacZ was used as negative control. Clonal validation was performed with genotyping PCR on genomic DNA and complementary DNA, with primer pairs indicated in S1 Table.

### Cell culture

EAhy926 cells were maintained and expanded in M199 medium (Sigma Aldrich) supplemented with 20% FCS (Biochrom, Germany), 2 mM L-glutamine (PAN-Biotech, Germany), 100 units/ml penicillin, 10 μg/ml streptomycin (PAA Laboratories), 25 μg/ml Heparin (Sigma Aldrich), and 50 μg/ml EC growth supplement (Corning, NY), hereafter referred to as M199 basal growth medium, at 37°C and 5% CO_2_ atmosphere. Starvation of cells was carried out after rinsing cells in phosphate buffered saline (PBS) (PAN-Biotech GmbH, Germany) and exposure to M199 media containing 100 units/ml penicillin, 10 μg/ml streptomycin, and 25 μg/ml Heparin for 6 h. For maintenance and expansion, typically 500,000 cells were seeded in T75 TC plastic flasks (Greiner Bio-One International) and passaged using Trypsin (PAN-Biotech, Germany) (unless stated otherwise) in 4 d intervals, with cells reaching confluence after day 2–3 upon seeding. For assays (unless stated otherwise), we seeded 50,000 cells per well in a 12-well plate (or coverslip in same format) with cells left for 4 consecutive days (reaching confluency at day 2) to expand without change of the growth media (hereafter referred to as steady-state culture conditions). At day 4, cells were harvested, starved, stimulated, and lysed or fixed for subsequent analysis. Automated cell count was conducted using CASY Model-TT cell-analyzer (Roche, Germany) and included monitoring of cell viability and proliferation capacity from passage 4 to passage 40. Experiments were carried out at passages 7–40 after clonal selection. HEK293T and Cos7 cells were maintained and expanded in DMEM (Biochrom, Germany) containing 1.0 g/l D-glucose, 2 mM L-glutamine, 100 units/ml penicillin, 10 μg/ml streptomycin, and 10% FCS.

### Transient transfection with expression plasmids and siRNA

Transfection of HEK293T cells was carried out using the polyethylenimine (PEI) method [119]. For transfection of 20 nM siRNA SmartPools TβR2 (L-003930-00-0005), FBN1 (L-011034-00-0005), or control (D-001810-10-05) (Dharmacon), Lipofectamine2000 (ThermoFisher Scientific) was used according to manufacturer instructions.

### Cell stimulation with growth factors and SMKI treatment

For cell stimulation with rhBMP6 (S. Vukicevic, University of Zagreb, Croatia), rhBMP9 (PeproTech, Hamburg, Germany), rhTGFβ-1 (PeproTech), and rhActivin-A (R&D Systems), growth factors were reconstituted and stored according to manufacturer instructions. For small-molecule–based inhibition (SMKI), K02288 (Alex Bullock, Oxford, UK) [120] and SB-431542 (Sigma-Aldrich) [47] were added to cells 1 h prior to ligand stimulation with indicated concentrations unless stated otherwise. For long-term Rho-kinase inhibition, Y-27632 (Stemcell Technologies) was added at the indicated concentration at time of cell-seeding and 12–48 h later.

### Antibodies

All antibodies used for WB were prepared in TBS-T containing 3% w/v bovine serum albumin (BSA)/fraction V (Carl Roth). WB was conducted as previously described [103]. All WB raw data are deposited in S1 raw images. Antibodies for immunocytochemistry (ICC) were diluted in PBS containing 1% w/v BSA and 3% v/v normal goat serum (Abcam). Antibody dilutions for PLA were prepared in DuoLink antibody dilution buffer (Sigma Aldrich). Respective dilutions are indicated as follows: phosphorylated SMAD1/5, clone 41D10 (WB 1:1000; Cell Signaling Technologies); phosphorylated SMAD2, clone 138D4 (WB 1:1000; Cell Signaling Technologies); SMAD1, clone D59D7 (WB 1:1000; Cell Signaling Technologies, ChIP 5 μg/immunoprecipitation); SMAD1, AB55476 (PLA 1:200; Abcam); SMAD2 clone 86F7 (WB 1:1000; PLA 1:200; Cell Signaling Technologies); SMAD2/3 clone 18 (WB 1:1500; PLA 1:200; BD Biosciences); SMAD5 12167-1-AP (WB 1:1000; PLA 1:100; Proteintech); SMAD4 clone D3R4N (WB 1:1000; PLA 1:800; Cell Signaling Technologies); ILK clone 65–1 (WB 1:500; ICC 1:50; PLA 1:50; Santa Cruz Biotechnology); β1-integrin (CD-29) clone 18 (WB 1:1000; BD Biosciences); β1-integrin clone P5D2 (ICC 1:500; Abcam); phosphorylated β1-integrin-Ser785 AB8124 (WB 1:10000; PLA 1:500; Merck Millipore); phosphorylated β1-integrin-Tyr783 AB8125 (WB 1:10000; Merck Millipore); phosphorylated paxillin-Tyr118 (ICC 1:100; Cell Signaling Technologies); N-Cadherin clone 32 (ICC 1:250; BD Biosciences); VE-Cadherin clone D87F2 (WB 1:1000); phosphorylated VE-Cadherin-Tyr685 CP1981 (WB 1:1000; ECM Biosciences); VE-Cadherin clone BV6 (ICC 1:200; Merck Millipore); PECAM-1 (CD-31) clone 89C2 (ICC 1:3000; immunohistochemistry [IHC]: 1:250; Cell Signaling Technologies); LTBP-1 (IHC: 1:1000; the antibody was previously described [121]); β-Catenin clone D10A8 (ICC 1:250; Cell Signaling Technologies); αSMA (IHC: 1:250 ab5694, Abcam); MLC 2 #3672 (WB 1:1000; Cell Signaling Technologies); pMLC-Ser19 #3671 (WB 1:1000; ICC 1:50; IHC: 1:50; Cell Signaling Technologies); phosphorylated cofilin-Ser3 clone 77G2 (WB 1:1000; Cell Signaling Technologies); LAP (of TGFβ-1) clone 9005 (WB 1: 1000; ICC 1:250; R&D Systems); TβR2 clone C-4 (WB: 1:500; Santa Cruz Biotechnology); BMPR2 (ICC 1:200; Cell Signaling Technologies); BMPR2 clone-18 (WB 1:1000; BD Biosciences); FN ab23750 (ICC 1:50; Abcam); GAPDH clone 14C10 (WB 1:1000; Cell Signaling Technologies); β-Actin clone AC-15 (WB 1:5000; Sigma Aldrich); myc-tag clone 9B11 (WB 1:1000; ICC 1:8000; Cell Signaling Technologies); and TGFβ-1,2,3 MAB1835 (WB 1:1000; R&D Systems). Polyclonal rabbit anti-FBN1 antiserum was raised against the C-terminally His6-tagged rF90 representing the N-terminal half of FBN1 in 293 Epstein-Barr virus nuclear antigen (EBNA) cells [122]. The antiserum was purified by affinity chromatography on a column with antigen coupled to cyanogen bromide–activated Sepharose (GE Healthcare). FBN1 antibody was used at 1:1000 for ICC and IHC and at 1:2000 for WB. Polyclonal rabbit anti-LTBP-1 antiserum was raised against the C-terminally double-strep–tagged polypeptide rL1K representing the C-terminus of human LTBP-1 (Arg^1181^ to Glu^1394^). L1K was expressed in 293EBNA cells as previously described [83]. The antiserum was purified before usage by affinity chromatography on a column with antigen coupled to cyanogen bromide–activated Sepharose (GE Healthcare).

### Surface biotinylation

For biotinylation of surface proteins from transiently transfected Cos7 cells, 20,000 Cos7 cells were seeded per centimeter squared of a 10-cm dish in DMEM containing 10% FCS, 2 mM L-Glutamine, 100 units/mL penicillin, and 10 μg/mL streptomycin. Cells were left to adhere before transfection of indicated expression vectors was performed using the PEI method. For surface biotinylation of endogenous proteins, ECs were seeded at 20,000 cells/cm^2^ in M199 basal media 2 d prior to the experiment. The general cell-surface biotinylation protocol is described in [123]. Cell surface proteins were biotinylated using cell-impermeable sulfosuccinimidyl-6-(biotinamido) hexanoate (EZ-Link Sulfo-NHS-SS-Biotin, Thermo Scientific). Since all extracellularly exposed lysine residues are encoded in exon 2 of BMPR2, labeling of α-amino groups in the remaining extracellular domain of BMPR2ΔE2 had to be established [124]. This is achieved in pH lower than the typical reaction for NHS-ester reagents. With pKa = 8.9 for α-amino group being considerably lower than for ε-amino group of lysine (pKa = 10.5), lysine amines are less in the un-protonated and reactive state [124]. Biotinylation solution was prepared by solving 0.5 mg/mL EZ-Link Sulfo-NHS-SS-Biotin (Thermo Scientific) in biotinylation buffer (PBS, 10 mM MgCl2 [pH 6.5]) by stirring and warming to 37°C. After EZ-Link Sulfo-NHS-SS-Biotin labelling at 4°C for 50 min and quenching (3 times 3-min PBS [pH 8.0] containing 100 mM glycine), cells were lysed in RIPA buffer (150 mM NaCl, 25 mM Tris/HCl, 0.1% SDS, 0.5% NP-40 [pH 7.8]) containing 1 mM henylmethylsulfonylfluorid, 2 mM sodium orthovanadate, 50 mM sodium fluoride, and 1× EDTA-free complete inhibitor cocktail (Roche). Precipitation of biotinylated surface proteins was performed overnight by addition of 40 μl streptavidin-coupled sepharose beads (GE Healthcare). Samples were washed with fresh lysis buffer, eluted with 40 μl 2× Laemmli buffer, and subjected to WB.

### ICC

The amount of 5 × 10^4^ cells were plated on glass coverslips placed in 12-well plates. Cells were left to expand for 3–4 consecutive days to form a confluent monolayer (unless stated otherwise) before fixation with 4% paraformaldehyde (PFA) was performed. Immunofluorescence staining was performed as described in [125]. In brief, cells were permeabilized in 0.5% Triton-X-100 for 15 min at room temperature; after blocking for 1 h with a mixture of 3% w/v BSA and 5% v/v normal goat serum in PBS, cells were stained sequentially by using the indicated primary antibodies. Primary antibody binding was detected using sequential labelling with Alexa Fluor488 or 595 conjugated secondary antibodies (Invitrogen) for 1 h at room temperature. Phalloidin-Alexa594 or Phalloidin-Alexa680 was purchased from Invitrogen and used according to manufacturer instructions. DRAQ5 (Thermo Fisher) or 4′,6-diamidino-2-phenylindole (DAPI; Carl Roth) were used for nuclear counterstaining. Membrane staining of fixed adherent cells using Vybrant DiO cell labelling (Thermo Fisher Scientific) was performed according to manufacturer instructions.

### IHC of paraffin sections

For IHC stainings, 10-μm-thick serial sections of paraffin embedded human lung tissue (controls and PAH donors) were baked for 1 h at 37°C, washed 2 times for 30 min with UltraClear (Biosystems, Switzerland), and rehydrated in increasing Ethanol-Water series. Sections were then washed in Buffer I (1.5% final concentration hydrogen peroxide, 3.9 g citric acid, 10.2 g di-sodiumhydrogenphosphatedihydrate in 100 ml water) for 15 min. Antigen retrieval was performed by immersing sections in Buffer II (0.48 g Tris-base, 0.03 g EDTA, 100 ml water [pH 9]) and heating on high setting for 2 min in the microwave. Autofluorescence of the tissue was blocked by treating the slides in Sudan black solution (0.3% Sudan Black in 70% ethanol) for 20 min followed by washing in PBX (PBS + 0.1% Triton X-100). The tissue was permeabilized using 0.4% Triton X-100 (Sigma Aldrich) for 10 min. Sections were blocked in 5% BSA (Roth) in PBS for 1 h at room temperature. Primary antibody was incubated at 4°C either overnight (FBN1; alpha-SMA) or for 2 nights (PECAM-1, pMLC, LTBP1). For PECAM-1 and pMLC, the following modifications were made to the staining procedure: the sections were baked for 1 h at 60°C instead, and the heat-mediated antigen retrieval step was omitted. Primary antibodies were detected by incubation with anti-mouse, anti-rabbit (F(ab')2 fragments only), and anti-goat (H+L chain—highly cross-adsorbed) secondary antibodies (Alexa Fluor Dyes) for 1 h at room temperature. Secondary fluorescently labelled antibodies were used at 1:250–1:350 dilutions for both IHC. When strong specimen autofluorescence at 488 nm excitation was detected, secondary antibody emission in the infrared spectral range was chosen. Antibodies used for these experiments are listed in the antibody section. Specimens were counterstained with DAPI (Invitrogen) and mounted with FluoromountG (SouthernBiotech).

### PLA

Cells were seeded at 200,000 cells/cm^2^ in M199 basal medium on glass coverslips. Monolayers of cells were serum starved for 6 h, followed by stimulation with the indicated growth factors for 15 min. Subsequently, Duolink *in situ* proximity ligation (Sigma Aldrich) was performed as previously described [126]. Specificity of antibodies was verified by single antibody controls as well as positive control, i.e., TGFβ-dependent SMAD2/3-SMAD4 translocation into the nucleus.

### High-content PLA analysis

To retrieve quantitative ratios of SMAD-PLA signals in nucleus versus cytoplasm of confluent EC monolayers, we have established a customized ImageJ-based algorithm. For the channel depicting nuclei (DAPI staining), a Gaussian-blur filter (sigma = 2) followed by Huang-white autothreshold and watershed algorithm was applied to generate a mask for automated count of individual and well-separated nuclei by the “analyze particle” function. For the channel depicting PLA signals, image definition was increased by rolling-ball background subtraction method (radius of pixels = 50). The PLA channel was duplicated, and a Gaussian-blur filter (sigma = 2) was applied. The PLA channels were subtracted from each other, and autothreshold (default) was applied. For the final PLA signal channel, a median filter with radius of 2 pixels was applied to discriminate individual PLA particles for automated count using the count particle function. The number of PLA signals in the same ROI as the nuclear masks were considered nuclear PLA signals, whereas all signals outside of these masks were considered cytosolic PLA signals. Seven or more images were quantified per staining. The quantification was performed on one set of experiments, when all stainings were performed at the same time.

### Confocal microscopy

Confocal data were produced using an inverted Leica DMi8 CEL Compact semimotorized confocal scanning microscope with excitation by 405, 488, 552, and 638 nm diode lasers. All confocal data sets were imaged using a 40×/1.30 HC PL APO Oil CS2 WD 0.24 mm objective, and data were recorded by photomultiplier or hybrid detector.

### Wide-field microscopy and live cell imaging

Phase contrast and epifluorescence images were taken using an inverted fluorescence microscope Axiovert 200 with Cy2, FITC, Alexa594, and Cy5 excitation/emission filters and a 63× Zeiss plan apochromat oil immersion objective (Zeiss). Signals were recorded with CoolSNAP HQ2 EMCCD camera (Photometrics, Tucson, AZ). Images were processed using linear BestFit option of Axiovision software (Zeiss). Life Cell imaging of monolayers incorporating FN^rho^ (Cytoskeleton Inc., Denver, CO) was performed after seeding and incubation of ECs for 6 h in the presence of 20 μg/ml FN^rho^ on glass-bottom dishes (MatTek Corporation). A region of interest was chosen for subsequent video time lapse imaging using the Mark&Find macro in AxioVision Software (Zeiss). Time lapse imaging was performed for 6 h with 5 min time-frames between individual pictures with samples mounted on a motorized scanning table (Maerzhaeuser, Germany) equipped with a heat- (37°C) and CO_2_- (5%) controlled Life Cell Imaging chamber (ibidi, Germany) providing stable atmosphere.

### Scanning electron microscopy

Confluent monolayers of ECs were rinsed with PBS and subjected to a primary fixation using 2.5% glutaraldehyde in PBS for 30 min. The cells were washed 3 times in PBS and subjected to a secondary fixation using 4% PFA for 20 min. The cells were then dehydrated, using serial dilutions of ethanol (25%, 50%, 75%, 90%, and 99.9% for 5 min each). After the last dehydration step, the samples were covered with a drop of 99.9% ethanol, placed into a desiccator, and heated at 30°C for 12 h. Prior to imaging, the samples were sputter coated with 10 nm of gold/palladium (80% gold, 20% palladium), using a BAI-TEC-SCD050 sputtering machine. Images were obtained with a Gemini-LEO-1550 scanning electron microscope (Carl Zeiss Jena, Germany), using a combination of the SE and Inlens detector set at 3 kV.

### Cell adhesion assay

For cell adhesion assay, individual wells of a TC-treated 24-well plate were surface functionalized by incubation with 5 μg/cm^2^ FN (from bovine plasma, Sigma Aldrich), 5 μg/cm^2^ Collagen-type-1 (rat tail, ibidi, Germany), 5 μg/cm^2^ Collagen-type-4 (human placenta, Merck), 0.2% Gelatine solution (porcine skin, Sigma Aldrich), and 0.01% solution Poly-L-Lysine (Sigma Aldrich). FN and Gelatine dilutions were prepared in PBS, while Collagen dilutions were prepared in 17.5 mM acetic acid. ECM proteins were incubated in wells for 1 h at 37°C. ECs were cultured as described above and harvested with Accutase (Sigma Aldrich) prior to seeding. Surfaces were washed once with PBS before 10,000 cells per well were seeded. Cells were allowed to adhere for 45 min at 37°C. Afterwards, plates were rinsed twice with PBS, and remaining cells were fixed with 4% PFA. Cell labelling with Vybrant DiO (Thermo Fisher Scientific) was performed according to manufacturer instructions.

### ECIS

Cell adhesion and spreading was quantified by recording capacitance at a frequency of 64 kHz, where the decrease in capacitance is directly proportional to the electrode coverage, using the ECIS Zθ (theta) instrument (Applied BioPhysics) [127]. ECs were harvested with Accutase (Sigma Aldrich) and pelleted by 4 min of 300 rpm centrifugation. Pellets were resuspended in basal M199 media. The amount of 5 × 10^4^ cells were seeded on 0.1% gelatine (EmbryoMax, Sigma Aldrich) coated 8W10E arrays (ibidi, Germany). To interfere with β1-integrin–ECM interactions, ECs were subjected to β1-integrin–blocking antibody (2.5 μg/ml, Abcam, P5D2 #ab24693) to isotype control IgG1 (2.5 μg/ml, Cell Signaling, #5415). Cells were kept in suspension and incubated with antibodies at 37°C by gentle inversion of the tube every 5 min. After 30 min, cells were seeded as described earlier. Influence of SMKI on cell adhesion and spreading was analyzed by culturing ECs for 3 d in M199 basal growth medium in the presence of K02288 (1 μM), SB-431542 (10 μM), or DMSO as control without medium change. Cells were harvested and seeded on arrays as described earlier.

### Collagen-lattice contraction assay

To prepare collagen type-1 lattices, 48-well T.C. plates were coated with FCS, 1 h prior to cell seeding, and left at the 37°C incubator before rinsing wells with PBS, in order to prevent lattice stickiness to the T.C. plastic. The following steps for lattice seeding were carried out with solutions prechilled on ice. Per lattice, 200 μl collagen (rat-tail, ibidi, Germany) solved in 17.5 mM acetic acid were neutralized by addition of 20 μl sterile neutralization buffer (100 mM carbonate/bicarbonate [*pH* 9.6]). Directly upon neutralization, collagen type 1 becomes insoluble and was rigorously vortexed. The amount of 100 μl of M199 media containing 2 mM L-glutamine, 100 units/ml penicillin, 10 μg/ml streptomycin, and 25 μg/ml Heparin was adjusted to neutral pH; 65 μL of FCS and 50 μg/mL EC growth supplement was added and again rigorously vortexed. A total of 500,000 cells/lattice were collected upon Accutase-based detachment by mild centrifugation at 300 rpm. Cell pellet was resuspended by adding 300 μl neutralized collagen-media mix and seeded into a 48-well plate prepared as written earlier. The plate was left at the incubator for 1 h before 500 μl of M199 basal growth media was added drop by drop at the corners of the well. For ROCK inhibition, 10 μM Y-27632 was added, while control cells were exposed to DMSO. The lattice diameter was documented using an optical scanning unit (Typhoon *FLA* 9500, GE Life Sciences), and cells were placed back to 37°C/5% CO_2_ atmosphere. The lattice diameter was documented again 24 h later.

### CFS

To test mechanical response to external deformation from single cells in cultured monolayers, CFS was used [128]. Silica colloids of 23 μm from Microparticles GmbH (Berlin, Germany) were glued with UHU-plus-Endfest, 2-component epoxy to the apex middle region of a tipless cantilever. Tipless cantilevers D from Bruker model MLCT-O10 were used, with a nominal spring constant of k = 0.03 N/m. Prior to an experiment, the system (cantilever + colloid) was calibrated by compressing a hard surface (mica) to obtain the cantilever sensitivity. Then, the thermal noise method was used to extract the spring constant of the cantilever. An AFM Nanoscope multimode 8 from Bruker was used in force spectroscopy mode for CFS experiments. All experiments were performed in a closed fluid chamber in M199 basal media with constant 37°C temperature using a thermal application controller (TAC). Cell monolayers grown on glass coverslips were rinsed with PBS and glued on round metal pucks with double-sided tape and were mounted on the AFM scanner where a fluid chamber was assembled. Approach-retraction cycles were taken with a constant velocity of 500 nm/s and a maximal loading force of 1 nN. To obtain the elasticity parameter or Young’s modulus E, the Nanoscope analysis software version 1.4 from Bruker was used to analyze all obtained force-distance curves. First, force-distance curves were converted to force-separation curves such that the distance of piezo displacement is transformed into distance between colloidal probe and cell surface. Then, a baseline correction to the cantilever for the free-of-contact region was made, and finally, the linearized version of the Hertz model was used to fit the experimental data. In this model, the applied force F is exerted by a spherical indenter on a soft deformable surface and is given as a function of the deformation δ as follows:

$${\left(F\right)}^{2/3}={\left(\frac{4}{3}\frac{E}{\left(1-\nu^{2}\right)}\sqrt{R}\right)}^{2/3}$$

where, in these experiments, R (radius of the spherical indenter) = 11,500 nm, ν (Poisson ratio for the cell surface) = 0.5 according to [129], δ is the deformation on the cell surface, and E is the Young’s modulus. Since the Hertz model is only valid within the range of small deformations (below approximately 10% the radius of the indenter), only the first 50 nm after contact between colloid and cell surface were considered to fit the data.

### QI by AFM

QI was performed using a NanoWizard 3 AFM (JPK Instruments, Berlin, Germany), mounted on an Olympus IX71 inverted phase-contrast microscope. Silicon cantilevers (qp-BioAC, Nanosensors, Switzerland) were used. Imaging was performed with the longest probe (CB3: 80 μm), possessing a nominal spring constant of 0.06 N/m. Prior to imaging, calibration was performed using the thermal noise method, after obtaining the deflection sensitivity of the cantilever by pressing the AFM tip against a hard reference substrate (glass). The actual spring constants ranged from 0.06 to 0.09 N/m.

All measurements were conducted on cell monolayers cultured on glass-bottom petri dishes. The cells were cultured for 48 h after seeding to reach the desired confluency. Prior to imaging, they were fixed in 1.25% glutaraldehyde solution for 2 min. During imaging, the samples were kept hydrated in PBS, and all measurements were performed at room temperature. Arrays of force-distance curves were automatically collected in QI mode with an applied load of 2 nN at a constant velocity of 50 μm/s. Data were collected on a selected grid size of 128 × 128 pixels, distributed over an area of 40 × 40 μm scan size. JPK SPM data processing software (JPK Instruments) was used for fitting each individual force-distance curve and for reconstructing the stiffness maps, which display the Young’s modulus of each individual pixel. For fitting, the retract segments of the collected force-distance curves were batch analyzed, using the Hertz/Sneddon fit function (conical indenter) and a Poisson ratio of 0.5.

### Use of micro-patterns

Micropatterned poly-L-lysine grafted polyethylene-glycol chips (CYTOO chip starter’s A x18) were obtained from CYTOO (Grenoble, France), and patterns were coated with FN^rho^ (20 μg/ml) (Cytoskeleton Inc., Denver, CO) according to manufacturer instructions. Chips were placed in 6-well plates, and 6 × 10^4^ cells were seeded on the chips in a volume of 4 ml basal M199 growth media. Cells were left to adhere, spread, and contract for 16 h before chips were rinsed in ice-cold PBS and fixed with 4% PFA for subsequent microscopy analysis.

### ECM deposition and decellularization

The following procedure was chosen after comparing different published decellularization strategies including different detergent-based decellularization protocols, which, in our hands, depleted LAP-TGFβ from FBN1 depots (evaluated by ICC). Instead, we used a snap freeze/thaw procedure followed by PBS/ddH_2_O/PBS washing (and sonication). For this, 50,000 cells/well of 12-well plates were seeded either directly on T.C. plastic (substrate for reporter-gene assay) or on uncoated heat-sterilized glass coverslips (immunofluorescence) in a volume of 2 ml basal M199 medium. Cells were left to produce ECM for 6–9 consecutive days without media exchange. After this, medium was removed and replaced by 1 ml ice-cold PBS before plates were frozen at −80°C. Frozen plates were left for at least 40 min before thawing at a 37°C water bath for 1 min. Immediately after, cells were placed on ice, and liquid was aspirated. Surfaces were then rinsed once with ice-cold deionized water (ddH_2_O) and then left in fresh ddH_2_O on ice for 2 min. Water was removed and replaced again by ice-cold PBS. After 1 min, the detachment of remaining cells was monitored by phase-contrast microscopy. For TGFβ bioassay, in case there were still fewer cells remaining, 12-well plates were placed into a Sonorex sonication bath (Bandelin, Germany) and sonicated for 10 s followed by an additional PBS/ddH_2_O/PBS washing step. Samples were either fixed in 4% PFA or prepared for further downstream processing (see “TGFβ bioassay using dual luciferase reporter cells on decellularized substrates”).

### TGFβ bioassay using dual luciferase reporter cells on decellularized substrates

For TGFβ bioassay, HEK293T cells were transfected with CAGA_12_-dual luciferase reporter construct or additionally cotransfected with β1-integrin expression construct. Eighteen hours after transfection, cells were serum starved for 6 h. Then, reporter cells were harvested by Accutase-based detachment, centrifuged at 300 rpm for 2 min, and resuspended in DMEM supplemented with 0.5% FCS, 2 mM L-glutamine, 100 units/ml penicillin, and 10 μg/ml streptomycin to gain a concentration of 0.5 × 10^5^ cells/ml. For ROCK inhibition, 20 μM Y-27632 was added into the cell suspension, while control cells were exposed to DMSO. The amount of 1 ml of reporter-cell suspension was seeded on decellularized substrates and left to incubate at 37°C/5% CO_2_ atmosphere for an additional 24 h. Media was aspirated, and cells were rinsed carefully in ice-cold PBS and lysed in 1× passive lysis buffer (Promega). Measurements were carried out as previously described [130] using the InfiniteProF200 luminescence plate reader (Tecan, Maennedorf, Switzerland).

### Measurement of latent versus active TGFβ from conditioned cell culture supernatants

For detection of TGFβ from conditioned media of BMPR2-deficient cells, we used an HEK293T CAGA_12_-reporter assay. For this, 50,000 control cells or BMPR2-deficient cells were grown in 12-well plates and grown for 6–9 d. After this, conditioned media was harvested and stored immediately at −80°C until assaying. For this, detached cells were removed by brief centrifugation at 500 rpm for 5 min, and supernatant was divided into 2 fractions of 350 μl each. For activation of latent TGFβ, 350 μl of conditioned media was mixed with 70 μl 1 N HCL and incubated for 10 min at room temperature. After this, acidified supernatants were neutralized by mixing with 270 μl of 1.2 N NaOH/0.5M HEPES. Successful neutralization was titrated before. For nonacidified controls, 350 μl of conditioned supernatants was mixed with 350 μl PBS. The amount of 50 μl of neutralized supernatants was then used immediately to stimulate confluent HEK293T CAGA_12_-reporter cells seeded in 96 wells. For this, reporter cells were starved for 6 h in 50 μl DMEM without FCS and supplemented with 50 μl of supernatants for an additional 24 h before luciferase measurements were performed. All firefly luciferase measurements were normalized against renilla luciferase values from the same samples to give relative light units (RLUs). For representation of active TGFβ in supernatants of BMPR2-deficient cells compared to control cells, the fold induction of acidified samples relative to nonacidified samples is shown.

### Image acquisition and analysis

Structural representation of proteins was performed using PyMOL 2.1 (Schrödinger). Confocal raw data were post-processed and adjusted for color and contrast (linear adjustments maintained for confocal data sets represented within one figure) using LAS-X software including DyeFinder and 3D visualization. Epifluorescence images were adjusted for color and contrast using the “linear BestFit” function in AxioVision (Zeiss) software. Microscopy images were inverted and pseudo-colored in Adobe Photoshop 7 software. Line scans were performed in ImageJ with stacked images, followed by the “plot profile” function of a line, covering the region of interest. Intensity values were normalized to a scale of 0%–100% and plotted in GraphPad Prism 7 (GraphPad Software Inc.). Binary images of single confocal z-planes of decellularized FBN1 staining were generated with the the ImageJ plugin FibrilJ [131]. Mean signal intensity of luminal FBN1 deposits in PAs were quantified using the circle tool in ImageJ in regions beyond the border of the inner elastic membrane. IHC sections of different donors were all stained at the same time, and images were acquired with the same exposure time. At least 10 different PAs originating from up to 3 different donors were compared (control *n* = 3, IPAH *n* = 3, HPAH *n* = 1). Schemes and cartoon drawings were prepared using Corel Draw Graphics Suite X8.

### RNA-Seq

#### RNA-Seq library preparation and sequencing

For the analysis of differential gene expression, WT and BMPR2-mutant cells were grown as confluent monolayer in full medium conditions for 3 d. After lysis, the NucleoSpin RNA from MACHEREY-NAGEL was used according to manufacturer instructions to isolate RNA. After initial quality control using Agilent’s Bioanalyzer, sequencing libraries were prepared from 500 ng of total RNA per sample following Roche’s stranded “KAPA RNA HyperPrep” library preparation protocol for single indexed Illumina libraries: First, the polyA-RNA fraction was enriched using oligo-dT–probed paramagnetic beads. Enriched RNA was heat-fragmented and subjected to first strand synthesis using random priming. The second strand was synthesized incorporating dUTP instead of dTTP to preserve strand information. After A-tailing Illumina sequencing, compatible adapters were ligated. Following bead-based clean-up steps, the libraries were amplified using 10 cycles of PCR. Library quality and size was checked with qBit, Agilent Bioanalyzer, and qPCR. Sequencing was carried out in biological triplicates on an Illumina HiSeq 4000 system in SR75bp mode (single read, 75 bp read length) yielding between 46 and 69 million fragments per sample.

### RNA-Seq data analysis

#### Processing of RNA-Seq experiments

Single-end, 75-bp reads from Illumina sequencing were mapped to the reference genome (hg19) using the STAR mapper (splice junctions based on RefSeq; options: ‐‐alignIntronMin20 ‐‐alignIntronMax500000 ‐‐outFilterMismatchNmax 10). Differential gene expression was ascertained using the DESeq2 package [132]. The cut-off for significantly altered gene expression was a fold change of <0.5 or >2 with an adjusted *P* value of 0.05. The shared differentially expressed genes in both BMPR2-deficient EC lines were identified with BioVenn [133]. For heatmaps, z-score of these genes were used and hierarchically clustered using the “pheatmap” package in RStudio. Functional annotation clustering was performed using DAVID Bioinformatics Resources 6.8 [134,135]. Functional clusters with Benjamini-corrected *P* < 0.05 were considered significant.

### ChIP-Seq promoter analysis

Publicly available data sets deposited in the Gene Expression Omnibus (GEO) database at the National Center for Biotechnology Information (http://www.ncbi.nlm.nih.gov/geo), under accession number GSM684747 and GSM2429820, were used to display SMAD1/5 ChIP-Seq track of HUVECs treated with BMP9 [53] and pSMAD1/5 ChIP-Seq track of MDA-MB-231 cells treated with TGFβ1 [41] in the Integrative Genomics Viewer [136].

### ChIP

For ChIP, the simpleCHIP enzymatic ChIP-Kit with magnetic bead separation was chosen and performed according to manufacturer instructions. For one immunoprecipitation, 3.5 × 10^6^ cells were seeded in basal growth medium into a 16-cm dish and left untreated for 3 consecutive days, before PFA fixation and chromatin isolation was performed. Before, optimal chromatin digestion conditions were identified following APPENDIX-A of the manufacturer instructions revealing 7.5 μl of 1:10 endonuclease dilution as the optimal concentration for efficient chromatin digestion per immunoprecipitation. The amount of 100 μl input of crosslinked and digested chromatin (corresponds to approximately 10 μg) was diluted in 1× ChIP buffer and proceeded for immunoprecipitation using 10 μg Smad1XP antibody (CellSignalling) or 10 μg of nonspecific IgG control in 1 ml overnight. After elution of immunoprecipitated DNA from magnetic beads and reverse of crosslink, DNA was purified via spin columns and eluted in 50 μl of DNA elution buffer.

### qRT-PCR

RNA was purified using NucleoSpin RNA II (Machery-Nagel) according to manufacturer instructions. The amount of 1 μg RNA was reverse transcribed into cDNA (M-MLV reverse transcriptase, Promega). qRT-PCR was performed using SYBR Green Master Mix, StepOne Plus, and StepOne Software 2.3 (Applied Biosystems). Target gene expression was quantified relative to RSP9 using the ΔΔCT method including primer efficiency [137]. Quantification of ChIPs was performed with 0.5 μL eluate DNA, primers directed against SMAD1/5 occupied regions in the ITGB1 and ID3 promoter and SYBR Green Master Mix. ChIP results were calculated using fold enrichment relative to IgG samples of the respective cell line. Measurements were done in technical triplicates. All primers are listed in S1 Table.

### Statistical analysis

Statistical analysis for densitometric protein level quantification, qRT-PCR, RPKM values, quantitative image analysis, ECIS, CAGA Dual luciferase assay, and CFS were performed using GraphPad Prism 7 (GraphPad Software Inc.). Normal distribution of data sets *n* < 5 were tested with the Shapiro-Wilk normality test. Data sets *n* ≥ 5 were tested additionally with the Kolmogorov Smirnov test for normality. In cases of failure to reject the null hypothesis, the ANOVA and Bonferroni post hoc test were used to check for statistical significance under the normality assumption. Upon rejection of the null hypothesis (e.g., all densitometric protein level quantifications displayed as fold induction), the Kruskal-Wallis test and a post hoc Dunn’s multiple-comparisons test or a two-tailed Mann-Whitney test were applied. Significance levels were assed for differences between BMPR2^wt^ and BMPR2- deficient cell clones. For all experiments, statistical significance was assigned with an alpha level of *P* < 0.05 (*P < 0.05, **P < 0.01, ***P < 0.001, ****P < 0.0001).

### Illustrations

Graphical illustrations were created with CorelDRAW X8 and BioRender (https://biorender.com).

## Supporting information

S1 Fig Generation and validation of BMPR2^ΔE2^ and BMPR2^KO^ mutant ECs carrying BMPR2 mutations leading to BMPR2 deficiency. (A) Targeting strategy to produce and detect *BMPR2*-deletion clones by CRISPR directed cleavage in *BMPR2* exon 2 and schematic diagram of established BMPR2-deficient EC cell lines. *BMPR2*^*ΔE2*^ harboring a 165-nt deletion of exon 2 with concomitant loss of exon 2 splice acceptor site causing exon skipping from the final transcript and truncated BMPR2 protein expression. Single *BMPR2* copy deletion (*BMPR2*^*KO*^) was achieved by a 53-nt frameshift deletion which results in a premature termination codon (PTC) and non-sense–mediated mRNA decay. Black arrows indicate primers to validate exon 2 deletions. (B) cDNA Sanger sequencing at the target site for 3 different *BMPR2* cell clones. Cas9 cutting site is indicated by arrowhead. Relative location to the PAM sequence is indicated. (C) Structure of BMP2 homo-dimer (blue) (PDB 2H64) superimposed to the crystal structure of the extracellular domain of BMPR2 (PDB 2HLQ). Deletion of exon 2 (aa26–aa83) results in a truncated receptor lacking critical interfaces for ligand binding (red) and loss of 2 extracellular disulfide bonds (yellow) important for protein folding. (D) qRT-PCR data on *BMPR2*^*WT*^ transcript (blue) relative to *BMPR2*^*ΔE2*^ *transcript* levels *(red)* and loss of *BMPR2* expression (white). Values are expressed as relative mean (*n* = 3). Statistics are not shown due to clarity. (E) Immunoblot and densitometric quantification from total cell extracts of indicated cell clones using an antibody specific to BMPR2, binding to a carboxy-terminal epitope preserved in both *BMPR2*^*wt*^ *and BMPR2*^*ΔE2*^ (predicted molecular weight BMPR2^wt^ approximately 140–150 kDa; BMPR2^*ΔE2*^ approximately 130 kDa) (left). Data are presented as mean + SD relative to lane 1 (one-way ANOVA with post hoc Bonferroni, *n* = 4 independent experiments). (F) Cell surface biotinylation at primary amines followed by precipitation using Streptavidin in indicated clones (upper) or Cos7 cells overexpressing indicated BMPR2 constructs (lower). (G) Confocal microscopy of *BMPR2*^*ΔE2*^ cells transiently transfected with a myc-tagged BMPR2ΔE2 construct. Cells were immunostained with anti-BMPR2 antibody (green) and anti-myc antibody (red); see S1 Data for underlying data. ****P < 0.0001; scale bars, 10 μm. nt, nucleotide; PAM, protospacer adjacent motif.
(TIF)

S2 Fig Characterization of altered Activin signaling in BMPR2-deficient ECs. (A) BMPR2-deficient ECs confer sensitivity to Activin A. Dose response (1.5, 3, 10 nM) of Activin A–dependent phosphorylation of SMAD1/5 and SMAD2 upon 15 min of stimulation. si, small interfering
(TIF)

S3 Fig BMPR2-deficient ECs signal through hetero-oligomers comprising BMP and TGFβ receptors as indicated by the formation of mixed SMAD complexes. (A) Immunoblot demonstrating efficiency of TβR2 knock-down by siRNA (20 nM). (B) The ALK5 selective inhibitor SB-431542 abolishes BMP6-SMAD2 but not SMAD1/5 phosphorylation (upper), while the ALK2 selective inhibitor K02288 abolishes BMP6-SMAD1/5 phosphorylation (lower). (C) Epifluorescence images of PLA (left) showing complexes of SMAD5 (S5) with SMAD2/3 (S2/3) in indicated cell clones upon TGFβ stimulation (200 pM) for 15 min. PLA signals are pseudo-colored greyscale and inverted (upper). Scale bar, 10 μm. (D) Quantification of SMAD5-SMAD2/3 PLA signals (right) in TGFβ-stimulated cells with the number of nuclear, cytosolic, and overall PLA foci shown. Data are presented as mean ± SD (*n* ≥ 7 frames, 20–30 cells each). See S2 Data for underlying data. (E) PLA controls for *BMPR2*^*ΔE2*^ mutant ECs shown in panel C, i.e., SMAD5 and SMAD2/3 antibodies alone (upper) or for PLA shown in Fig2E, i.e., SMAD1, SMAD2 antibodies alone (lower). (F) PLA positive control: 15 min TGFβ (200 pM) stimulation for SMAD2/3-co-SMAD4 complexes in *BMPR2*^*KO*^ cells. Statistical significance relative to BMPR2^wt^ was calculated using one-way ANOVA and Bonferroni post hoc test for PLA data; *P < 0.05, **P < 0.01, ***P < 0.001, ****P < 0.0001. n.s., not significant
(TIF)

S4 Fig Differential expression of TGFβ pathway members and increased SMAD1 occupancy at ID3 promoter. (A, B) RNA-Seq analysis of WT and BMPR2-deficient ECs under steady-state conditions (*n* = 3 independent replicates). (A) Hierarchical clustering of differentially expressed TGFβ pathway members. Heatmap color coding shows z-score of differentially regulated genes (red = high; blue = low). (B) Relative expression of ligands, TGFβ, and BMP type-1, type-2 and co-receptors under steady-state conditions shown with RPKM values. Note that ALK1 and ENG are both significantly reduced in BMPR2-deficient ECs. (C) Verification of increased ITGB1 expression in BMPR2-deficient ECs by qRT-PCR analysis (*n* = 6). (D) IGV browser displays over the *ID3* loci showing SMAD1/5 ChIP-Seq track of HUVECs treated with BMP9 [53] and pSMAD1/5 ChIP-Seq track of MDA-MB-231 cells treated with TGFβ1 [41]. ChIP-Seq data were retrieved from the GEO (GSM684747, GSM2429820). (E) SMAD1 occupancy at the ID3 promoter was validated by ChIP-qPCR in steady-state conditions. IPs are a representative experiment of two, and ChIP-qPCR was performed in triplicates shown with means + SD. (F) Verification of altered *ID1*, *ID2*, and *ID3* expression in BMPR2-deficient ECs by qRT-PCR analysis (*n* ≥ 4). Statistical significance relative to BMPR2^wt^ was calculated for RPKM values using one-way ANOVA and Bonferroni post hoc test and for qRT-PCR data using the Kruskal-Wallis test with post hoc Dunn test; *P < 0.05, **P < 0.01, ***P < 0.001, ****P < 0.001. See S3 Data for underlying data. n.s., not significant
(TIF)

S5 Fig EndMT and alterations in F-actin organization induce subcellular stiffening. (A) Maximum projection of confocal z-stacks showing cell junctions of indicated cell clones immuno-labelled with an anti-N-Cadherin (green) antibody. (B) Single confocal z-planes (medial) showing cell junctions of indicated cell clones immuno-labelled with an anti-β-catenin (red) antibody. Scale bars, 10 μm. (C) SEM micrographs of indicated cell clones, showing different organization of CCC sites between 2–3 neighboring cells (indicated). Figure enlargements with higher resolution (below) are indicated by white frame. (D) qRT-PCR of indicated cell clones for EndMT transition markers *SNAIL* and *TWIST* under steady-state growth conditions. Values are expressed as mean F.I. relative to *BMPR2*^*ΔE2*^ + SD (*n* = 3 independent experiments). See S4 Data for underlying data. (E) Representative single confocal z-planes showing the distribution of pMLC (red) and ILK (green) of indicated cell clones. Scale bars, 10 μm. (F) Maximum projections of basal-to-apical confocal z-planes of indicated ECs stained with Phalloidin (white pseudo-color). See S1 Movie. Figure enlargements are indicated by yellow frame. F.I., fold induction; ILK, integrin-linked kinase; n.d., not detected; SEM, scanning electron microscopy
(TIF)

S6 Fig BMPR2-deficient ECs spread on RGD-containing ECM. Cell adhesion on dishes (TC plastic) coated with ECM proteins (all 5 μg/cm^2^). Representative pictures of cells, which were seeded on TC plastic, FN, or collagen I and counterstained using DiO (pseudo-color white) labelling and DAPI. Spreading area is quantified in Fig 5B (*n* = 3 independent experiments). Scale bars, 50 μm. TC, tissue culture.
(TIF)

S7 Fig Concomitant localization of mechanical components of the matrix, the cell membrane, and cytoskeleton from basal to EC junctions in BMPR2-deficient ECs. (A) Maximum projection of confocal z-stacks immunostained for ILK (green) and FBN1 (red) for indicated cell clones (upper). Side-view projection of single confocal z-plane indicated by white line in upper. Relative localization of ILK (green) and FBN1 (red) is indicated by white arrowhead. See also S3 Movie. (B) Single confocal z-planes (medial) immunostained for ITGB1 (green) and FBN1 (red) for indicated cell clones (upper). Side projection of confocal plane indicated by white line in upper. Relative localization of ITGB1 (green) and FBN1 (red) is indicated by white arrowhead. See also S4 Movie. (C) Maximum projection of confocal z-stacks immunostained for endogenous Fn (red) and the F-actin cytoskeleton (green) of indicated cell types (left). Figure enlargement depicting relative localization of Fn fibers (red) and bundles of filamentous actin (green) (right; white arrowhead). (D) Maximum projection of confocal z-stacks immunostained for FBN1 (red) and F-actin (green) of indicated cell clones (left). Figure enlargement depicting relative localization of FBN1 fibers (red) and bundles of filamentous actin (green) (right; white arrowhead). Line scans (blue line, upper) of single confocal z-planes (indicated) showing maximum signal intensity in percent (lower). (E) Characterization of decellularized deposits of FBN1 (binarized images of single confocal z-planes) from BMPR2^wt^ or BMPR2-deficient cells (upper). Scale bars represent 10 μm.
(TIF)

S8 Fig Ectopic LTPB-1 deposits surround αSMA-positive cells in inner luminal PAs from IPAH and HPAH donors with low BMPR2 expression. (A–B) Representative PAs of control, IPAH, and HPAH donors were stained for LTBP-1 or αSMA (red), collagen, and elastin at approximately 520 nm emission (green) and DAPI (blue) (i). (A) Higher magnification of the area surrounding the iem (ii) shows co-localization of LTBP-1 with the elastic membrane in the tm and sub-EC layer (e.g., basal lamina) in controls, while LTBP-1 staining similar to FBN1 (Fig 7D) exceeds the iem toward the lumen of PAs from IPAH (middle) and HPAH (right) donors. (B) αSMA staining (red) was restricted to the tm in controls, while IPAH/HPAH PAs αSMA stained additional the intima and lumen. Scale bar represents 50 μm. lu, lumen; tm, tunica media.
(TIF)

S9 Fig High IL-6 expression and LTBP-1 deposition detected in BMPR2-deficient cells. (A) Immunocytochemical stainings against LTBP-1 by indicated cell clones. Note the junctional accumulation of LTBP-1 puncta in BMPR2-deficient cells. Region of interest zoom-in are shown in rectangular boxes. (B) qRT-PCR under steady-state culture conditions for indicated cell clones. Scale bars represent 20 μm. Values are expressed as fold induction (*n* = 3 independent experiments). Data are shown as mean + SD relative to BMPR2^wt^. See S4 Data for underlying data. F.I., fold induction
(TIF)

S1 Movie 3D reconstruction of confocal z-stacks (basal to apical) of BMPR2^wt^ (left) BMPR2^ΔE2^ (middle) BMPR2^KO^ (right) cells cultured under steady-state growth conditions with filamentous actin cytoskeleton (F-actin) shown in red (Phalloidin) and the nucleus in blue (DAPI) (first sequence of movie). Second movie sequence represented as color-coded topographical view with basal structures indicated in blue, medial structures indicated in green/yellow, and apical structures indicated in red.
(MP4)

S2 Movie Remodeling of FN^rho^ (red) by *BMPR2*^*wt*^ (left) and *BMPR2*^*ΔE2*^ (right) ECs with cell bodies shown by phase contrast microscopy. Cells were cultured for 6 h in the presence of 20 μg/ml Fn^rho^, and region of interest was chosen for subsequent video imaging. Video imaging was performed for 6 h with 5-min time frames between individual pictures. Scale bar, 10 μm.
(MP4)

S3 Movie 3D reconstruction of confocal z-stacks (basal to apical) of *BMPR2*^*wt*^ (left) and *BMPR2*^*ΔE2*^ (right) cells cultured under steady-state growth conditions and immunostained for ILK (green) (first sequence of movie) and FBN1 (red) (second sequence of movie) and the nucleus in blue (DAPI). Scale bars are indicated.
(MP4)

S4 Movie 3D reconstruction of confocal z-stacks (basal to apical) of *BMPR2*^*wt*^ (left) and *BMPR2*^*ΔE2*^ (right) cells cultured under steady-state growth conditions and immunostained for β1-integrin (green) (first sequence of movie) and FBN1 (red) (second sequence of movie) and the cell’s nucleus in blue (DAPI). Scale bars are indicated.
(MP4)

S1 Data Data underlying Fig 1B, 1C, 1E, 1F and S1D and S1E Fig. (XLSX)

S2 Data Data underlying Fig 2D and 2E and S3D Fig. (XLSX)

S3 Data Data underlying Fig 3B, 3C, 3D, 3E, 3G and S4A, S4B, S4C, S4F Fig. (XLSX)

S4 Data Data underlying Fig 4A and 4G, S5D and S9 Figs. (XLSX)

S5 Data Data underlying Fig 5A, 5B, 5C, 5D and 5E. (XLSX)

S6 Data Data underlying Fig 6D. (XLSX)

S7 Data Data underlying Fig 7A and 7C. (XLSX)

S8 Data Data underlying Fig 8F and 8H. (XLSX)

S1 Raw Images Raw images of WB shown in Figs 1B, 1C, 1D, 1E, 2B, 2C, 3A, 4A, 4D, 4F, 6B, 8B and S1E, S1F, S2A Figs. (PDF)

S1 Table List of primers used in this study. (XLSX)

## Abbreviations

5f: form follows function follows form
αSMA: alpha smooth muscle actin
ACVR2A: Activin A receptor, type-2 A
ACVR2B: Activin A receptor, type-2 B
AFM: atomic force microscopy
AJ: adherens junction
ALK: Activin receptor-like kinase
BMP: bone morphogenetic protein
BMPR2: BMP type-2 receptor
BSA: bovine serum albumin
CCC: cell-to-cell contact
CCM: cerebral cavernous malformation
CFS: colloidal force spectroscopy
ChIP-Seq: chromatin immunoprecipitation sequencing
CMC: cell-to-ECM contact
DAPI: 4′,6-diamidino-2-phenylindole
EBNA: Epstein-Barr virus nuclear antigen
EC: endothelial cell
ECIS: electric cell-substrate impedance sensing
ECM: extracellular matrix
EEM: external elastic membrane
EMT: epithelial-to-mesenchymal transition
EndMT: endothelial-to-mesenchymal transition
ENG: Endoglin
FA: focal adhesion
FBN1: fibrillin-1
FCS: fetal calf serum
FFF: form follows function
FN: fibronectin
FNrho: rhodamine-labelled FN
FOP: fibrodysplasia ossificans progressive
GEO: Gene Expression Omnibus
GO: Gene Ontology
GSEA: Gene Set Enrichment Analysis
HHT: hereditary hemorrhagic telangiectasia
HPAH: heritable PAH
HUVEC: human umbilical vein endothelial cell
ICC: immunocytochemistry
IgG: immunoglobulin G
IHC: immunohistochemistry
IL-6: interleukin-6
ILK: integrin-linked kinase
IPAH: idiopathic PAH
ITGB1: Integrin subunit beta 1
LAP: latency-associated peptide
LLC: large latency complex
LTBP1: latent transforming growth factor beta-binding protein 1
MLC: myosin light chain
NMD: non-sense-mediated decay
PAH: pulmonary arterial hypertension
PBS: phosphate buffered saline
PEI: polyethylenimine
PFA: paraformaldehyde
PI3K: phosphoinositide 3-kinase
PLA: proximity ligation assay
pMLC: phosphorylated MLC
QI: quantitative imaging
qPCR: quantitative PCR
qRT-PCR: quantitative real-time PCR
R-SMAD: Receptor-regulated SMAD
RLU: relative light unit
RNA-Seq: RNA sequencing
ROCK: Rho-associated protein kinase
RPKM: Reads per kilo base per million mapped reads
Ser785: Serine 785
siRNA: small interfering RNA
SLC: small latency complex
SMAD: Suppressor of Mothers against Decapentaplegic
SMC: smooth muscle cell
SMKI: small-molecule kinase inhibitor
TβR2: TGFβ type-2 receptor
TAC: thermal application controller
TC: tissue culture
TGFβ: transforming growth factor-beta
tm: tunica media
TNC: tenascin-C
TSS: transcription start site
Tyr783: Tyrosine 783
VE: vascular endothelial
WB: western blotting
WT: wild type
