# Supplementary figures and images for: BMPR2 acts as a gatekeeper to protect endothelial cells from increased TGFβ responses and altered cell mechanics
Source: PLoS Biol. 2019 Dec 11;17(12):e3000557. doi: 10.1371/journal.pbio.3000557 (PMC6927666; doi:10.1371/journal.pbio.3000557)

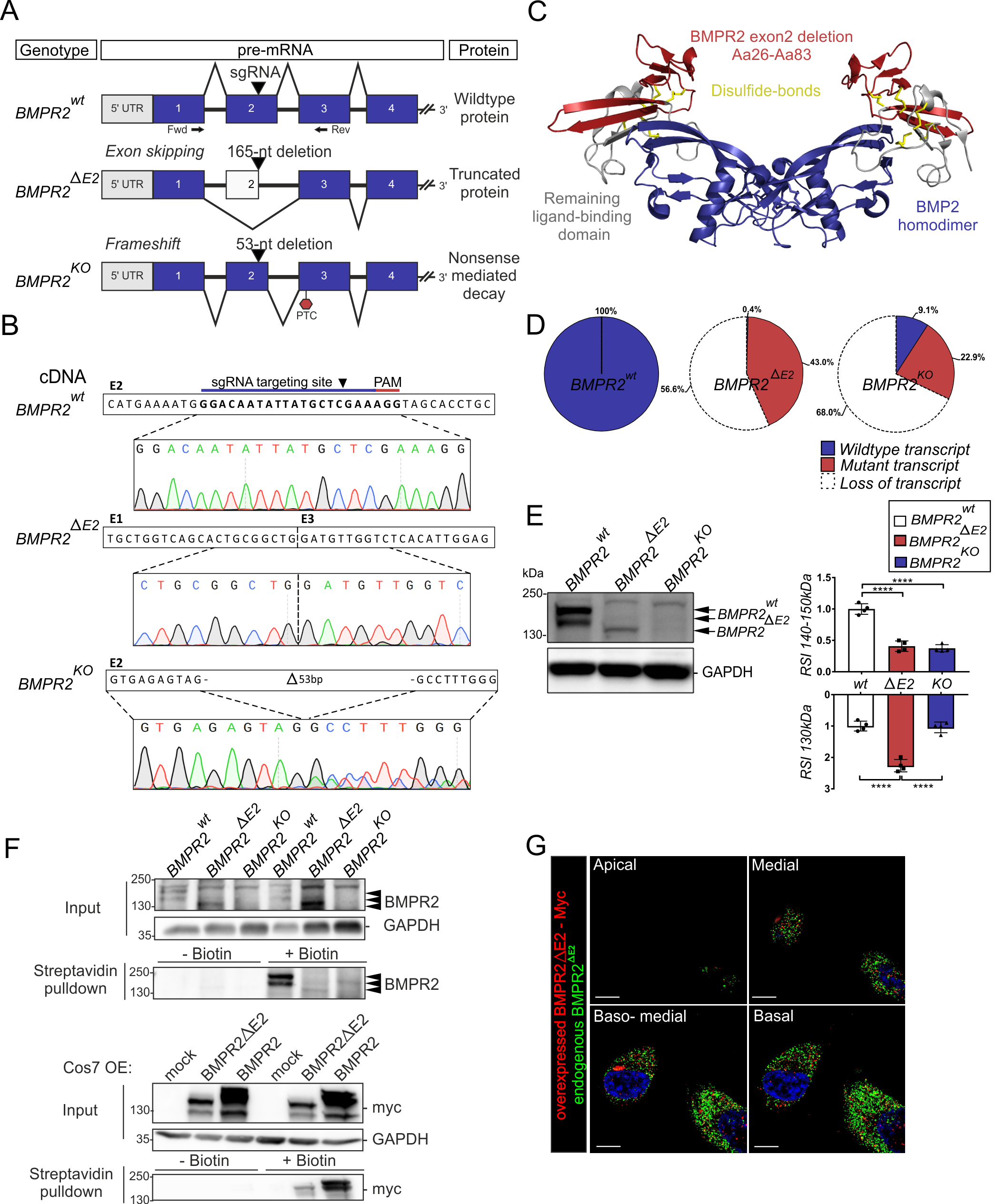

Supplement: S1 Fig — (A) Targeting strategy to produce and detect BMPR2-deletion clones by CRISPR directed cleavage in BMPR2 exon 2 and schematic diagram of established BMPR2-deficient EC cell lines. BMPR2ΔE2 harboring a 165-nt deletion of exon 2 with concomitant loss of exon 2 splice acceptor site causing exon skipping from the final transcript and truncated BMPR2 protein expression. Single BMPR2 copy deletion (BMPR2KO) was achieved by a 53-nt frameshift deletion which results in a premature termination codon (PTC) and non-sense–mediated mRNA decay. Black arrows indicate primers to validate exon 2 deletions. (B) cDNA Sanger sequencing at the target site for 3 different BMPR2 cell clones. Cas9 cutting site is indicated by arrowhead. Relative location to the PAM sequence is indicated. (C) Structure of BMP2 homo-dimer (blue) (PDB 2H64) superimposed to the crystal structure of the extracellular domain of BMPR2 (PDB 2HLQ). Deletion of exon 2 (aa26–aa83) results in a truncated receptor lacking critical interfaces for ligand binding (red) and loss of 2 extracellular disulfide bonds (yellow) important for protein folding. (D) qRT-PCR data on BMPR2WT transcript (blue) relative to BMPR2ΔE2 transcript levels (red) and loss of BMPR2 expression (white). Values are expressed as relative mean (n = 3). Statistics are not shown due to clarity. (E) Immunoblot and densitometric quantification from total cell extracts of indicated cell clones using an antibody specific to BMPR2, binding to a carboxy-terminal epitope preserved in both BMPR2wt and BMPR2ΔE2 (predicted molecular weight BMPR2wt approximately 140–150 kDa; BMPR2ΔE2 approximately 130 kDa) (left). Data are presented as mean + SD relative to lane 1 (one-way ANOVA with post hoc Bonferroni, n = 4 independent experiments). (F) Cell surface biotinylation at primary amines followed by precipitation using Streptavidin in indicated clones (upper) or Cos7 cells overexpressing indicated BMPR2 constructs (lower). (G) Confocal microscopy of BMPR2ΔE2 cells tra [file pbio.3000557.s001.tif]

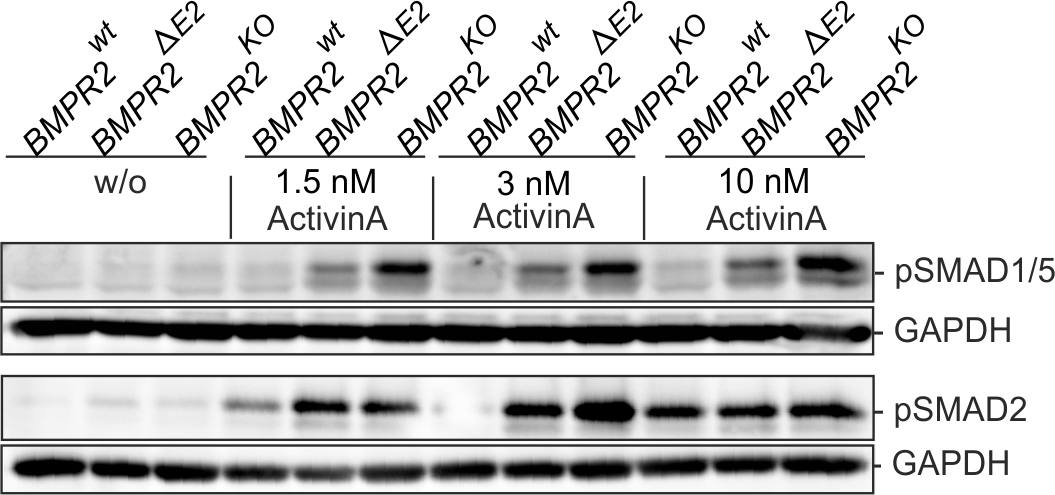

Supplement: S2 Fig — (A) BMPR2-deficient ECs confer sensitivity to Activin A. Dose response (1.5, 3, 10 nM) of Activin A–dependent phosphorylation of SMAD1/5 and SMAD2 upon 15 min of stimulation. si, small interfering (TIF) [file pbio.3000557.s002.tif]

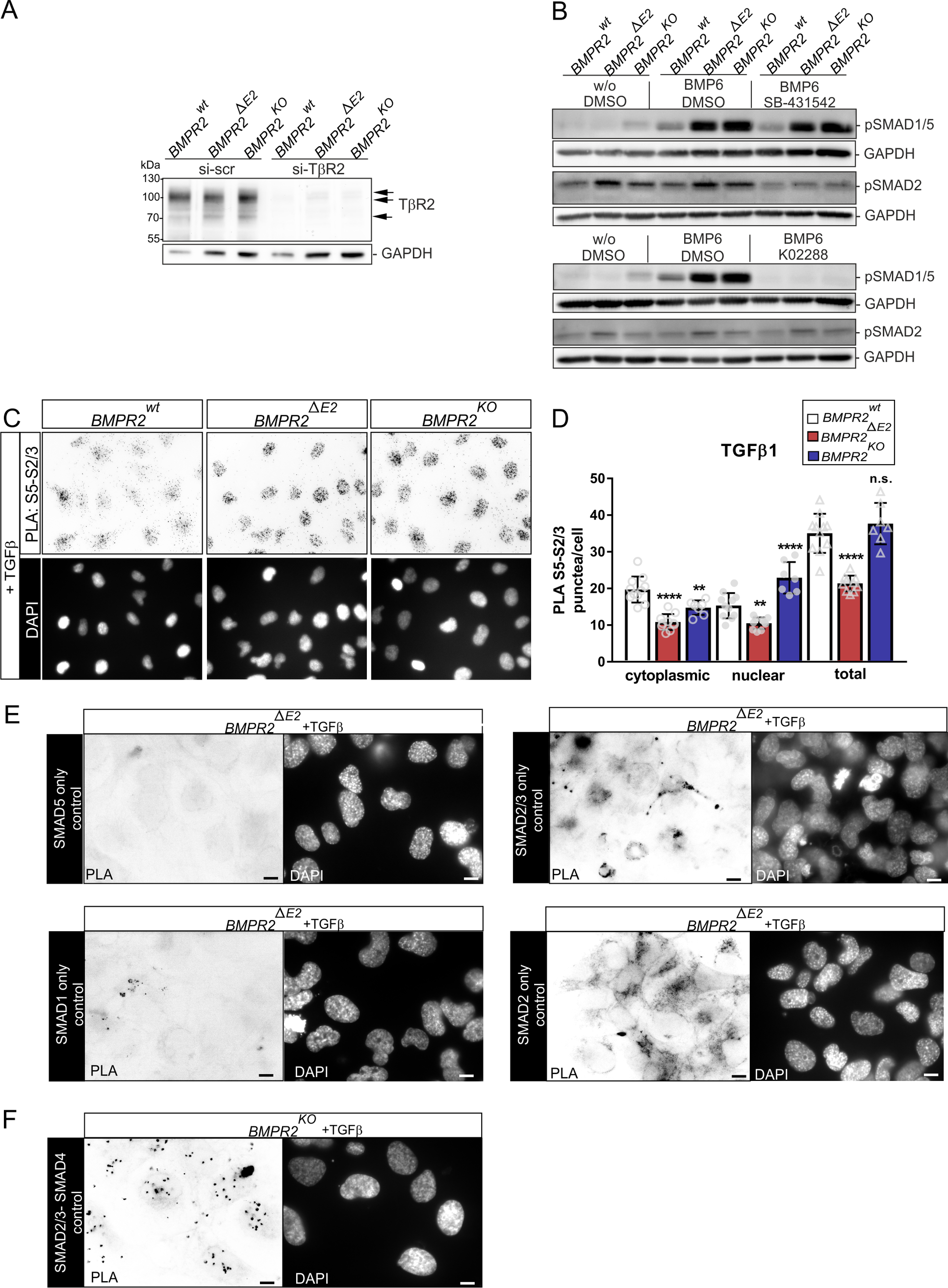

Supplement: S3 Fig — (A) Immunoblot demonstrating efficiency of TβR2 knock-down by siRNA (20 nM). (B) The ALK5 selective inhibitor SB-431542 abolishes BMP6-SMAD2 but not SMAD1/5 phosphorylation (upper), while the ALK2 selective inhibitor K02288 abolishes BMP6-SMAD1/5 phosphorylation (lower). (C) Epifluorescence images of PLA (left) showing complexes of SMAD5 (S5) with SMAD2/3 (S2/3) in indicated cell clones upon TGFβ stimulation (200 pM) for 15 min. PLA signals are pseudo-colored greyscale and inverted (upper). Scale bar, 10 μm. (D) Quantification of SMAD5-SMAD2/3 PLA signals (right) in TGFβ-stimulated cells with the number of nuclear, cytosolic, and overall PLA foci shown. Data are presented as mean ± SD (n ≥ 7 frames, 20–30 cells each). See S2 Data for underlying data. (E) PLA controls for BMPR2ΔE2 mutant ECs shown in panel C, i.e., SMAD5 and SMAD2/3 antibodies alone (upper) or for PLA shown in Fig2E, i.e., SMAD1, SMAD2 antibodies alone (lower). (F) PLA positive control: 15 min TGFβ (200 pM) stimulation for SMAD2/3-co-SMAD4 complexes in BMPR2KO cells. Statistical significance relative to BMPR2wt was calculated using one-way ANOVA and Bonferroni post hoc test for PLA data; *P < 0.05, **P < 0.01, ***P < 0.001, ****P < 0.0001. n.s., not significant (TIF) [file pbio.3000557.s003.tif]

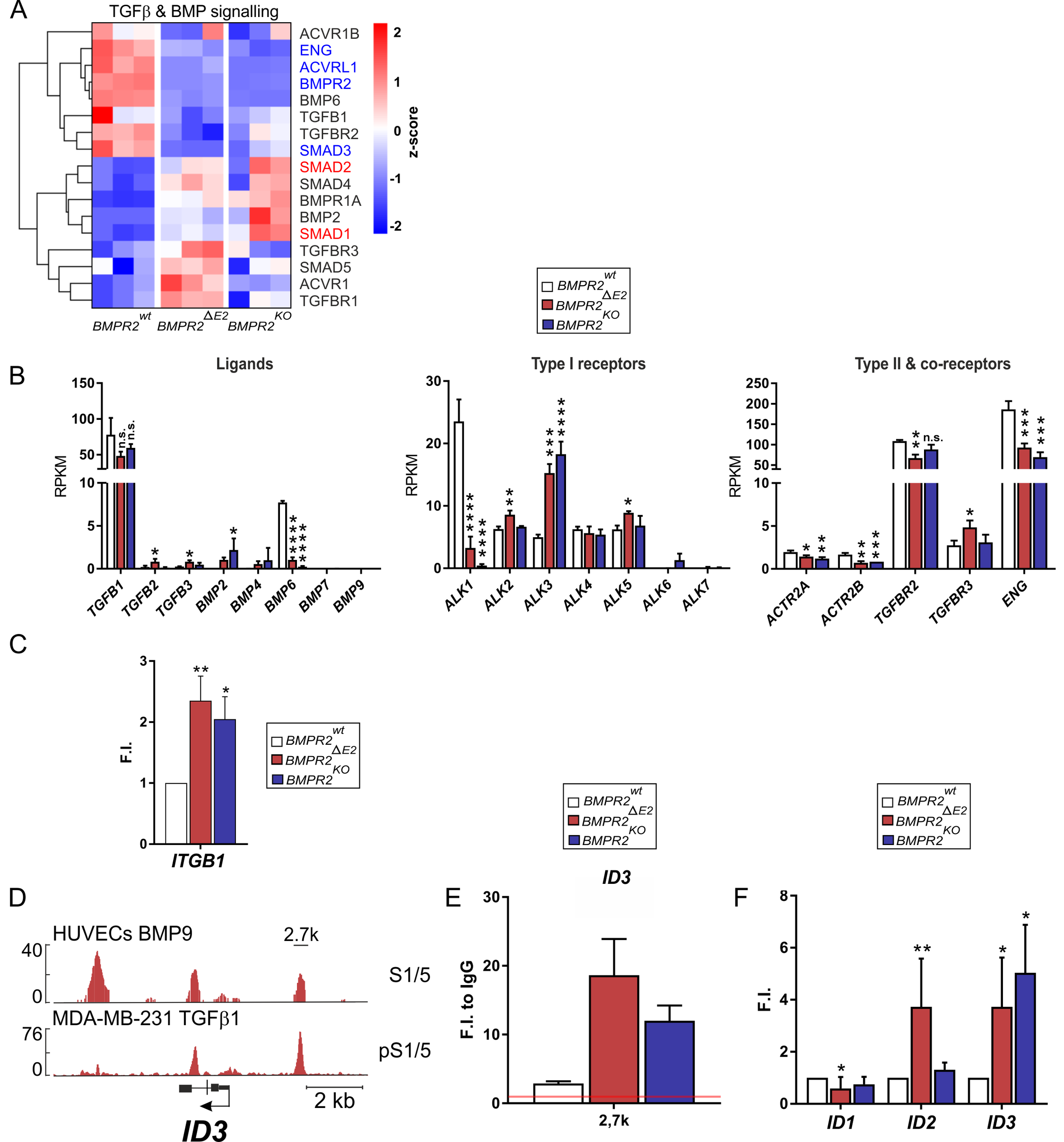

Supplement: S4 Fig — (A, B) RNA-Seq analysis of WT and BMPR2-deficient ECs under steady-state conditions (n = 3 independent replicates). (A) Hierarchical clustering of differentially expressed TGFβ pathway members. Heatmap color coding shows z-score of differentially regulated genes (red = high; blue = low). (B) Relative expression of ligands, TGFβ, and BMP type-1, type-2 and co-receptors under steady-state conditions shown with RPKM values. Note that ALK1 and ENG are both significantly reduced in BMPR2-deficient ECs. (C) Verification of increased ITGB1 expression in BMPR2-deficient ECs by qRT-PCR analysis (n = 6). (D) IGV browser displays over the ID3 loci showing SMAD1/5 ChIP-Seq track of HUVECs treated with BMP9 [53] and pSMAD1/5 ChIP-Seq track of MDA-MB-231 cells treated with TGFβ1 [41]. ChIP-Seq data were retrieved from the GEO (GSM684747, GSM2429820). (E) SMAD1 occupancy at the ID3 promoter was validated by ChIP-qPCR in steady-state conditions. IPs are a representative experiment of two, and ChIP-qPCR was performed in triplicates shown with means + SD. (F) Verification of altered ID1, ID2, and ID3 expression in BMPR2-deficient ECs by qRT-PCR analysis (n ≥ 4). Statistical significance relative to BMPR2wt was calculated for RPKM values using one-way ANOVA and Bonferroni post hoc test and for qRT-PCR data using the Kruskal-Wallis test with post hoc Dunn test; *P < 0.05, **P < 0.01, ***P < 0.001, ****P < 0.001. See S3 Data for underlying data. n.s., not significant (TIF) [file pbio.3000557.s004.tif]

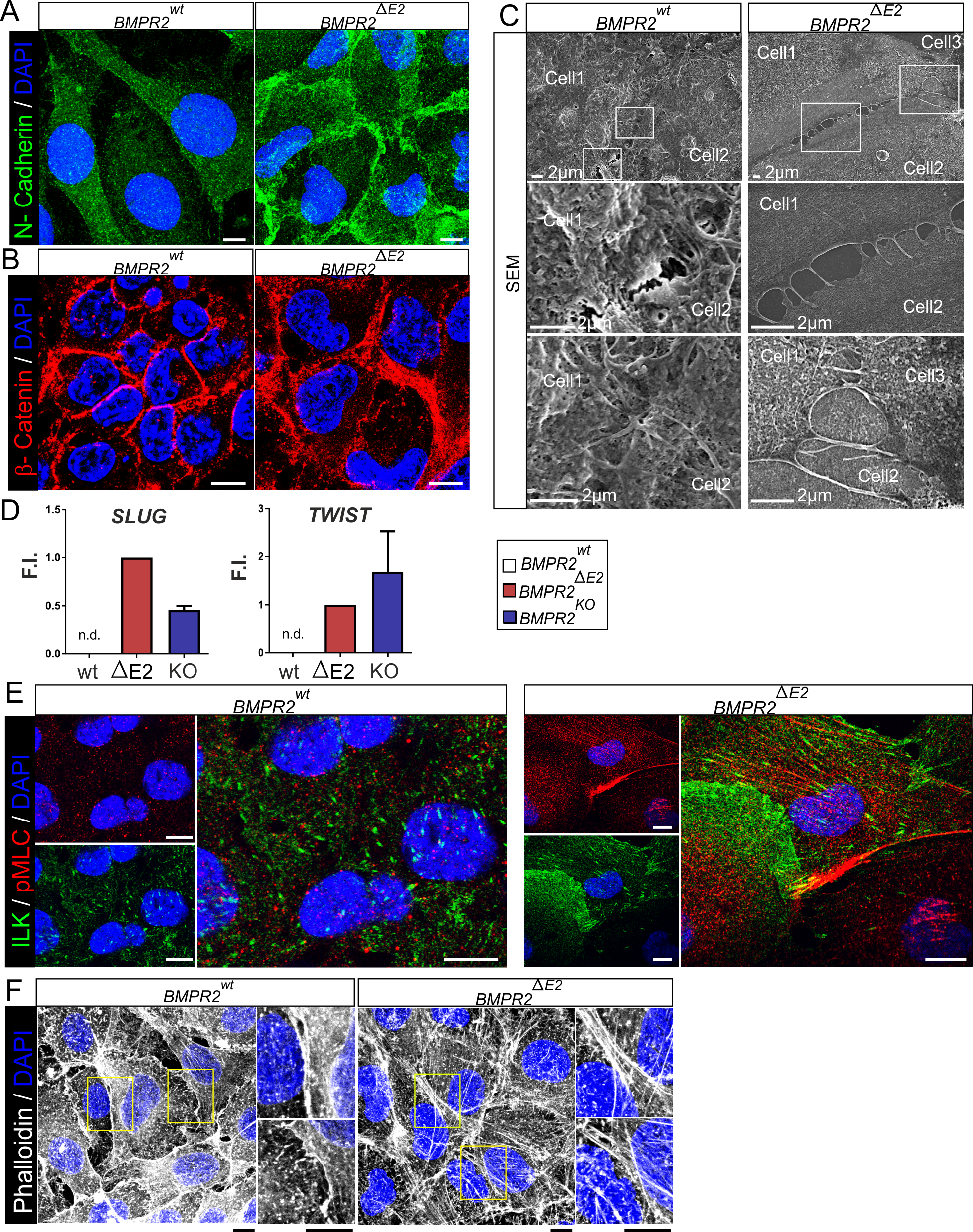

Supplement: S5 Fig — (A) Maximum projection of confocal z-stacks showing cell junctions of indicated cell clones immuno-labelled with an anti-N-Cadherin (green) antibody. (B) Single confocal z-planes (medial) showing cell junctions of indicated cell clones immuno-labelled with an anti-β-catenin (red) antibody. Scale bars, 10 μm. (C) SEM micrographs of indicated cell clones, showing different organization of CCC sites between 2–3 neighboring cells (indicated). Figure enlargements with higher resolution (below) are indicated by white frame. (D) qRT-PCR of indicated cell clones for EndMT transition markers SNAIL and TWIST under steady-state growth conditions. Values are expressed as mean F.I. relative to BMPR2ΔE2 + SD (n = 3 independent experiments). See S4 Data for underlying data. (E) Representative single confocal z-planes showing the distribution of pMLC (red) and ILK (green) of indicated cell clones. Scale bars, 10 μm. (F) Maximum projections of basal-to-apical confocal z-planes of indicated ECs stained with Phalloidin (white pseudo-color). See S1 Movie. Figure enlargements are indicated by yellow frame. F.I., fold induction; ILK, integrin-linked kinase; n.d., not detected; SEM, scanning electron microscopy (TIF) [file pbio.3000557.s005.tif]

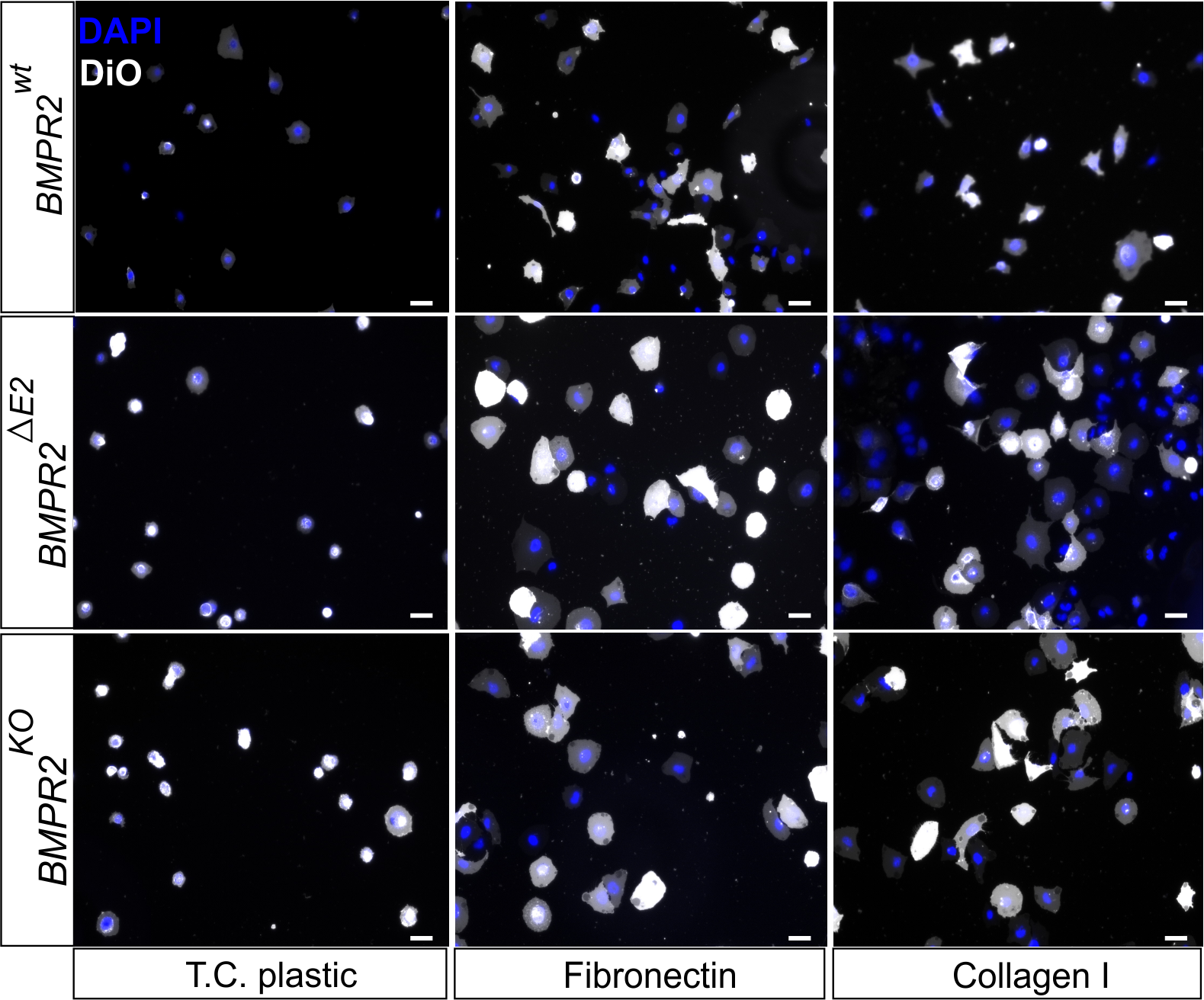

Supplement: S6 Fig — Cell adhesion on dishes (TC plastic) coated with ECM proteins (all 5 μg/cm2). Representative pictures of cells, which were seeded on TC plastic, FN, or collagen I and counterstained using DiO (pseudo-color white) labelling and DAPI. Spreading area is quantified in Fig 5B (n = 3 independent experiments). Scale bars, 50 μm. TC, tissue culture. (TIF) [file pbio.3000557.s006.tif]

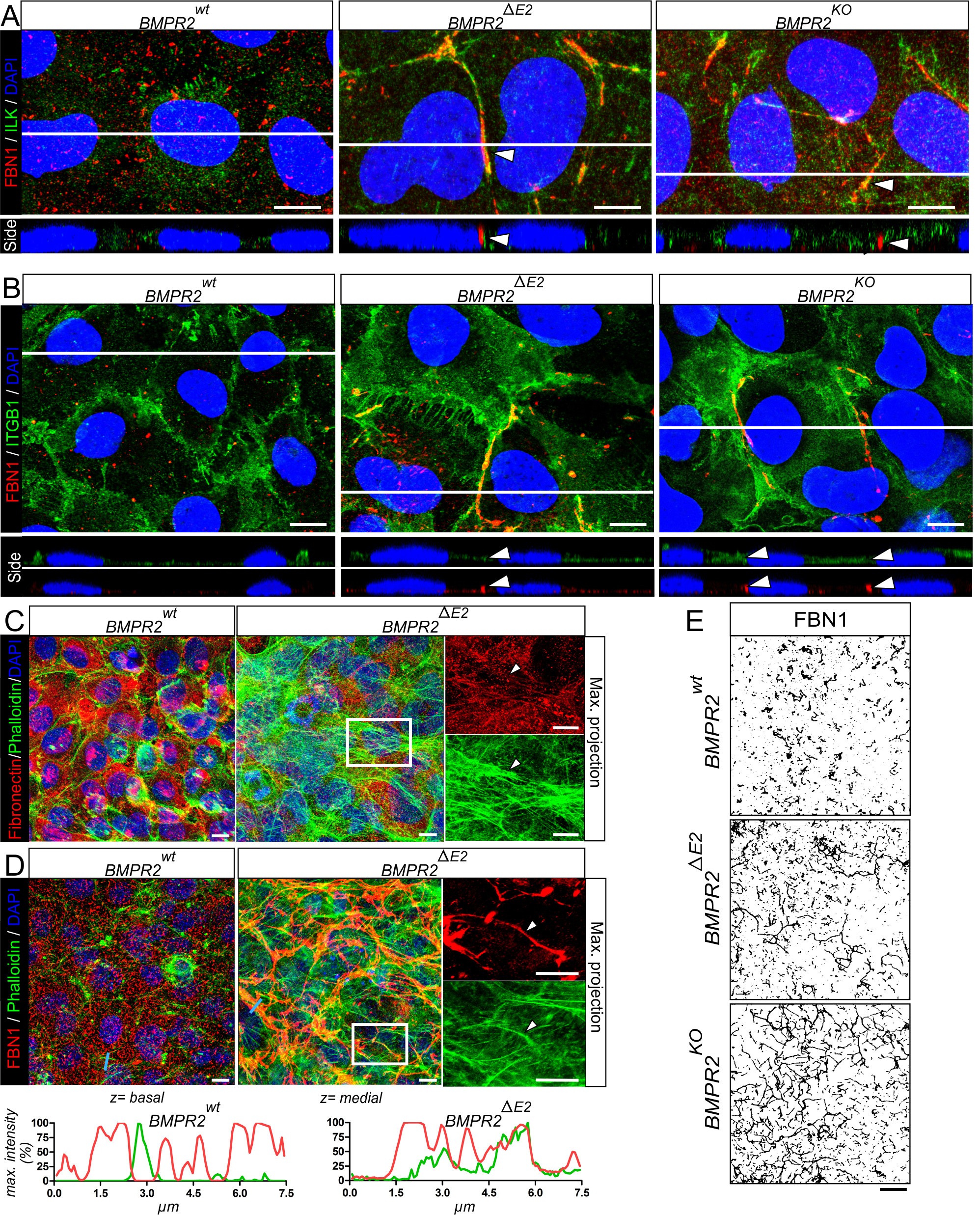

Supplement: S7 Fig — (A) Maximum projection of confocal z-stacks immunostained for ILK (green) and FBN1 (red) for indicated cell clones (upper). Side-view projection of single confocal z-plane indicated by white line in upper. Relative localization of ILK (green) and FBN1 (red) is indicated by white arrowhead. See also S3 Movie. (B) Single confocal z-planes (medial) immunostained for ITGB1 (green) and FBN1 (red) for indicated cell clones (upper). Side projection of confocal plane indicated by white line in upper. Relative localization of ITGB1 (green) and FBN1 (red) is indicated by white arrowhead. See also S4 Movie. (C) Maximum projection of confocal z-stacks immunostained for endogenous Fn (red) and the F-actin cytoskeleton (green) of indicated cell types (left). Figure enlargement depicting relative localization of Fn fibers (red) and bundles of filamentous actin (green) (right; white arrowhead). (D) Maximum projection of confocal z-stacks immunostained for FBN1 (red) and F-actin (green) of indicated cell clones (left). Figure enlargement depicting relative localization of FBN1 fibers (red) and bundles of filamentous actin (green) (right; white arrowhead). Line scans (blue line, upper) of single confocal z-planes (indicated) showing maximum signal intensity in percent (lower). (E) Characterization of decellularized deposits of FBN1 (binarized images of single confocal z-planes) from BMPR2wt or BMPR2-deficient cells (upper). Scale bars represent 10 μm. (TIF) [file pbio.3000557.s007.tif]

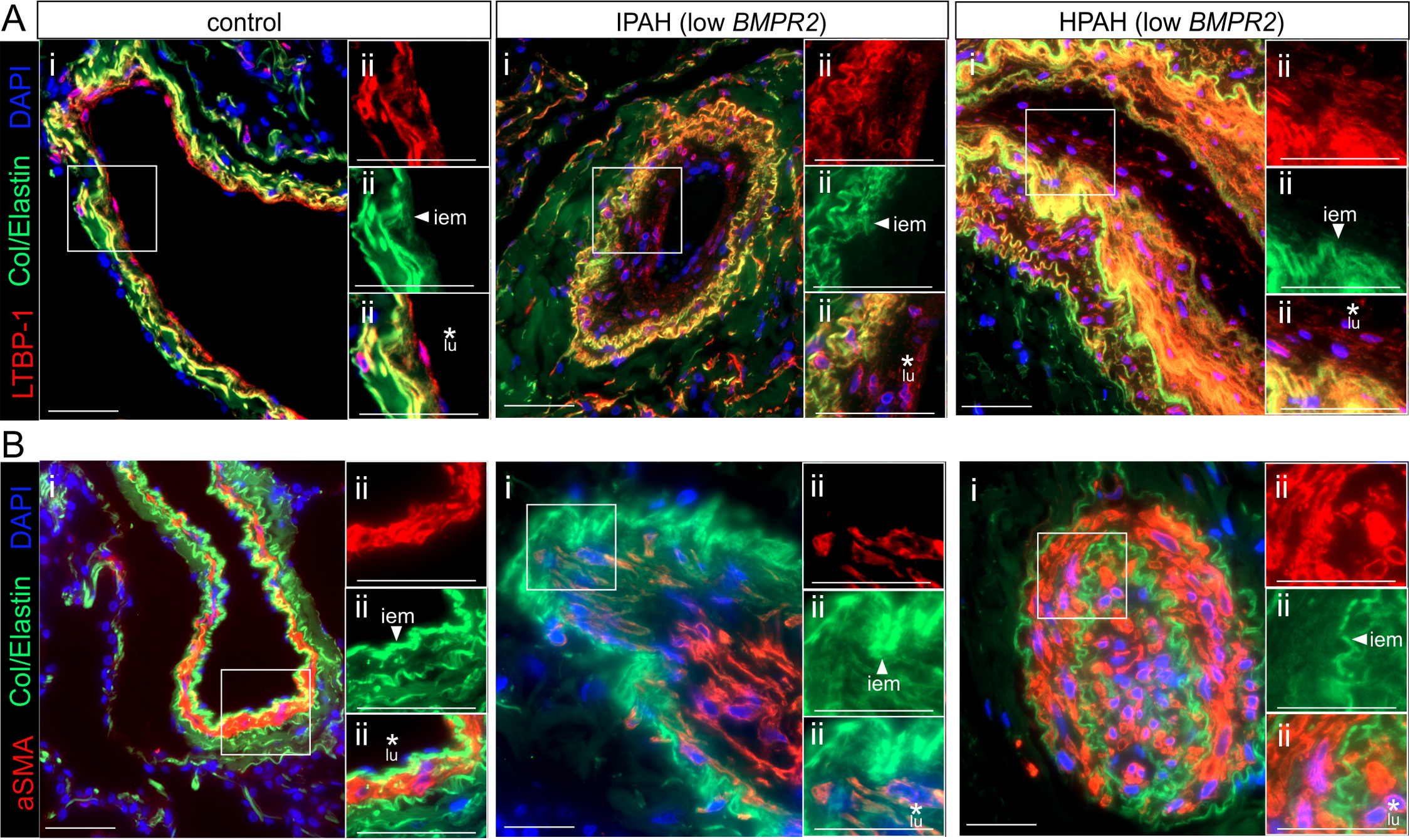

Supplement: S8 Fig — (A–B) Representative PAs of control, IPAH, and HPAH donors were stained for LTBP-1 or αSMA (red), collagen, and elastin at approximately 520 nm emission (green) and DAPI (blue) (i). (A) Higher magnification of the area surrounding the iem (ii) shows co-localization of LTBP-1 with the elastic membrane in the tm and sub-EC layer (e.g., basal lamina) in controls, while LTBP-1 staining similar to FBN1 (Fig 7D) exceeds the iem toward the lumen of PAs from IPAH (middle) and HPAH (right) donors. (B) αSMA staining (red) was restricted to the tm in controls, while IPAH/HPAH PAs αSMA stained additional the intima and lumen. Scale bar represents 50 μm. lu, lumen; tm, tunica media. (TIF) [file pbio.3000557.s008.tif]

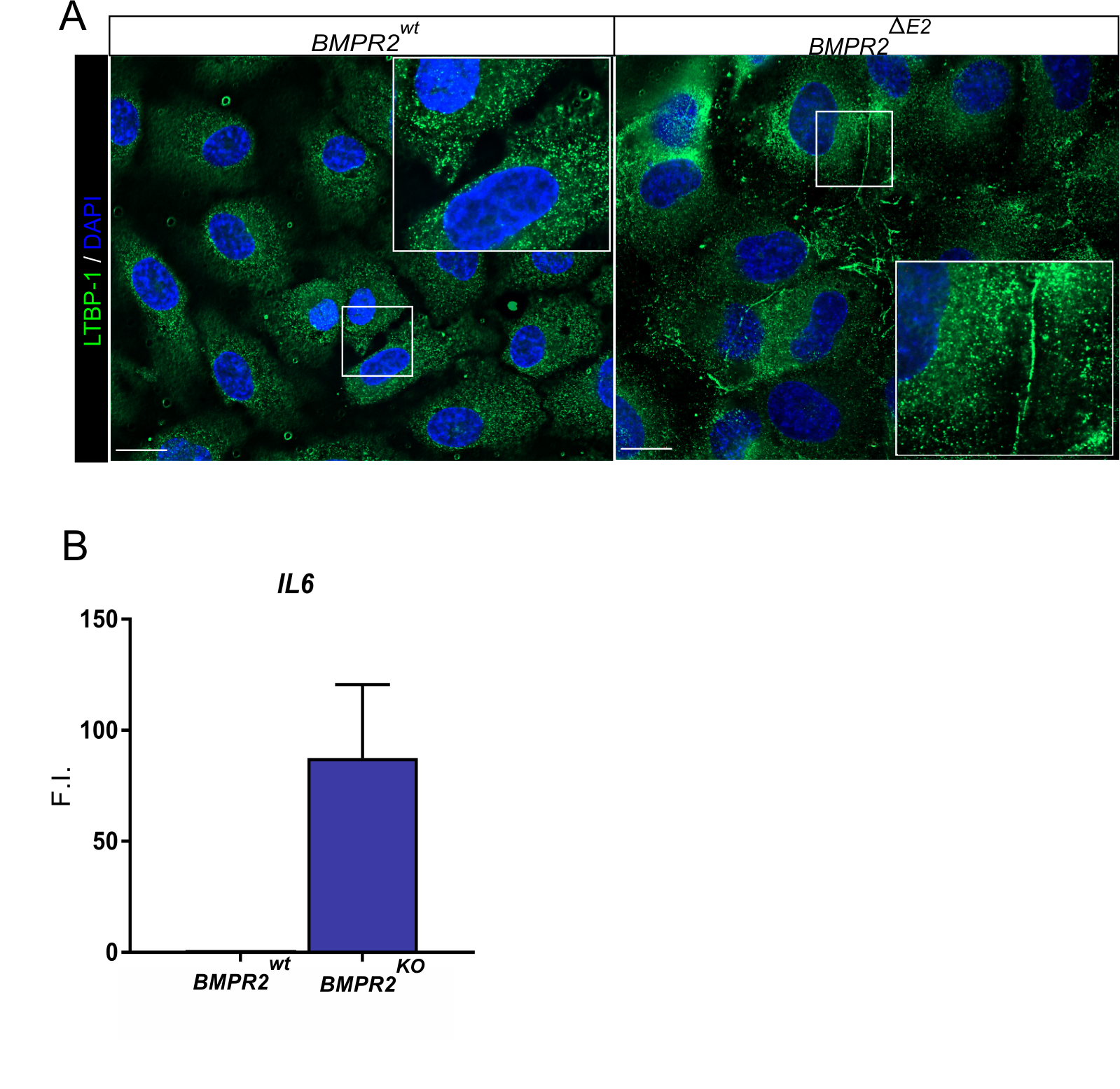

Supplement: S9 Fig — (A) Immunocytochemical stainings against LTBP-1 by indicated cell clones. Note the junctional accumulation of LTBP-1 puncta in BMPR2-deficient cells. Region of interest zoom-in are shown in rectangular boxes. (B) qRT-PCR under steady-state culture conditions for indicated cell clones. Scale bars represent 20 μm. Values are expressed as fold induction (n = 3 independent experiments). Data are shown as mean + SD relative to BMPR2wt. See S4 Data for underlying data. F.I., fold induction (TIF) [file pbio.3000557.s009.tif]
