# Supplementary material for: BMPR2 acts as a gatekeeper to protect endothelial cells from increased TGFβ responses and altered cell mechanics
Source: PLoS Biol. 2019 Dec 11;17(12):e3000557. doi: 10.1371/journal.pbio.3000557 (PMC6927666; doi:10.1371/journal.pbio.3000557)
Supplement: S1 Raw Images — (PDF) [file pbio.3000557.s022.pdf]

A

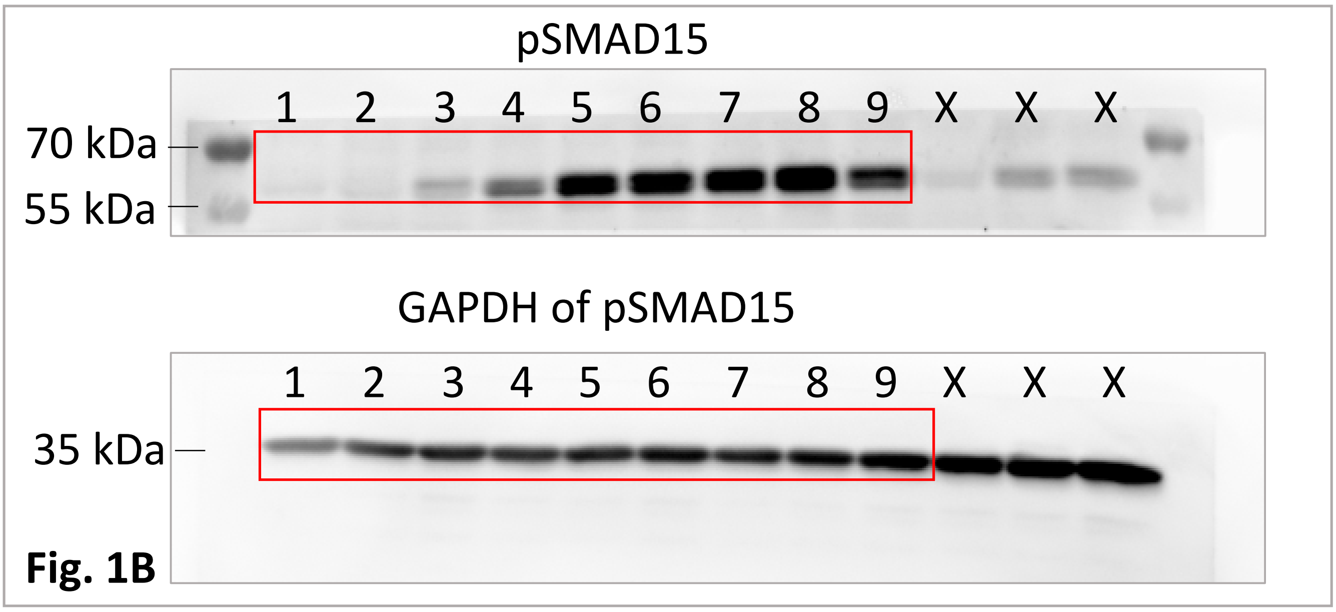
pSMAD15
1 2 3 4 5 6 7 8 9 X X X
70 kDa
55 kDa
GAPDH of pSMAD15
1 2 3 4 5 6 7 8 9 X X X
35 kDa
Fig. 1B

B

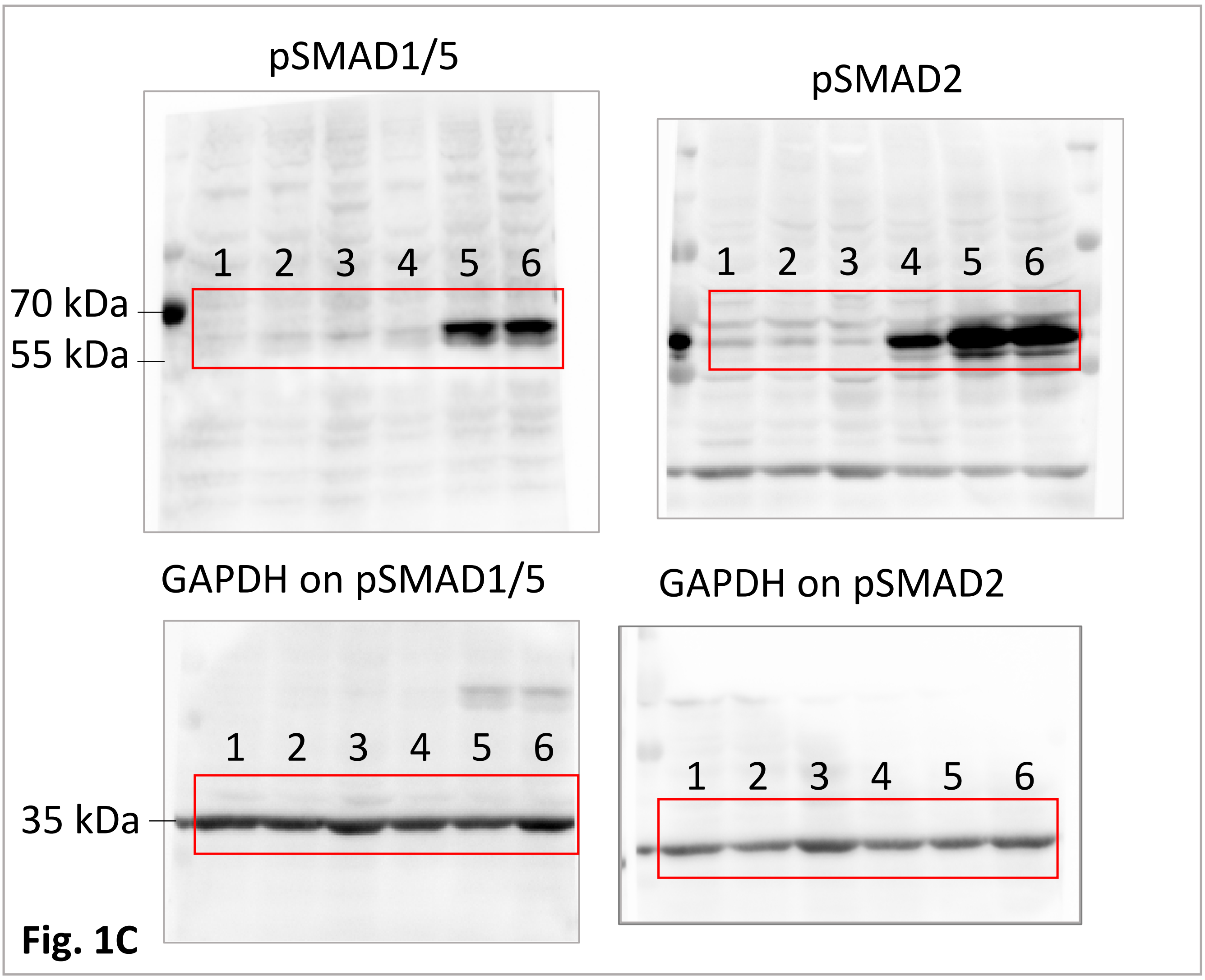
pSMAD1/5
1 2 3 4 5 6
70 kDa
55 kDa
pSMAD2
1 2 3 4 5 6
GAPDH on pSMAD1/5
1 2 3 4 5 6
35 kDa
GAPDH on pSMAD2
1 2 3 4 5 6
Fig. 1C

C

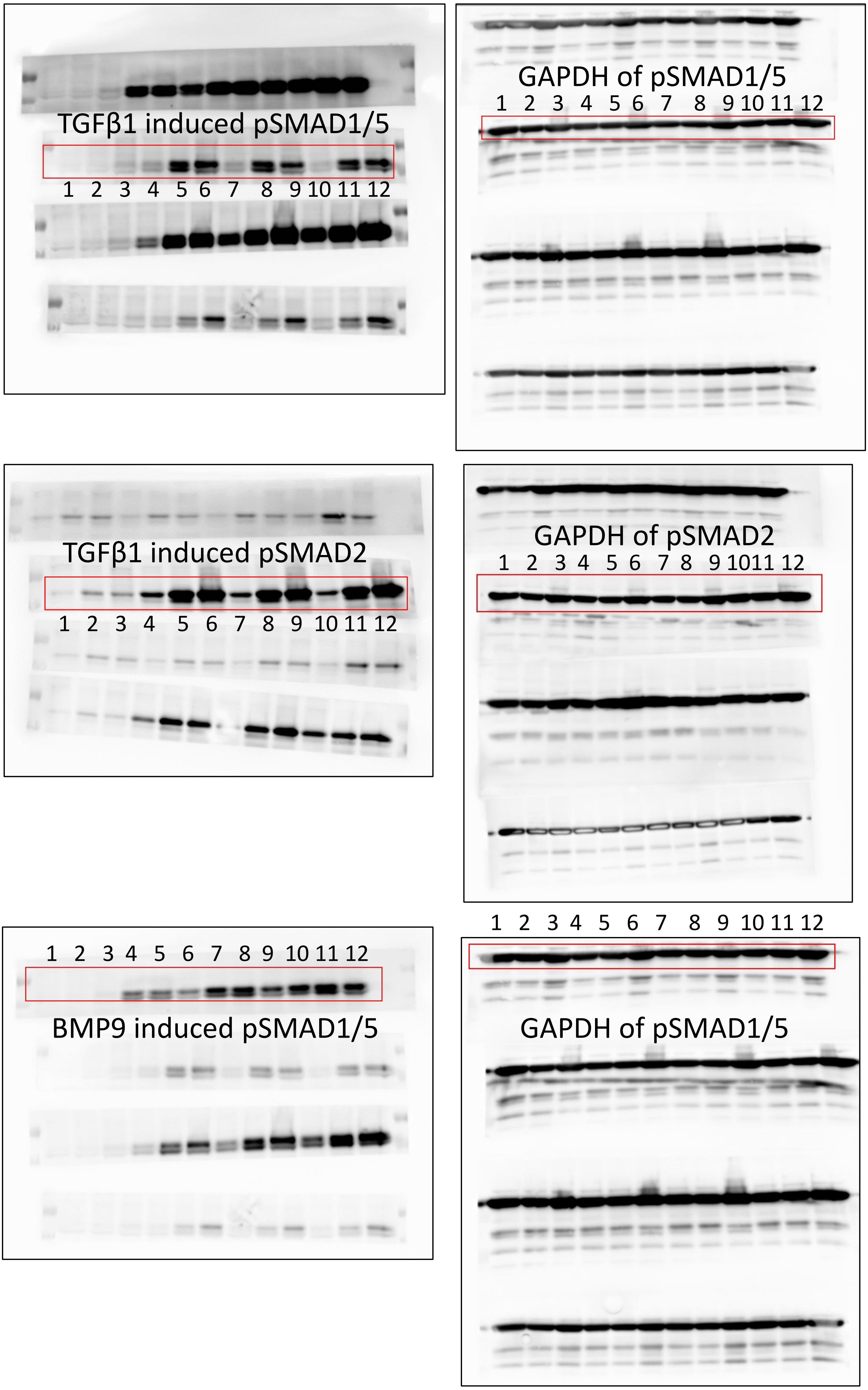
TGFβ1 induced pSMAD1/5
1 2 3 4 5 6 7 8 9 10 11 12
GAPDH of pSMAD1/5
1 2 3 4 5 6 7 8 9 10 11 12
TGFβ1 induced pSMAD2
1 2 3 4 5 6 7 8 9 10 11 12
GAPDH of pSMAD2
1 2 3 4 5 6 7 8 9 1011 12
1 2 3 4 5 6 7 8 9 10 11 12
BMP9 induced pSMAD1/5
1 2 3 4 5 6 7 8 9 10 11 12
GAPDH of pSMAD1/5

Fig. 1D

C

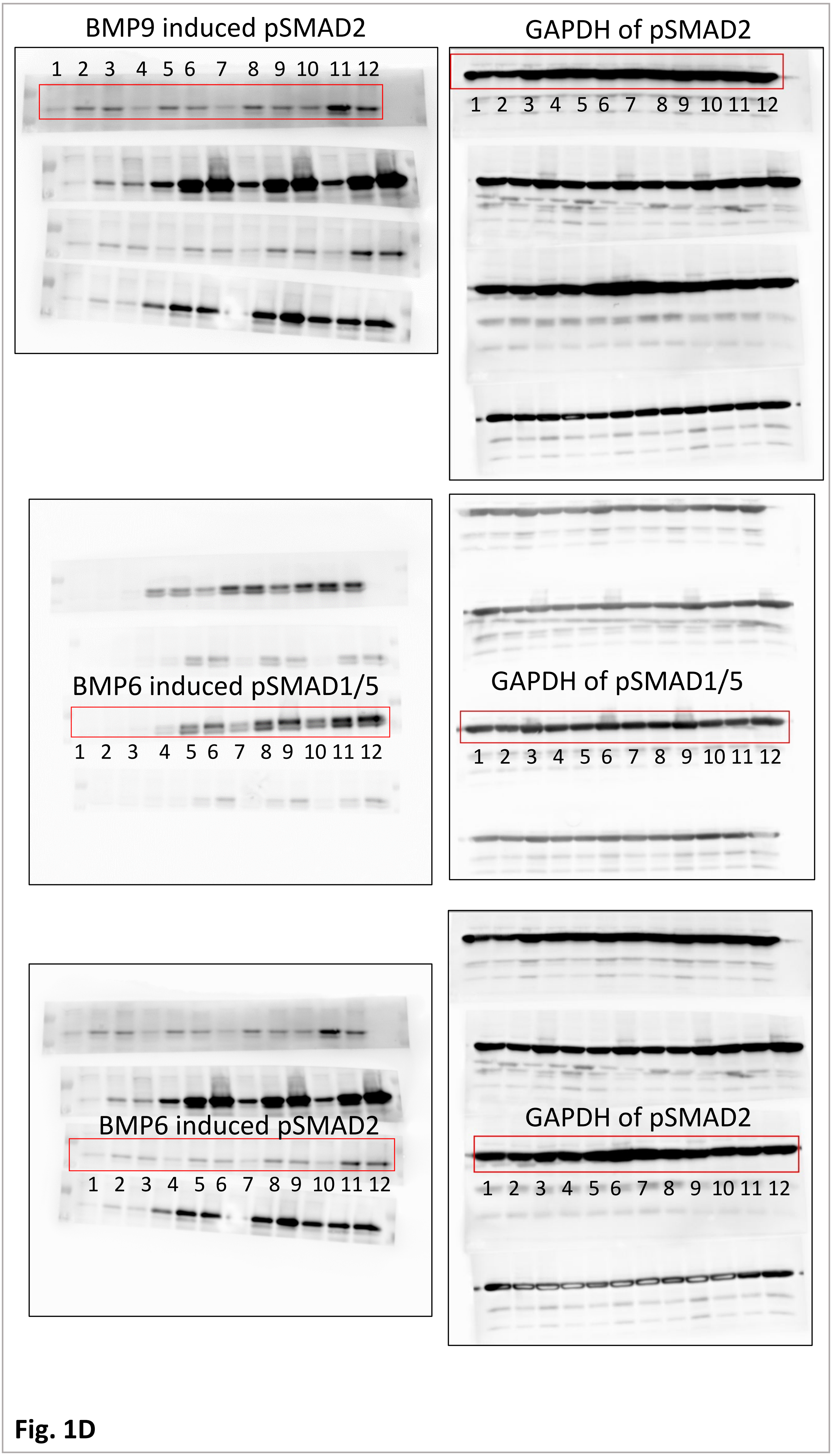
BMP9 induced pSMAD2
1 2 3 4 5 6 7 8 9 10 11 12
GAPDH of pSMAD2
1 2 3 4 5 6 7 8 9 10 11 12
BMP6 induced pSMAD1/5
1 2 3 4 5 6 7 8 9 10 11 12
GAPDH of pSMAD1/5
1 2 3 4 5 6 7 8 9 10 11 12
BMP6 induced pSMAD2
1 2 3 4 5 6 7 8 9 10 11 12
GAPDH of pSMAD2
1 2 3 4 5 6 7 8 9 10 11 12

Fig. 1D

D

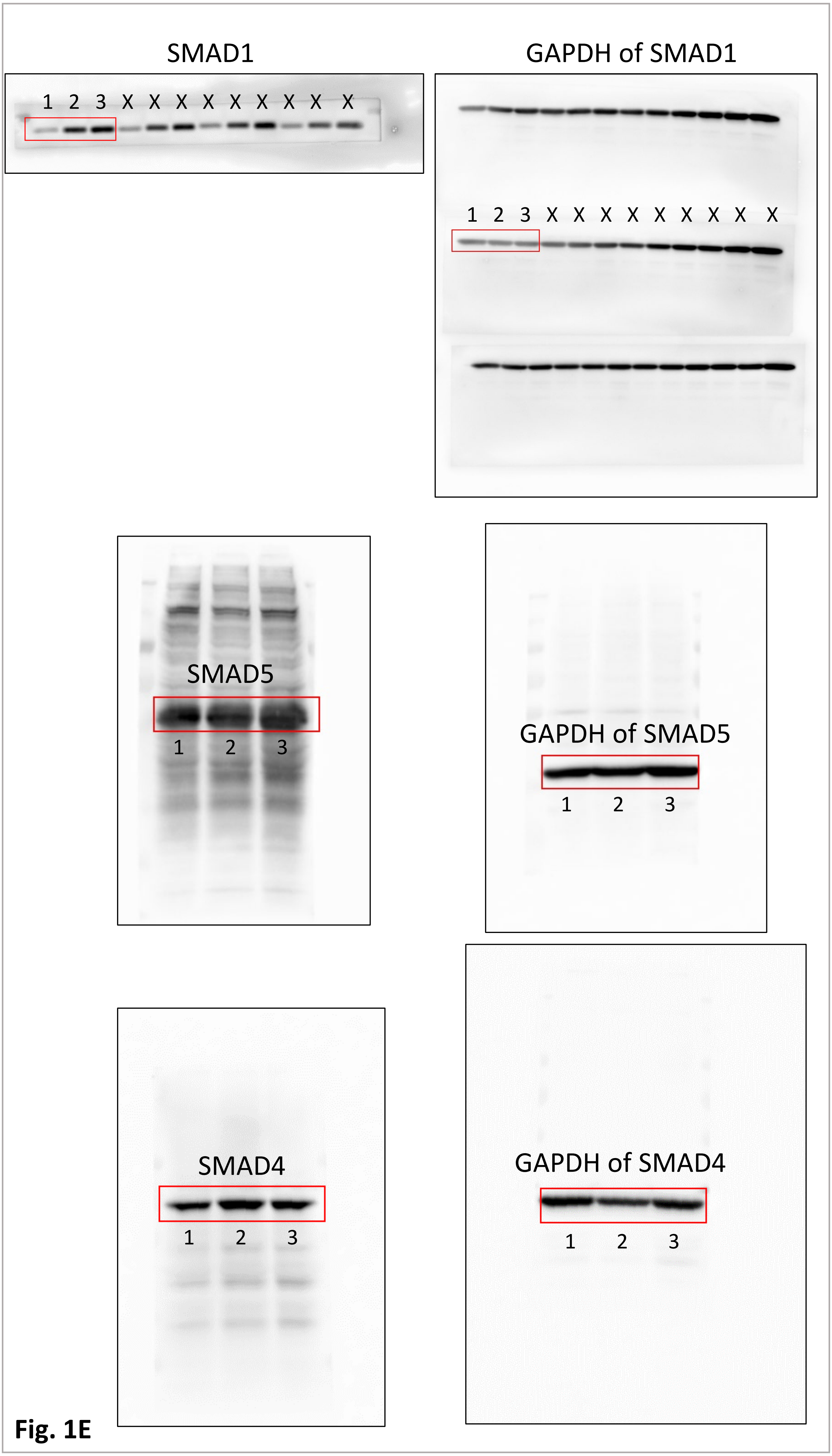
SMAD1
1 2 3 X X X X X X X X X
GAPDH of SMAD1
1 2 3 X X X X X X X X X
SMAD5
1 2 3
GAPDH of SMAD5
1 2 3
SMAD4
1 2 3
GAPDH of SMAD4
1 2 3

Fig. 1E

D

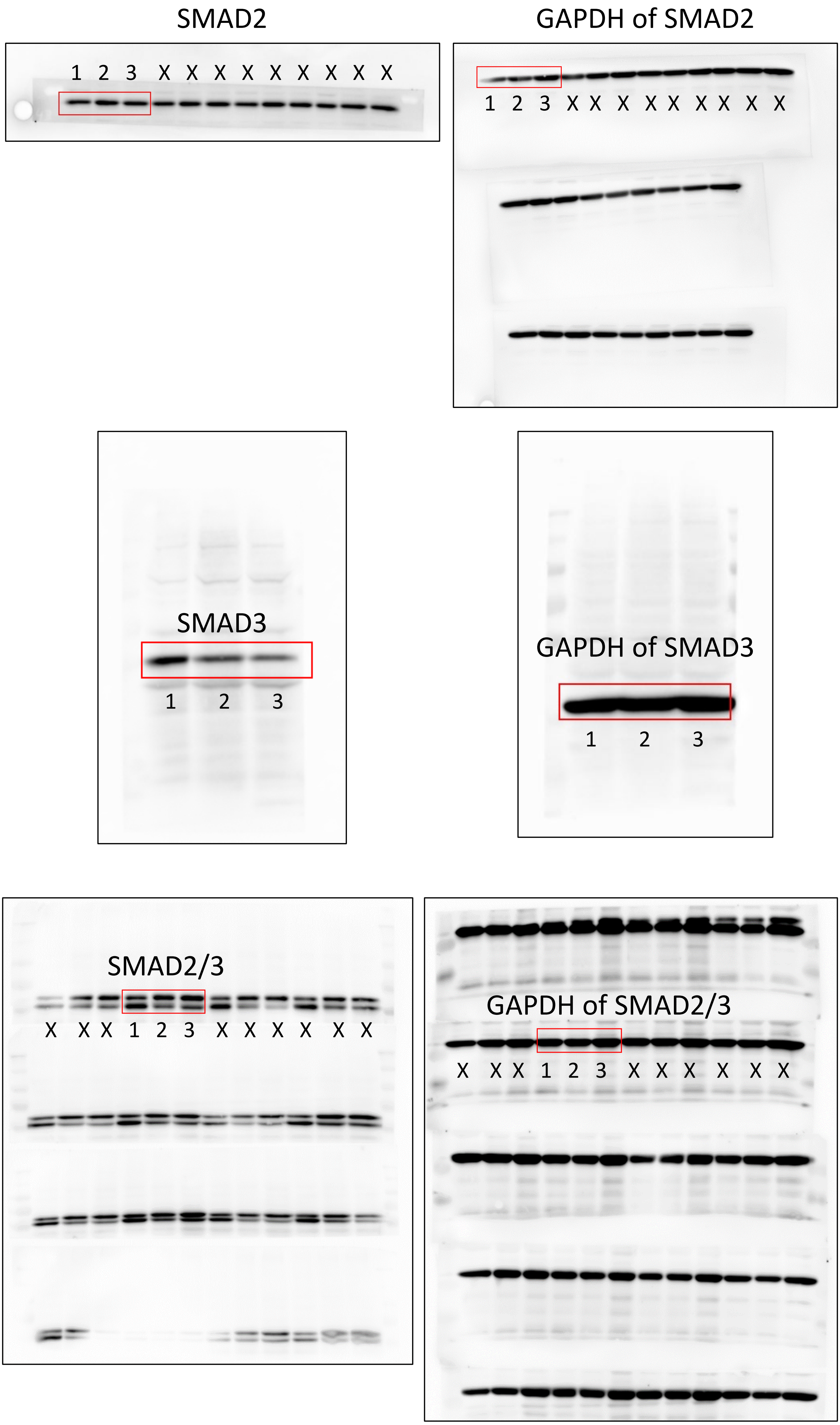
SMAD2
1 2 3 X X X X X X X X X
GAPDH of SMAD2
1 2 3 X X X X X X X X X
SMAD3
1 2 3
GAPDH of SMAD3
1 2 3
SMAD2/3
X X X 1 2 3 X X X X X X
GAPDH of SMAD2/3
X X X 1 2 3 X X X X X X

Fig. 1E

E

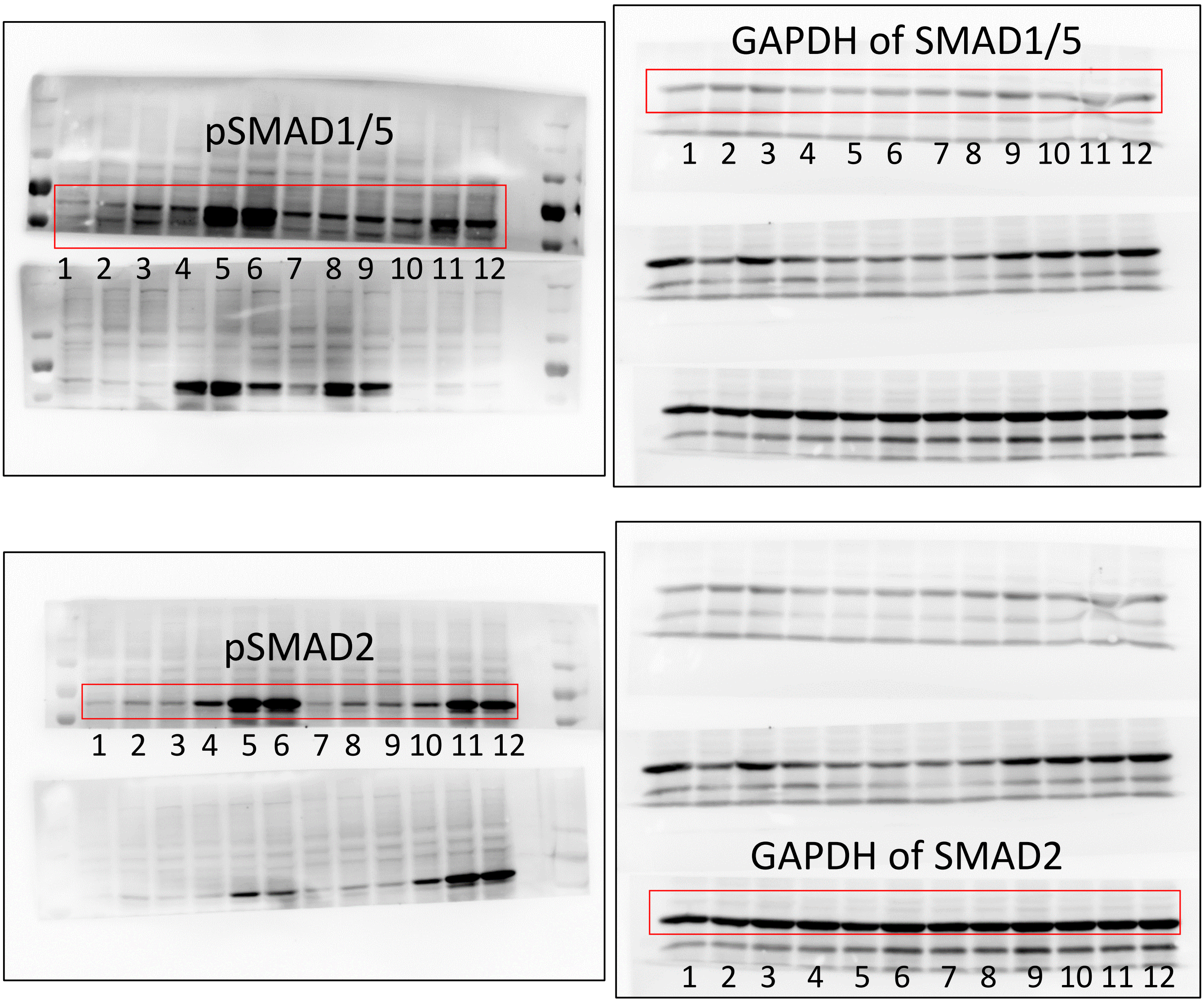

Fig. 2B

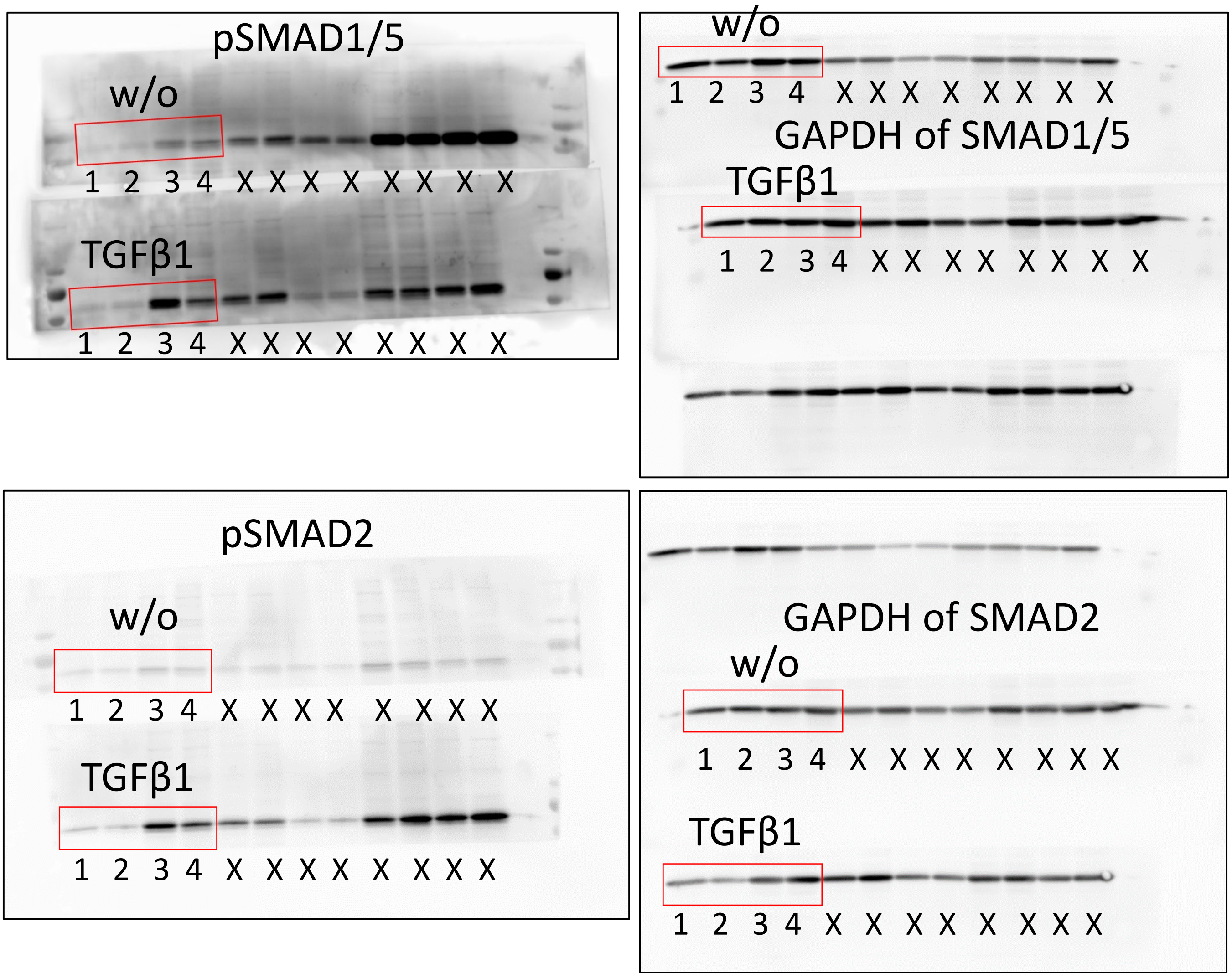

Fig. 2C

F

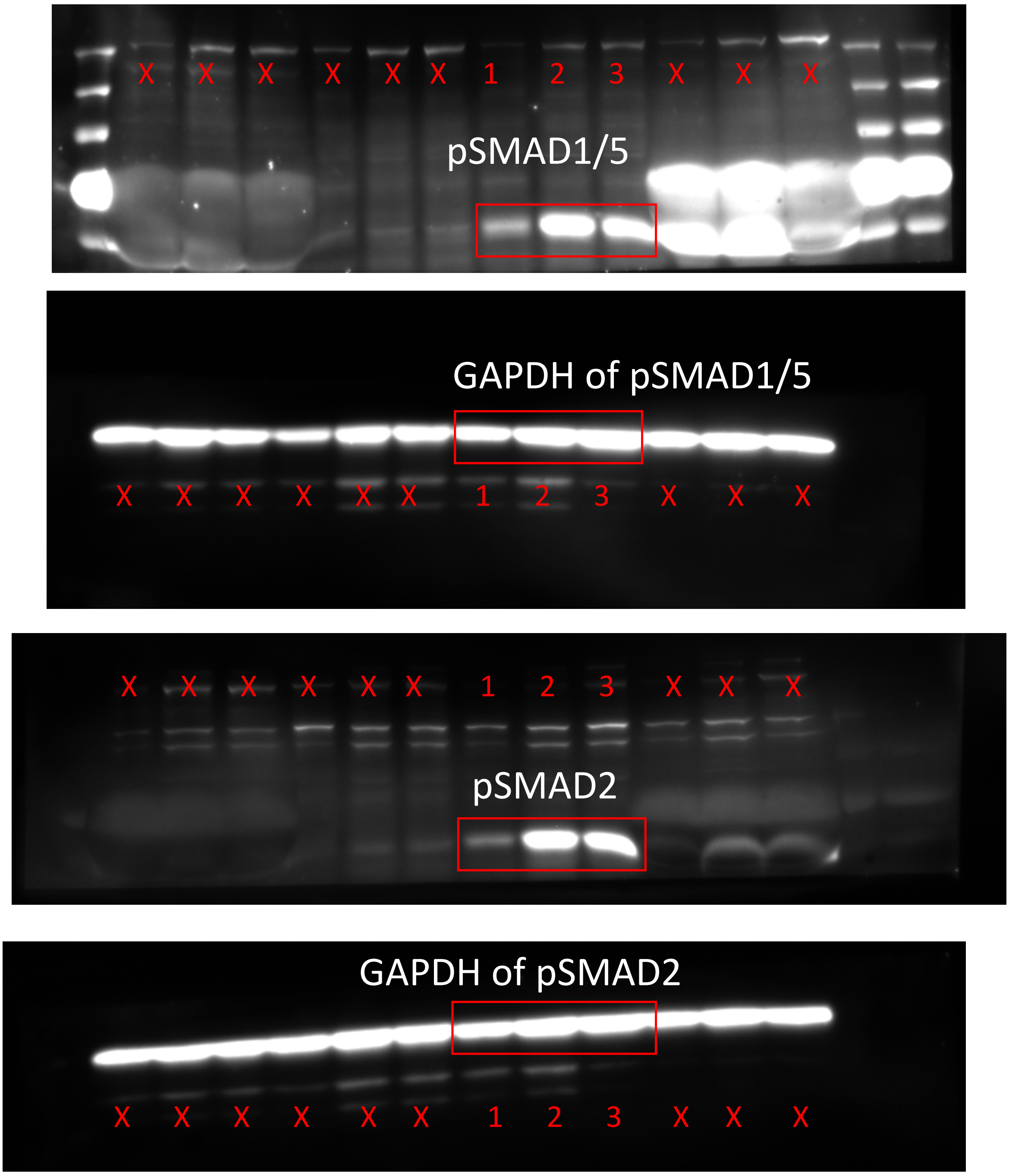
X X X X X X 1 2 3 X X X
pSMAD1/5
GAPDH of pSMAD1/5
X X X X X X 1 2 3 X X X
X X X X X X 1 2 3 X X X
pSMAD2
GAPDH of pSMAD2
X X X X X X 1 2 3 X X X

Fig. 3A

G

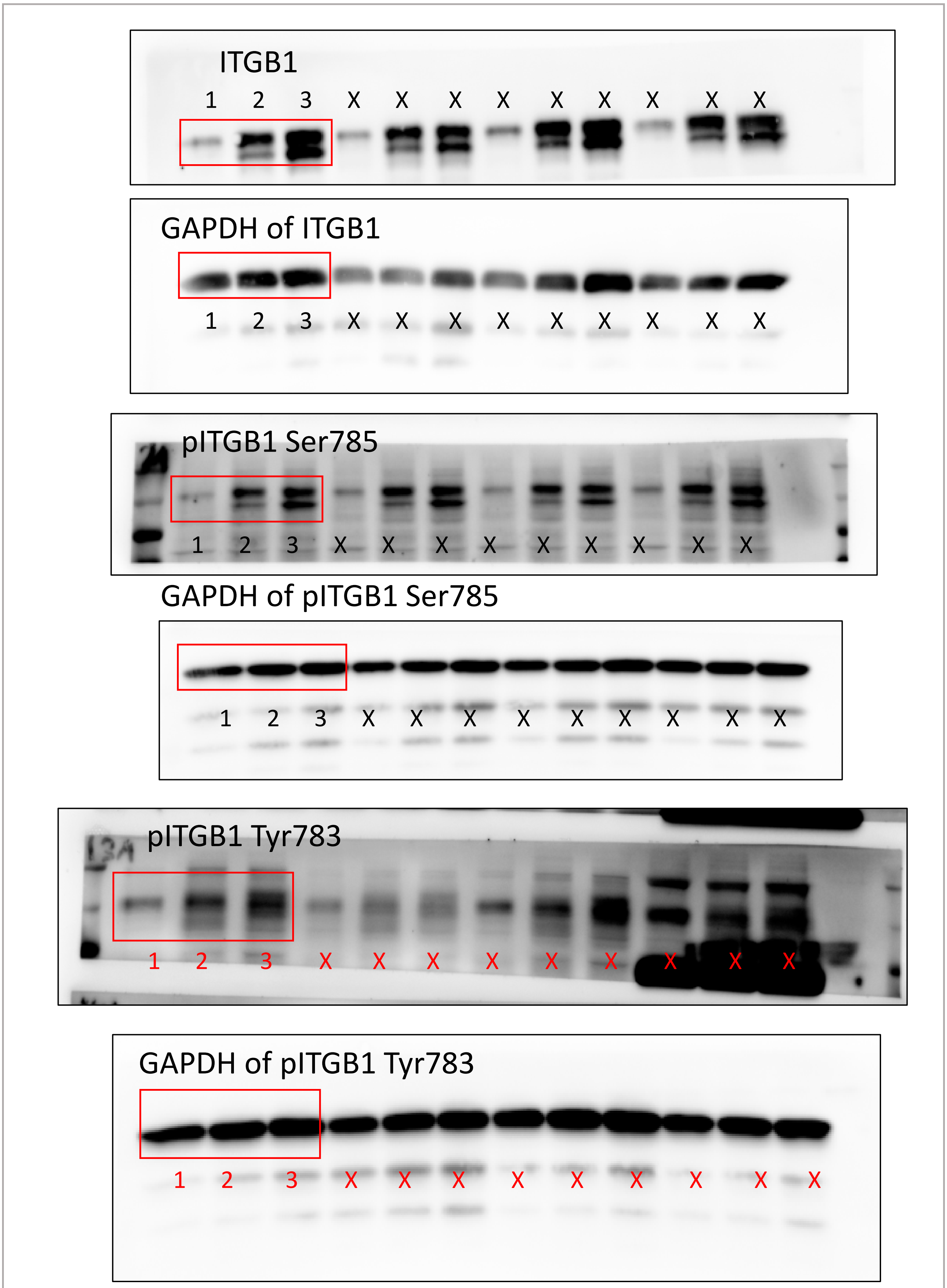
ITGB1
1 2 3 X X X X X X X X X
GAPDH of ITGB1
1 2 3 X X X X X X X X X
pITGB1 Ser785
1 2 3 X X X X X X X X X
GAPDH of pITGB1 Ser785
1 2 3 X X X X X X X X X
pITGB1 Tyr783
1 2 3 X X X X X X X X X
GAPDH of pITGB1 Tyr783
1 2 3 X X X X X X X X X

Fig. 4A

H

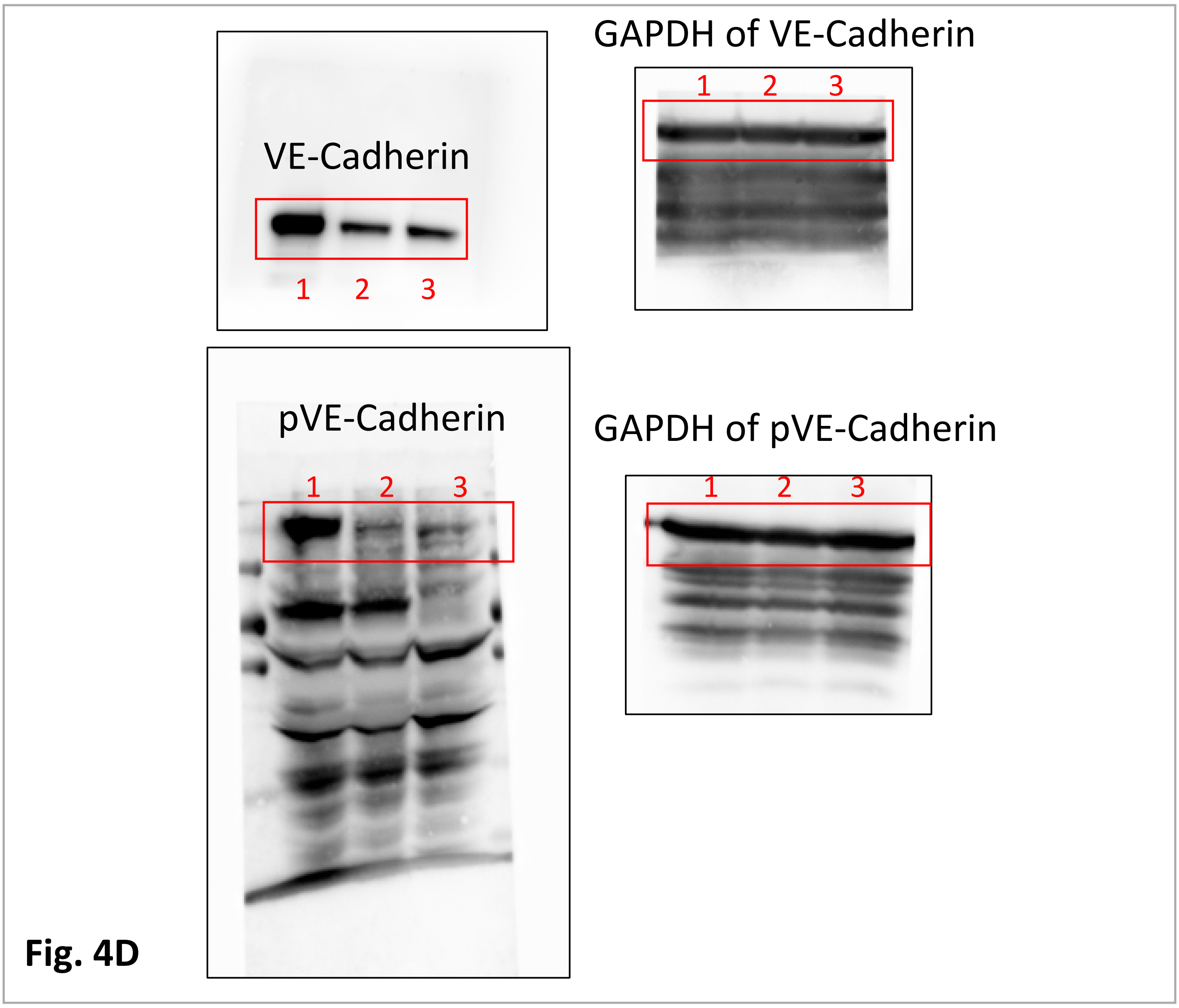

Fig. 4D

I

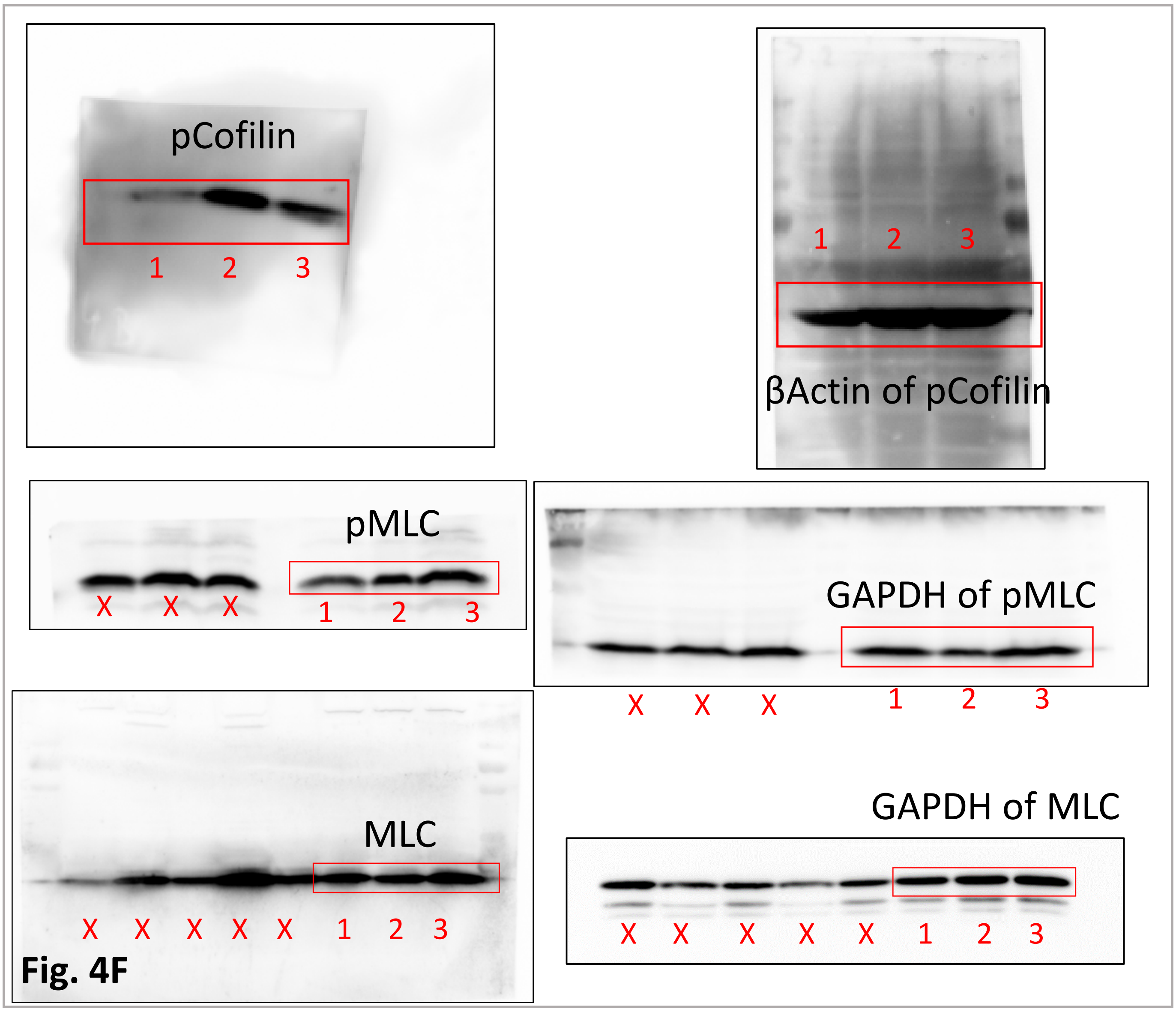

Fig. 4F

J

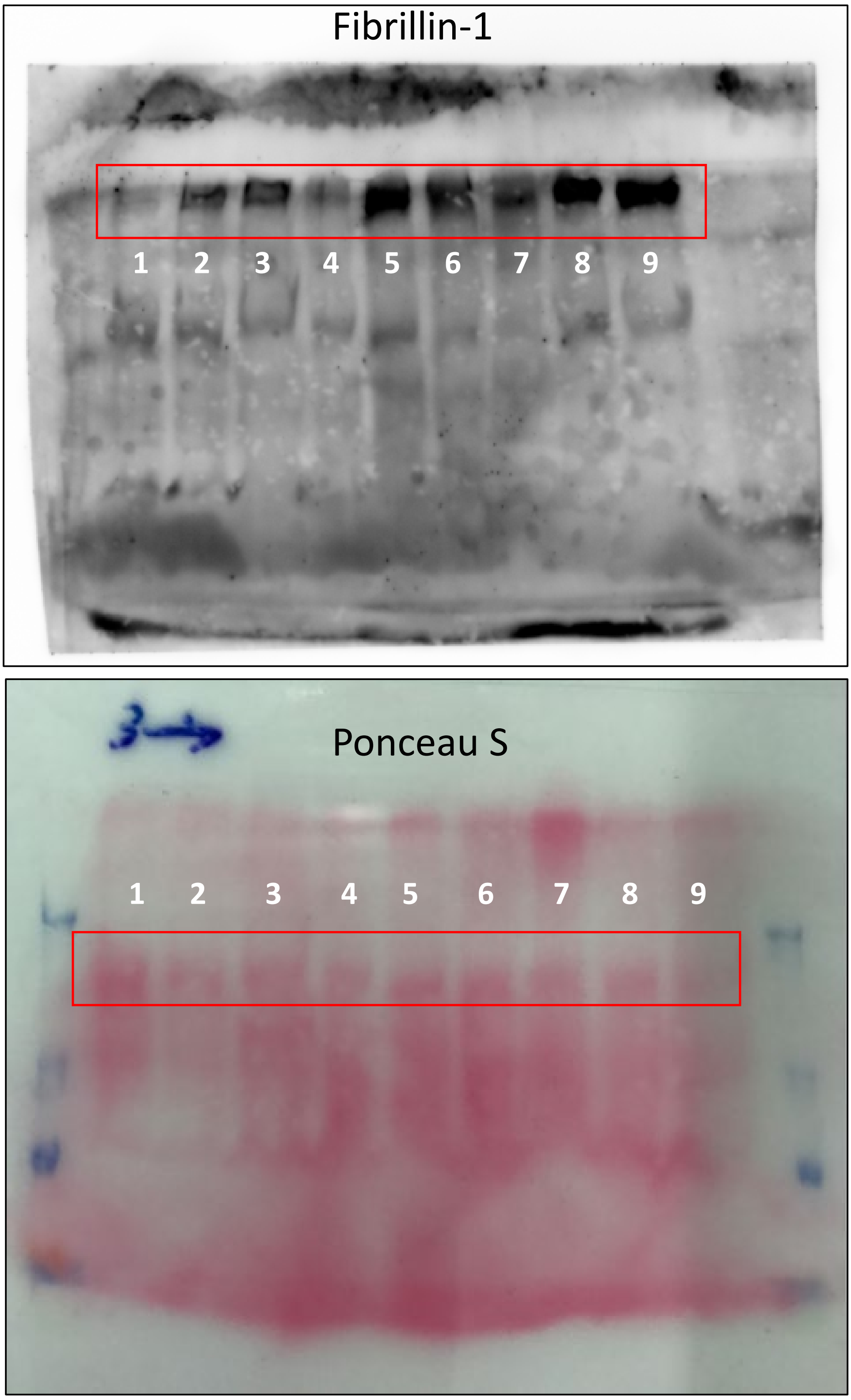

Fig. 6B

K

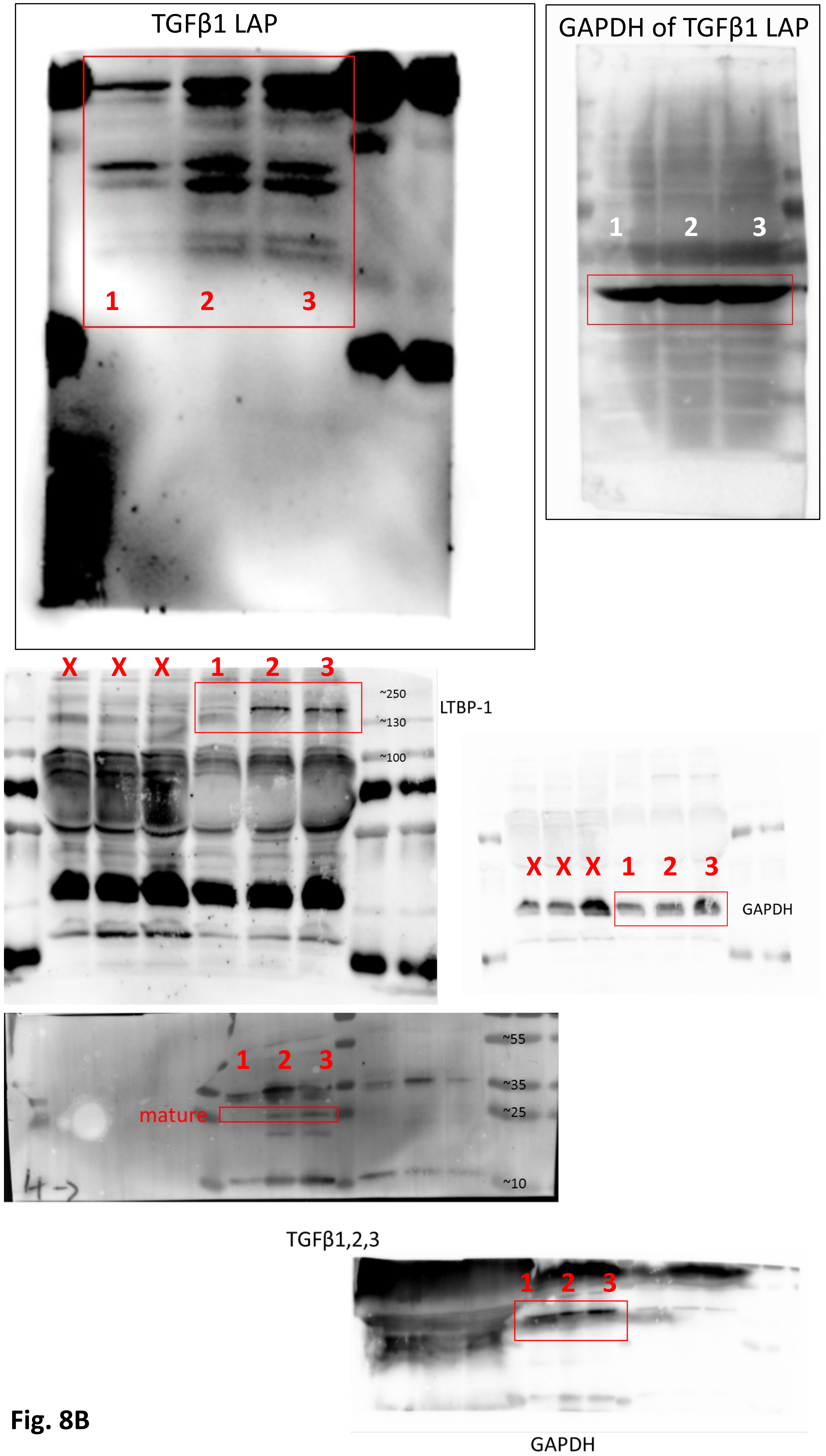
TGFβ1 LAP
1
2
3
GAPDH of TGFβ1 LAP
1
2
3
X X X 1 2 3
~250
~130
~100
LTBP-1
X X X 1 2 3
GAPDH
1 2 3
mature
~55
~35
~25
~10
TGFβ1,2,3
1 2 3
GAPDH

Fig. 8B

L

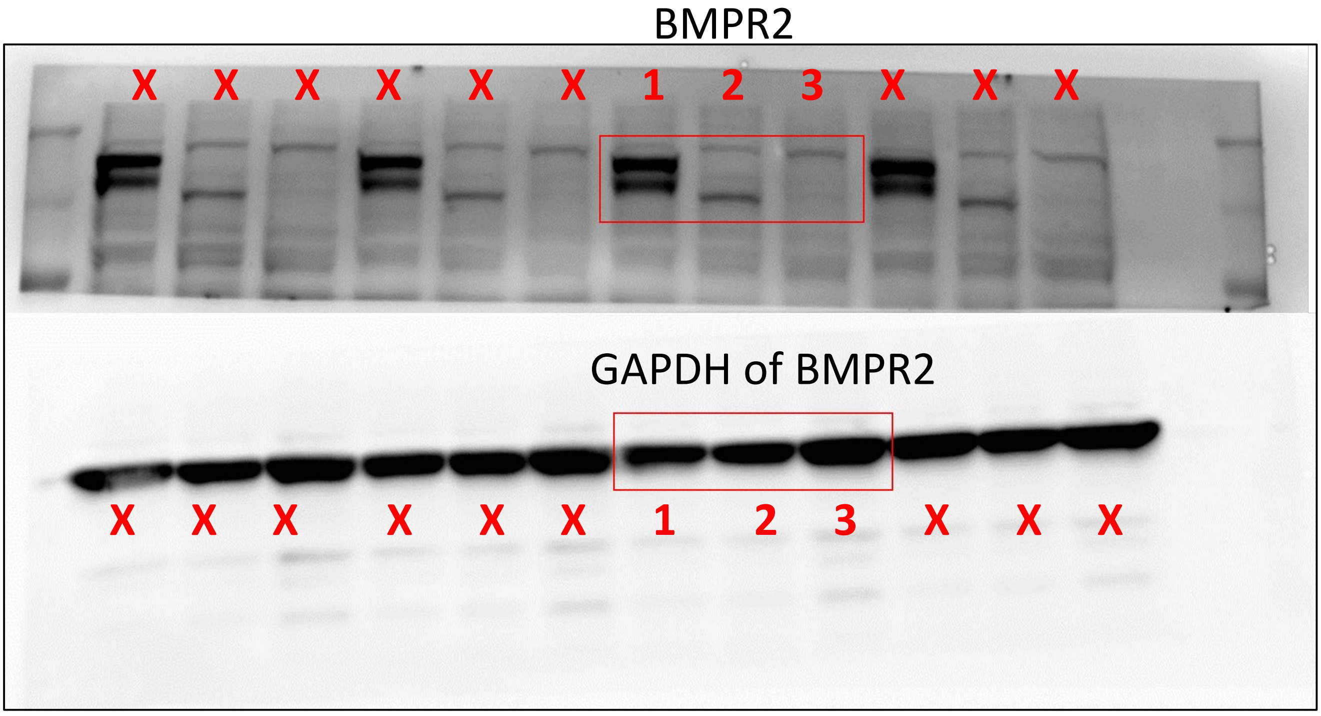

Fig. S1E

M

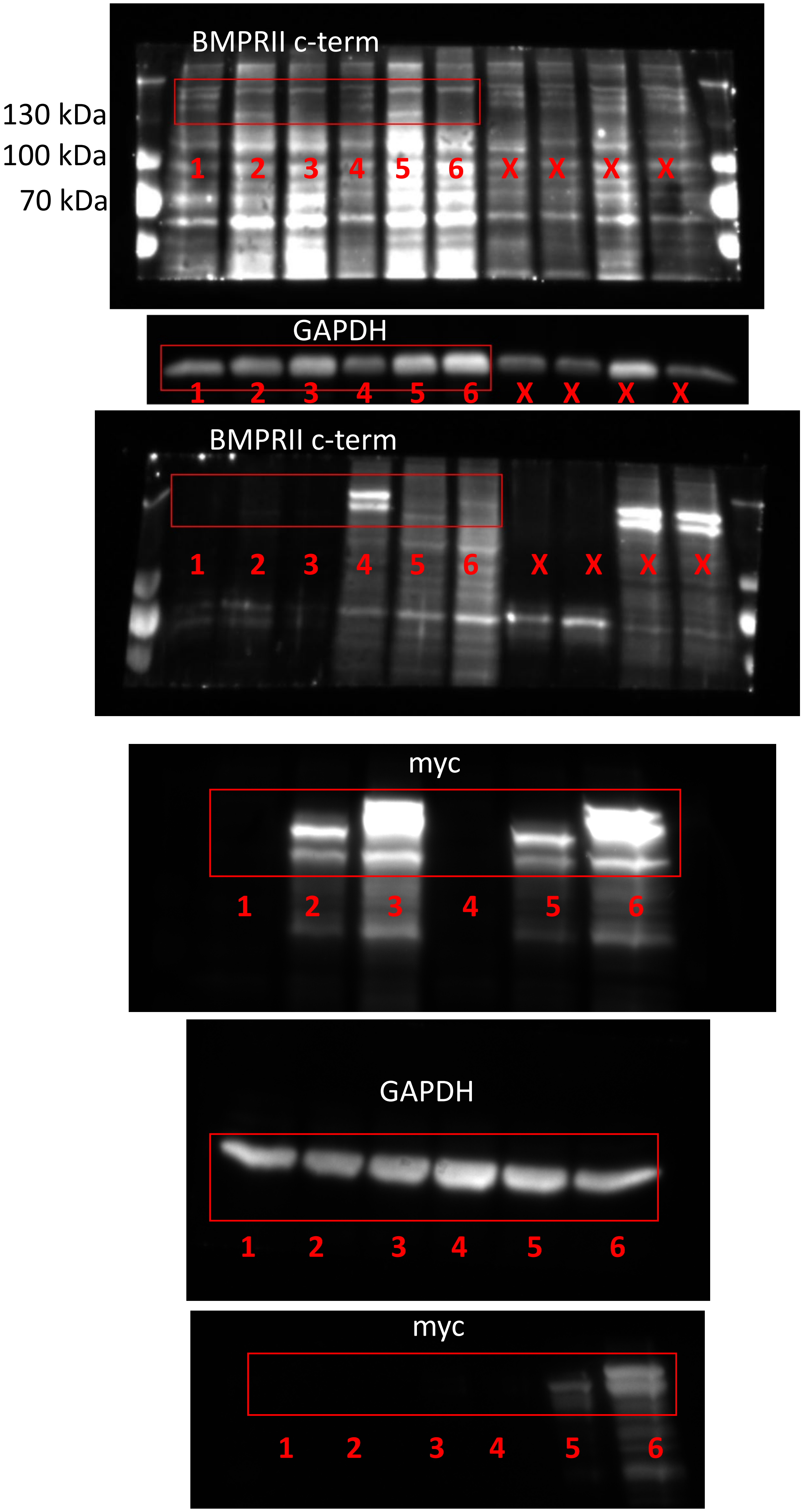
BMPRII c-term
130 kDa
100 kDa
70 kDa
1 2 3 4 5 6 X X X X
GAPDH
1 2 3 4 5 6 X X X X
BMPRII c-term
1 2 3 4 5 6 X X X X
myc
1 2 3 4 5 6
GAPDH
1 2 3 4 5 6
myc
1 2 3 4 5 6
Fig. S1F

N

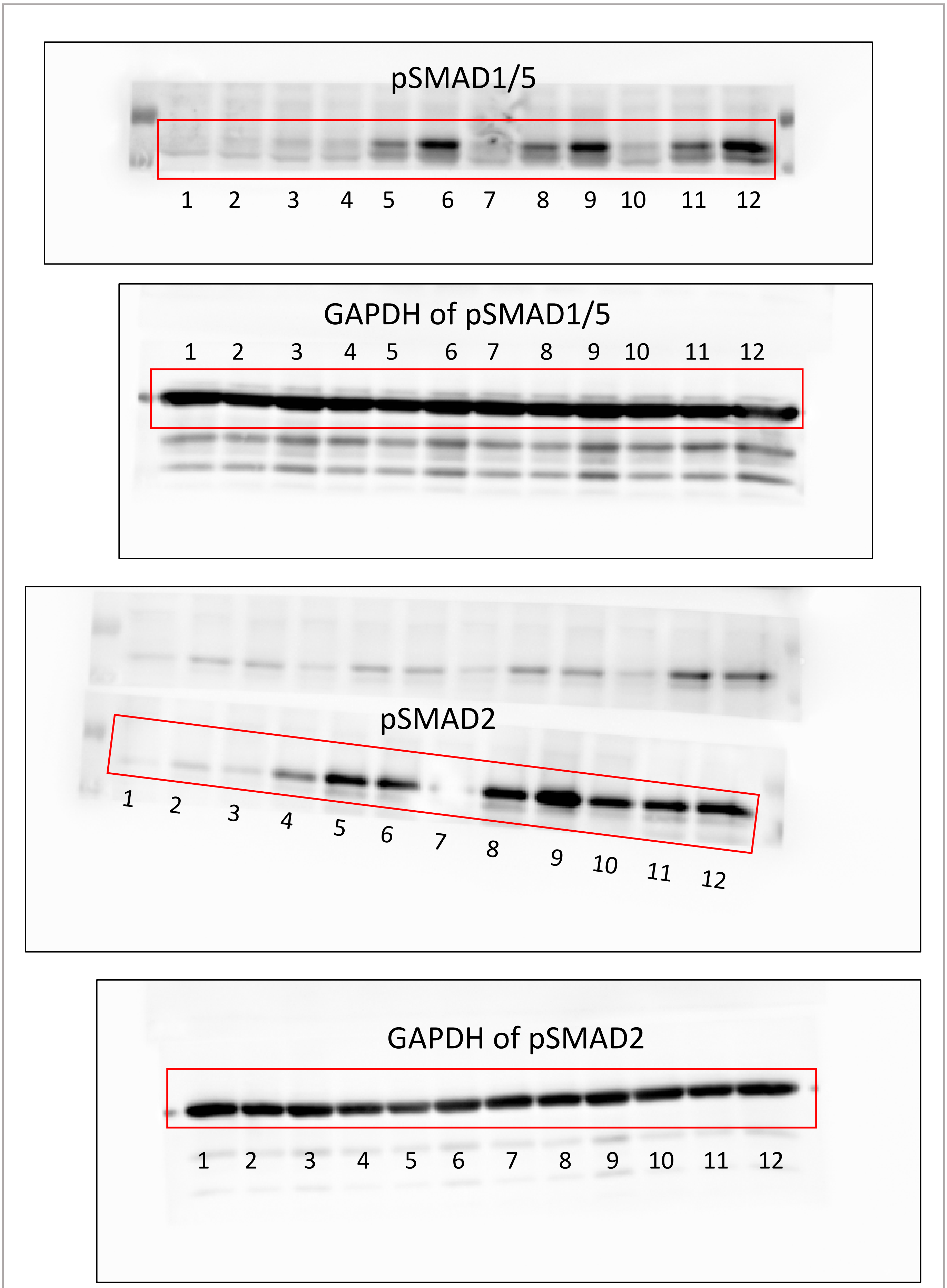
pSMAD1/5
1 2 3 4 5 6 7 8 9 10 11 12
GAPDH of pSMAD1/5
1 2 3 4 5 6 7 8 9 10 11 12
pSMAD2
1 2 3 4 5 6 7 8 9 10 11 12
GAPDH of pSMAD2
1 2 3 4 5 6 7 8 9 10 11 12

Fig. S2A

O

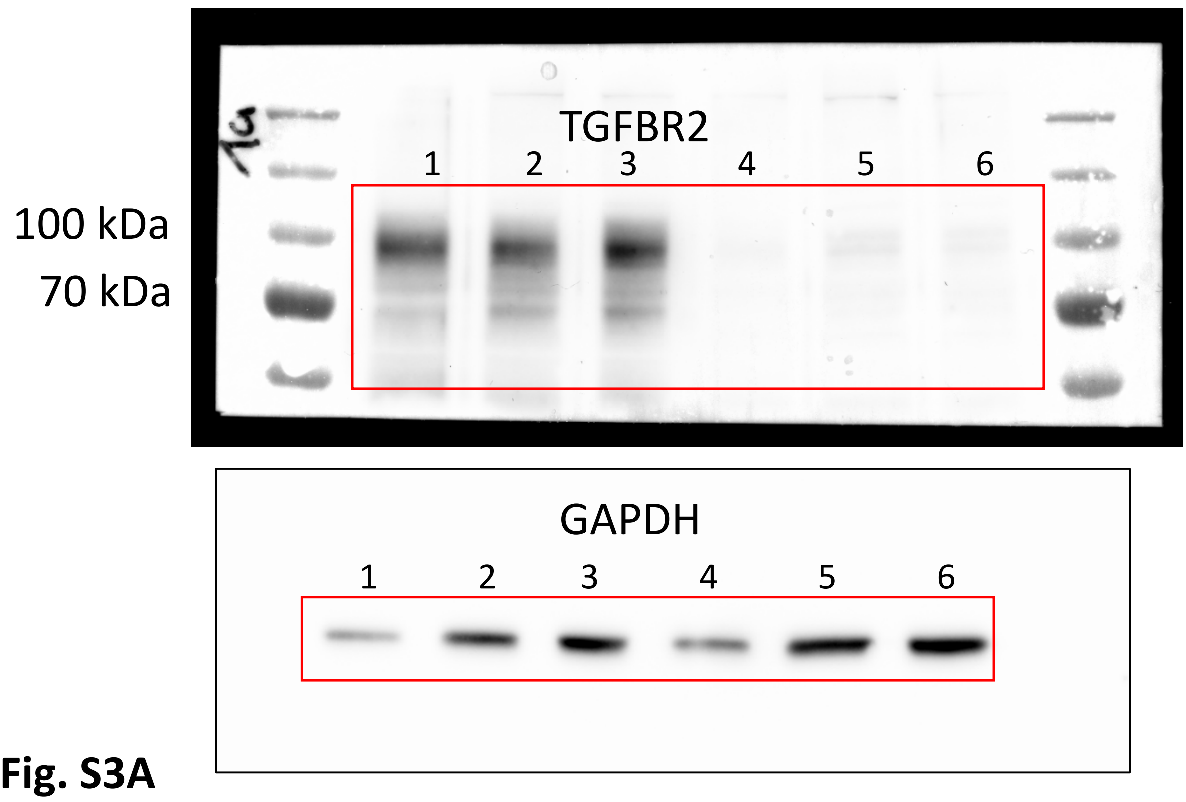

Fig. S3A

P

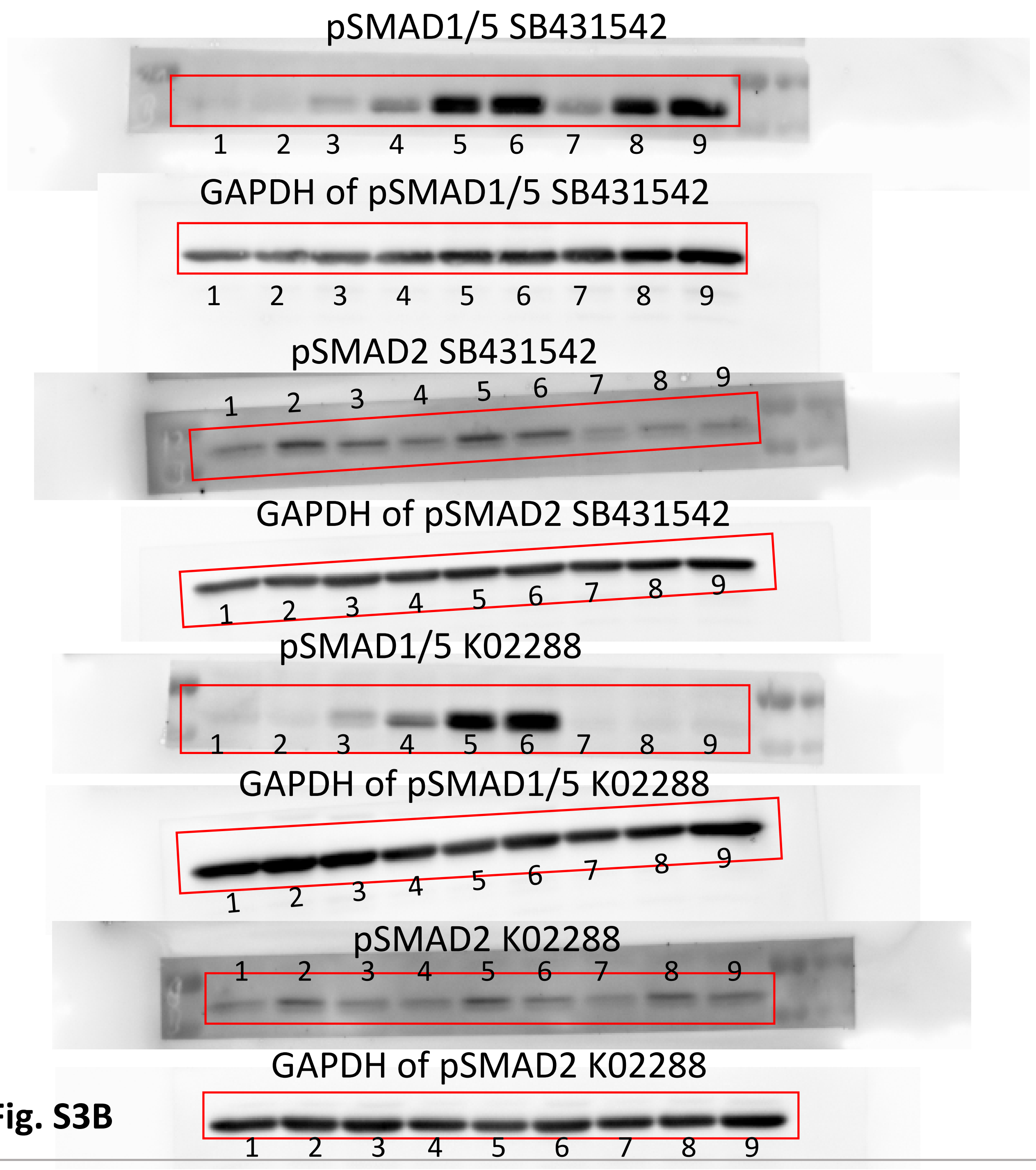

Fig. S3B
